# Tuning Intermolecular Interactions for Chiral Analysis: The Microwave Spectra and Molecular Structures of the Chiral Tag Candidates *cis*- and *trans*-2-Fluoro-3-(trifluoromethyl)oxirane and Their Gas-Phase Heterodimers with the Argon Atom

**DOI:** 10.1021/acs.jpca.4c05830

**Published:** 2024-10-16

**Authors:** Helen O. Leung, Mark D. Marshall, Jordan M. Aucoin, Jonah R. Horowitz

**Affiliations:** Department of Chemistry, Amherst College, P.O. Box 5000, Amherst, Massachusetts 01002-5000, United States

## Abstract

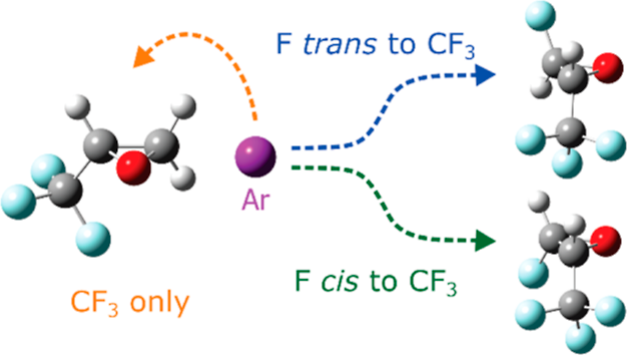

The *cis* and *trans* isomers of the chiral tagging candidate molecule, 2-fluoro-3-(trifluoromethyl)oxirane, as well as the lowest energy gas-phase heterodimer of each with the argon atom, are characterized via quantum chemistry calculations and microwave rotational spectroscopy from 5 to 18 GHz and their ground state, vibrationally averaged structures, are determined. Apart from the *cis*/*trans* nature of the ring substitution and small differences in the dihedral angle specifying the rotation of the trifluoromethyl group, the two oxirane molecules and their respective argon complexes each have remarkable structural similarity. In contrast, the binding mode of argon to the oxirane, while similar for the two complexes here, is distinct from those modes observed in previous argon-fluorooxirane species. The ability to tune the preferred mode of binding with differing levels of fluorine substitution may prove advantageous in applications of chiral tagging to a wide variety of analytes.

## 1 Introduction

Since its inception, the application of rotational spectroscopy to chiral analysis has proven to be both efficient and efficacious.^1,2^ Rotational spectroscopy is uniquely sensitive to the mass distribution of a molecular species. Enantiomers, however, have identical mass distribution and thus cannot be distinguished by their rotational spectra, yet they behave differently when they are in a chiral environment, present physiologically or otherwise. It is therefore important to be able to determine both the identity and quantity of an enantiomer in a sample, and this can be accomplished through intermolecular interactions. In the chiral tagging method, a chiral molecule (a “tag”) is used to form complexes with enantiomers, converting them into diastereomeric species with different moments of inertia, which give distinct rotational spectra. From these spectra, the absolute configurations of the enantiomers that form the complexes can be unambiguously established.^3−8^ Furthermore, the use of broadband, chirped pulse microwave spectroscopy allows simultaneous detection of many rotational transitions in a large range of frequencies, making it possible to quantitatively ascertain the relative amounts of the two complexes and thereby the original enantiomers.

Of course, the method described depends on suitable chiral tags. We have found that small fluorine-containing oxiranes, specifically, 2-(fluoromethyl)oxirane (FO),^9,10^ 2-(difluoromethyl)oxirane (DFO),^11^ and 2-(trifluoromethyl)oxirane (TFO),^12^ have features that make them potentially useful tags. (These molecules are also known as 3-fluoro-, 3,3-difluoro-, and 3,3,3-trifluoro-1,2-epoxypropane, respectively.) First, they can be easily introduced into the free-jet expansion of a broadband spectrometer. Second, they do not have features (such as a quadrupolar nucleus or internal rotation) that complicate the spectra of their complexes. Most importantly, they contain multiple functional groups, namely, electronegative O and F atoms and electropositive hydrogen atoms, that should enable them to bind to enantiomeric analytes readily. In fact, in self-tagging experiments, we have demonstrated that, with theoretical guidance, the spectra for the homochiral species of (FO)_2_,^13^ (DFO)_2_,^13^ and (TFO)_2_^14^ can be readily assigned. (The lowest energy heterochiral species of these dimers are not observed, consistent with theoretical prediction that they are nonpolar.) In another set of experiments, we collaborated with the Schnell group to employ both the microwave 3-wave mixing technique and chiral tag rotational spectroscopy to successfully quantify the enantiomeric excess of a sample of (*R*)- and (*S*)-styrene oxide using TFO as a chiral tag.^5^

Because different chiral analytes have different electronic and steric characteristics, it is desirable to have a number of chiral tags available to effectively form complexes with them. To this end, we continue to search for useful chiral tags, characterize their rotational spectra, and investigate how they interact via intermolecular interactions. In so doing, we also extend our understanding of the nature of intermolecular interactions. In the present work, we use samples that have an additional F atom in both *cis* and *trans* orientations relative to the trifluoromethyl group in TFO to give *cis*- and *trans*-2-fluoro-3-(trifluoromethyl)oxirane (also known as *cis*- and *trans*-1,3,3,3-tetrafluoro-1,2-epoxypropane, or in short, cFTFO and tFTFO). Our goal of this work is to explore how the presence of a fourth F atom and its location “tune” the properties of the other functional groups in the oxiranes. We report here our work on the rotational spectroscopy of these species and their molecular structures.

The use of argon as a carrier gas also affords us the opportunity to study how cFTFO and tFTFO interact with the argon atom via London dispersion forces. With the presence of one fluoromethyl group, the binding motif of argon to an oxirane depends on the number of F atoms in that group. Specifically, with more than one F atom, for DFO and TFO, argon locates on and interacts with the face of the epoxide ring opposite to that with the fluoromethyl group, the strongest interaction being with the O atom in both molecules.^11,12^ This is not the case with FO.^10^ Here, argon also interacts with the O atom and one of the C atoms in the epoxide ring, but this time, it is located on one side of the oxirane to make possible an interaction with the F atom in the fluoromethyl group. The different structures of the argon complexes of these three oxiranes have provided us with valuable information about the delicate balance of electrostatics and steric factors in intermolecular interactions, which we seek to expand in the present work that involves four fluorine atoms. We will show in the following that argon binds to cFTFO and tFTFO in a manner different from what we have observed so far in FO, DFO, and TFO.

## 2 Ab Initio Calculations

**Figure 1:**
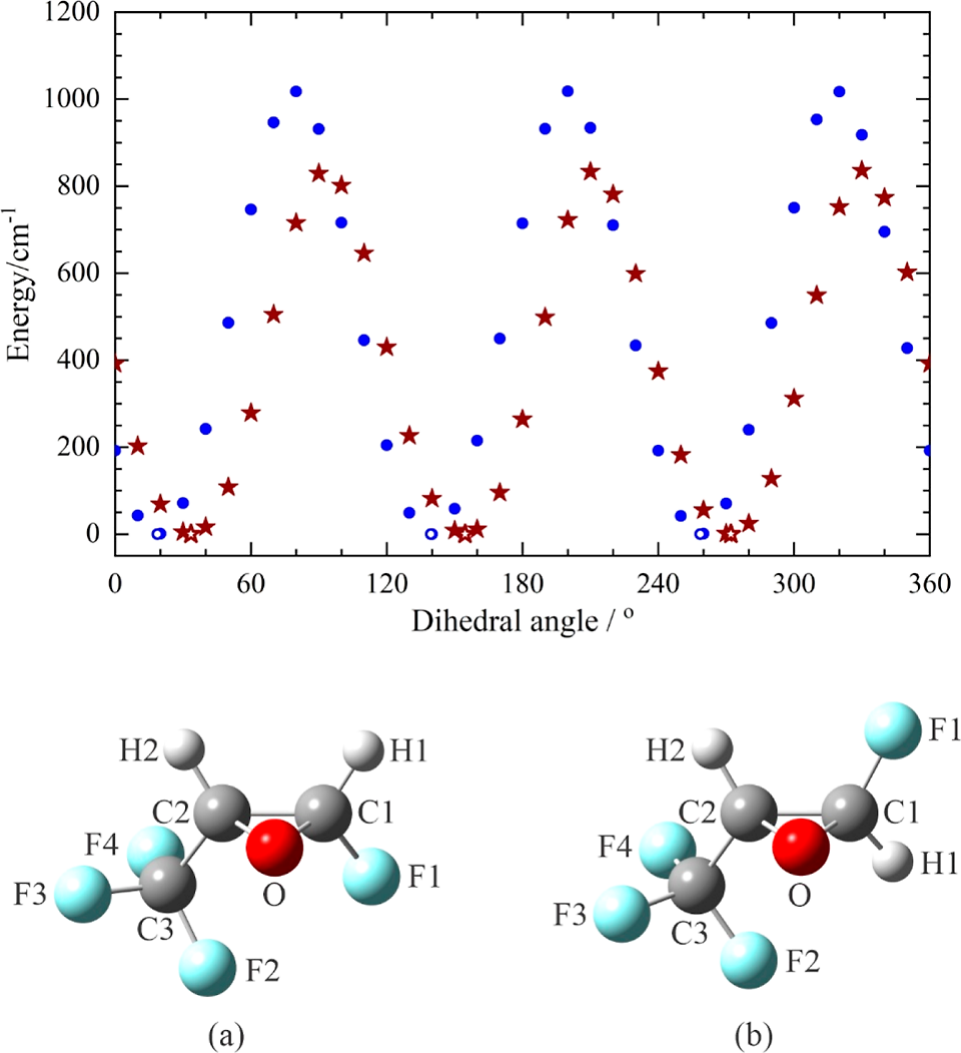
Relaxed scan of the C1C2C3F2 dihedral angle for each of the *cis* (brown star) and *trans* (blue circle solid) isomers of 2-fluoro-3-(trifluoromethyl)oxirane. One of the three equivalent optimized structures is shown in (a,b) for these isomers, respectively, with the corresponding energies of the minima set to 0, denoted by hollowed out symbols in the potential energy scan.

To facilitate our experimental work, we carry out ab initio calculations at the MP2/6-311++G(2d,2p) level with GAUSSIAN 16^15^ to determine the equilibrium structures of both cFTFO and tFTFO and the possible structures of their argon complexes. The structures of cFTFO and tFTFO and the barriers to CF_3_ internal rotation for these isomers are determined by scanning the C1C2C3F2 dihedral angle from 0 to 360° in steps of 10° while relaxing all other structural parameters in each molecule. The resulting potential energy curve and the optimized structure for each species (assigned to have an energy of 0) at one of the three equivalent minima, as well as the atom numbering schemes, are shown in Figure 1, while the structural parameters are listed in Table 1.

**Table 1:** Structural Parameters for *Cis*- and *Trans*-2-Fluoro-3-(trifluoromethyl)oxirane Using Ab Initio Calculation and a Structure Fit to the Moments of Inertia of 5 Isotopologues of Each Molecule

	cFTFO		tFTFO	
	theory	experiment^a^	theory	experiment^a^
C1–O/Å	1.3992	1.3843(31)	1.3981	1.4087(82)
C2–O/Å	1.4507	1.4470(61)	1.4503	1.4699(51)
C1–C2/Å	1.4576	1.4748(78)	1.4558	1.4866(72)
C2–C3/Å	1.5040	1.4966(68)	1.4989	1.4690(75)
C1–H1/Å	1.0796	[1.0796]	1.0787	[1.0787]
C1–F1/Å	1.3509	[1.3509]	1.3538	[1.3538]
C2–H2/Å	1.0810	[1.0810]	1.0802	[1.0802]
C3–F2/Å	1.3279	[1.3279]	1.3392	[1.3392]
C3–F3/Å	1.3458	[1.3458]	1.3399	[1.3399]
C3–F4/Å	1.3463	[1.3463]	1.3443	[1.3443]
∠C1C2O/deg	57.516	56.55(29)	57.512	56.91(42)
∠C2OC1/deg	61.492	62.74(28)	61.442	62.15(28)
∠OC1C2/deg	60.992	60.71(18)	61.046	60.95(24)
∠H1C1C2/deg	122.427	[122.427]	124.100	[124.100]
∠F1C1C2/deg	120.271	119.85(24)	117.855	118.41(32)
∠H2C2C1/deg	120.411	[120.411]	119.062	[119.062]
∠C3C2C1/deg	122.069	122.14(30)	121.279	121.38(38)
∠F2C3C2/deg	113.652	113.85(35)	111.906	112.07(36)
∠F3C3C2/deg	109.755	109.96(28)	111.231	111.39(26)
∠F4C3C2/deg	109.135	109.34(20)	109.524	109.68(20)
∠H1C1C2O/deg	–104.822	[−104.822]	104.779	[104.779]
∠F1C1C2O/deg	105.217	105.00(26)	–105.173	–102.37(62)
∠H2C2C1O/deg	103.050	[103.050]	103.386	[103.386]
∠C3C2C1O/deg	–102.532	[−102.532]	–101.756	[−101.756]
∠F2C3C2C1/deg	33.550	[33.550]	18.672	[18.672]
∠F3C3C2C1/deg	154.624	[154.624]	139.486	[139.486]
∠F4C3C2C1/deg	–87.943	[−87.943]	–101.366	[−101.366]
*A*/MHz	3532		4066	
*B*/MHz	1664		1322	
*C*/MHz	1486		1249	
|μ_a_|/D	1.098		0.281	
|μ_b_|/D	0.951		1.041	
|μ_c_|/D	2.595		0.061	

a 1σ standard deviations in the parameters are given in parentheses. The parameters without uncertainties are fixed to the ab initio values and are enclosed in square brackets.

The barrier to CF_3_ internal rotation for tFTFO is 1020 cm^–1^, which is 160 cm^–1^ higher than that for cFTFO. The values for these barriers are rather high, suggesting that CF_3_ is not likely to exhibit free internal rotation in either molecule. The values of the C1C2C3F2 dihedral angle are 33.55° and 18.67°, respectively, for cFTFO and tFTFO. The larger value of the angle in cFTFO serves to relieve repulsive steric interactions between F1 and F2 while allowing favorable intramolecular interactions between H2 and the two other F atoms in the fluoromethyl group. In the optimized structure, the F1–F2 interaction length is 2.7036 Å, which is shorter than the optimal van der Waals contact distance of 2.94 Å^16^ but the H2–F3 and H2–F4 interaction lengths are 2.5724 and 2.6967 Å, respectively, indicating strong attractive interactions. For tFTFO, the smaller C1C2C3F2 dihedral angle allows four sets of attractive intramolecular interactions: H1–F2, 2.5714 Å; H2–F3, 2.7272 Å; H2–F4, 2.6287 Å; and H2–F1, 2.6254 Å, with the H1–F2 interaction being the shortest and thus the strongest. Additionally, the F2–O distances are 2.7703 Å for cFTFO and 2.8096 Å for tFTFO. Both of these values are smaller than the F–O van der Waals contact of 2.99 Å, making the interaction in cFTFO less favorable. (The atomic coordinates for the equilibrium structures of both isomers can be found in Supporting Information.) Because of the multiple favorable attractive interactions (and fewer unfavorable interactions) in tFTFO, it is not surprising that the equilibrium energy for tFTFO is 825 cm^–1^ lower than that of cFTFO.

A comparison between the equilibrium structural parameters in both isomers (Table 1) shows that they are very similar. The corresponding bond lengths in cFTFO and tFTFO differ from each other by only 0.0004–0.0113 Å. The one notable difference is the orientation of the CF_3_ group, as reflected in the three C1C2C3F*n* (*n* = 2, 3, and 4) dihedral angles and mentioned earlier. Among the heavy atoms, there are minor differences in the F1C1C2, F2C3C2, and F3C3C2 angles, which are, respectively, 2.42° greater, 1.75° greater, and 1.48° smaller in cFTFO than in tFTFO. The greater values of the F1C1C2 and F2C3C2 angles in cFTFO serve to alleviate the F1–F2 repulsion. If they were to assume the same values as those for tFTFO, then the F1–F2 distance would decrease from 2.7036 Å to an even more unfavorable value of 2.6149 Å. On the other hand, the smaller F3C3C2 angle in cFTFO allows F3 and H2 to have a more favorable interaction length of 2.5724 Å instead of a longer 2.5996 Å if it were to assume the same value as in tFTFO. All in all, the differences in the equilibrium structures in the two isomers, not surprisingly, is a result of a balance among various intramolecular interactions. The rotational constants and magnitudes of the dipole moment components along the inertial axes are listed in Table 1 for both isomers. cFTFO has a large dipole moment of 2.974 D, and should have very strong *a*- and *b*-type transitions, and even stronger *c*-type transitions. The dipole moment, 1.080 D, for tFTFO is much smaller; we expect strong *b*-type transitions, much weaker *a*-type transitions, followed by *c*-type transitions.

**Figure 2:**
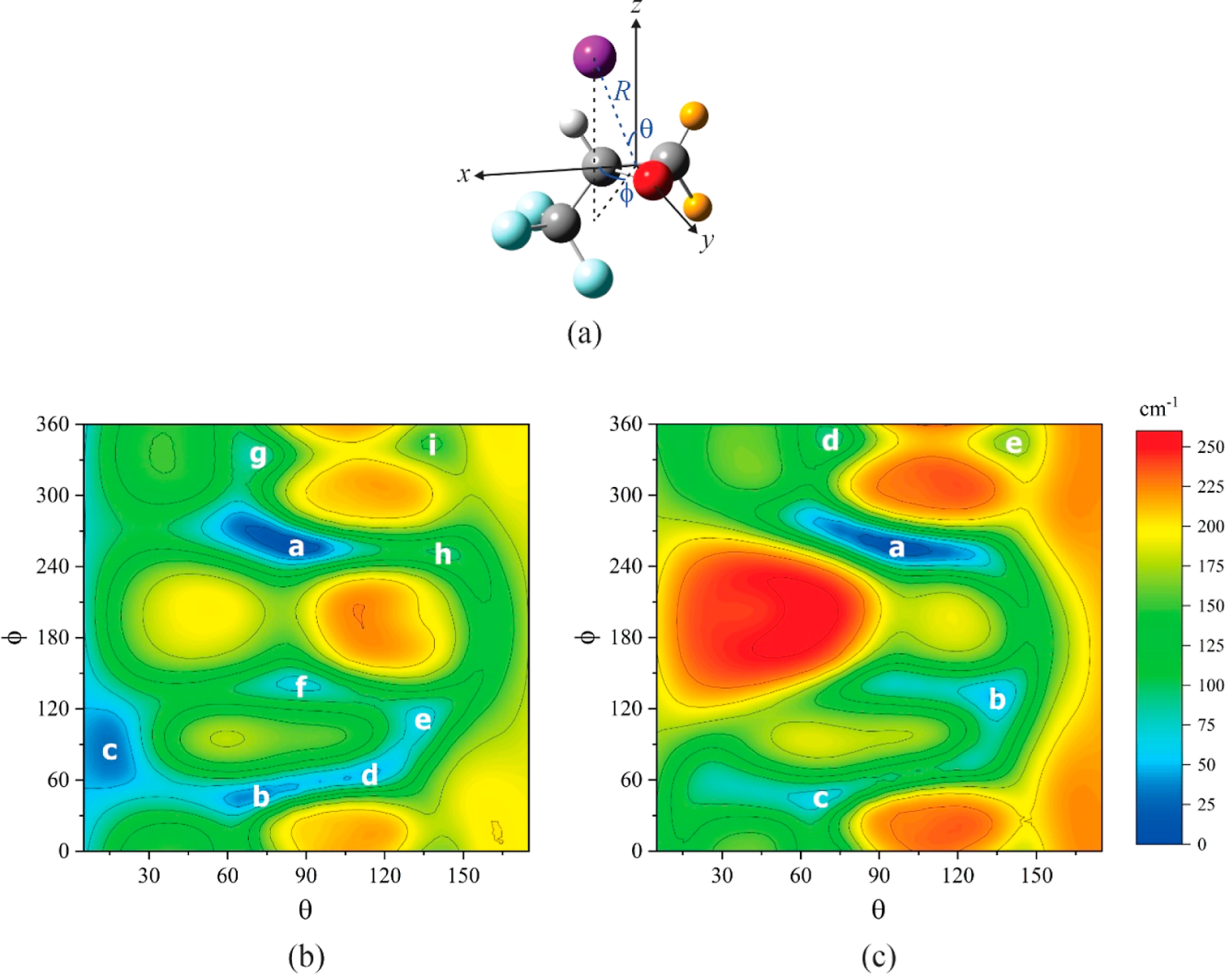
(a) Coordinate system that defines the position of argon with respect to cFTFO and tFTFO, as described in Section 2. Orange spheres represent a pair of H and F atoms appropriate to the oxirane in question. The potential contours due to argon interacting with (b) cFTFO and (c) tFTFO at various polar (θ) and azimuthal (ϕ) angles while relaxing its distance from the origin (*R*) are plotted using the same energy scale. Atom colors: C, dark gray; H, light gray; O, red; F, light blue; and Ar, purple.

**Figure 3:**
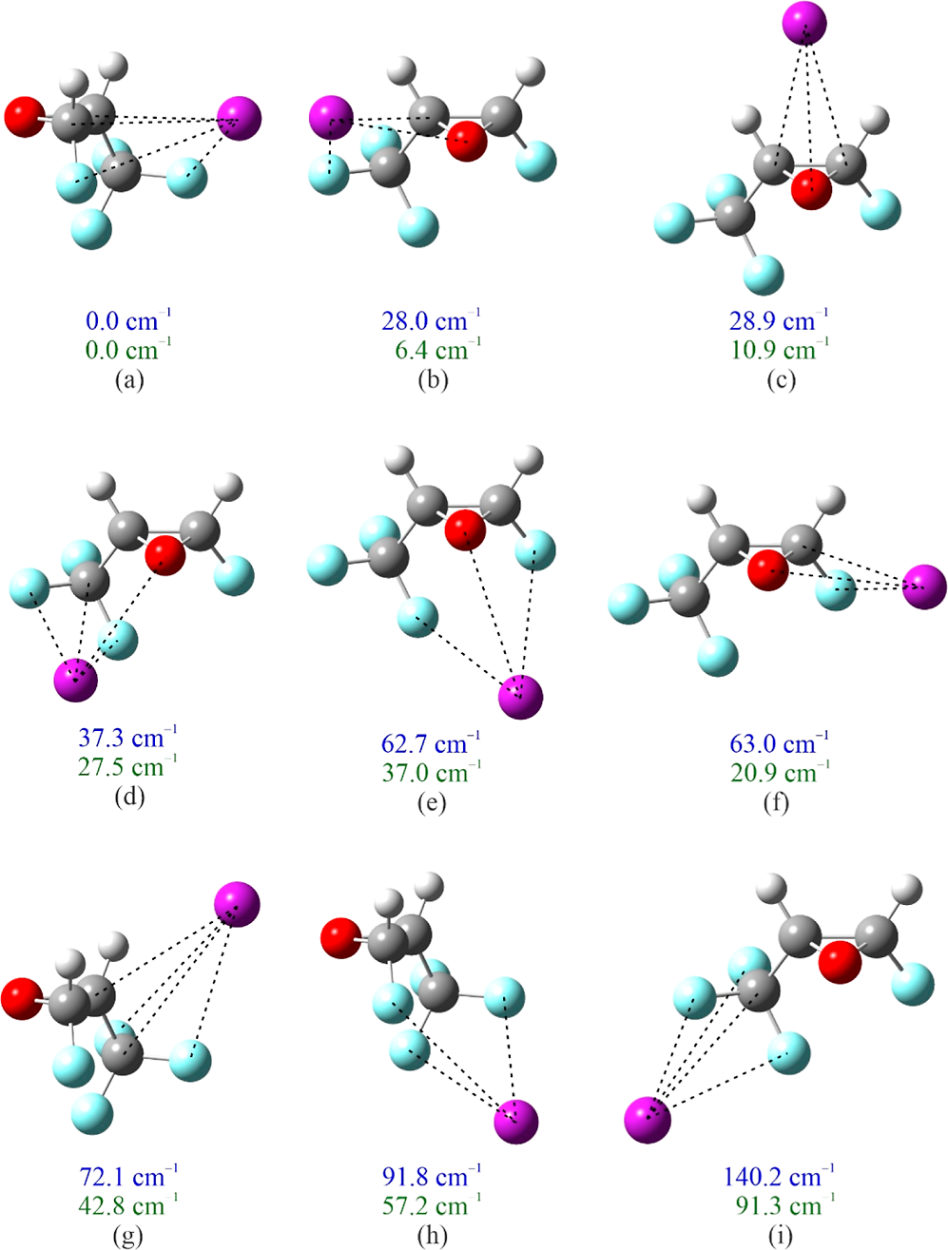
Optimized structures of Ar-cFTFO (without BSSE correction) corresponding to the minima found in the potential contour displayed in Figure 2b. The more important intermolecular interactions (arbitrarily chosen to have a distance no more than 10% longer than the sum of the van der Waals radii of the respective atoms) are indicated using dashed lines. The upper (blue) of each pair of numbers is the relative equilibrium energy, without BSSE correction, of each structure, and the lower (green) of each pair is the relative energy, including both BSSE and zero-point corrections. Atom colors: C, dark gray; H, light gray; O, red; F, light blue; Ar, purple.

**Figure 4:**
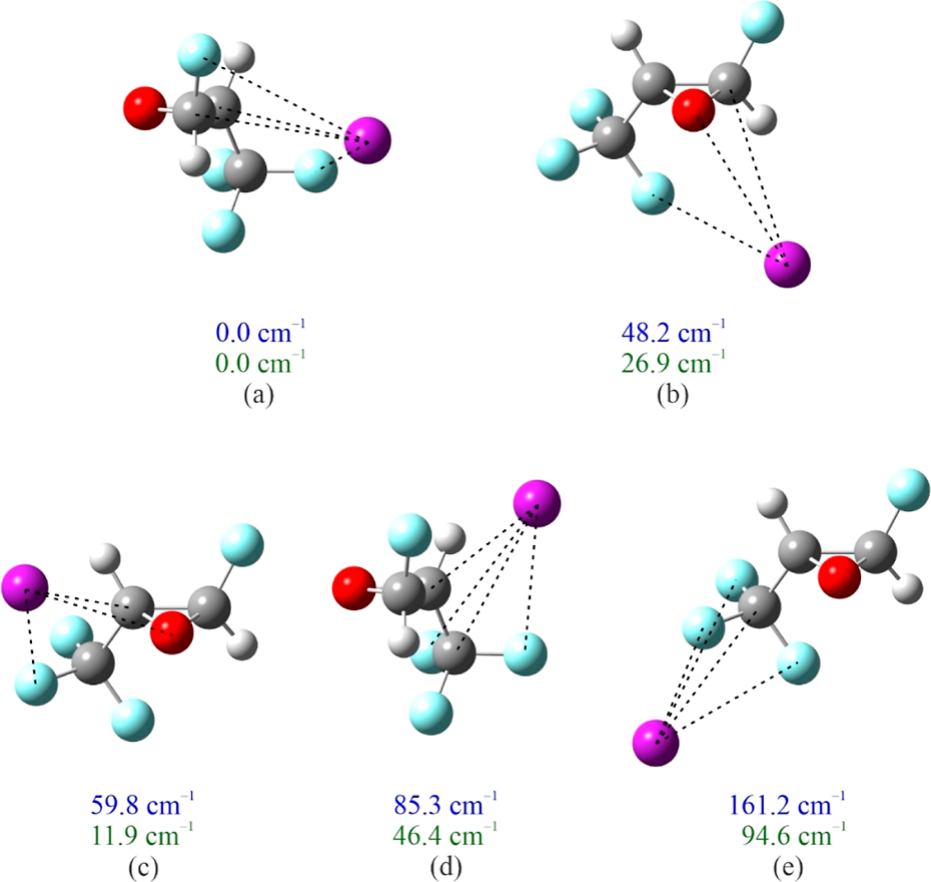
Optimized structures of Ar-tFTFO (without BSSE correction) corresponding to the minima found in the potential contour displayed in Figure 2c. The more important intermolecular interactions (arbitrarily chosen to have a distance no more than 10% longer than the sum of the van der Waals radii of the respective atoms) are indicated using dashed lines. The upper (blue) of each pair of numbers is the relative equilibrium energy, without BSSE correction, of each structure, and the lower (green) of each pair is the relative energy including both BSSE and zero-point corrections. Atom colors: C, dark gray; H, light gray; O, red; F, light blue; and Ar, purple.

To investigate the interaction potential energy between argon and each of the monomers, we fix the oxirane to its equilibrium structure (as described in Table 1) and place the origin of an axis system in the middle of the C–C bond in the epoxide ring. The *x* axis is along the bond, with the positive direction pointing away from C1, while the *z* axis is perpendicular to the epoxide ring, with the positive direction pointing away from the trifluoromethyl group (Figure 2a). An argon atom is then positioned at various polar (θ) and azimuthal (ϕ) angles, while its distance from the origin (*R*) is optimized. The potential energy contours deriving from a scan of θ from 5 to 175° and ϕ from 0 to 360°, each in steps of 10°, are shown in Figure 2b for Ar-cFTFO and in Figure 2c for Ar-tFTFO. Nine and five minima, respectively, are found; their structures are optimized and labeled alphabetically in order of increasing equilibrium energy (Figures 3 and 4). The interaction lengths between Ar and the heavy atoms in cFTFO in each Ar-cFTFO isomer are listed in Table 2, with their rotational constants, dipole moments, and energies listed in Table 3. Equivalent information for the Ar-tFTFO isomers is listed in Tables 4 and 5. To view the important interactions, we outline, in Figures 3 and 4, those interaction lengths that are no more than 10% longer than the van der Waals contact lengths. (The sums of van der Waals radii are Ar–C, 3.58 Å; Ar–O, 3.40 Å; and Ar–F, 3.35 Å.)^16^

**Table 2:** Interaction Lengths in Ångstroms between Ar and Heavy Atoms in Nine Isomers of Ar-cFTFO Obtained from Ab Initio Calculations at the MP2/6-311++G(2d,2p) Level without and with BSSE Correction (BSSEC)^a^

	without BSSEC	with BSSEC	without BSSEC	with BSSEC	without BSSEC	with BSSEC	without BSSEC	with BSSEC	without BSSEC	with BSSEC
	structure (a)		structure (b)		structure (c)		structure (d)		structure (e)	
Ar–C1	3.533	3.690	4.576	4.694	3.710	3.848	4.695	4.857	4.102	4.255
Ar–C2	3.824	3.932	3.639	3.756	3.684	3.834	4.119	4.286	4.487	4.686
Ar–C3	4.218	4.380	4.137	4.328	5.112	5.261	3.912	4.131	4.425	4.638
Ar–O	4.804	4.938	3.374	3.549	3.533	3.690	3.441	3.572	3.527	3.709
Ar–F1	3.551	3.811	5.647	5.820	5.012	5.145	5.054	5.251	3.629	3.747
Ar–F2	4.801	5.038	4.790	5.056	5.980	6.136	3.580	3.834	3.427	3.628
Ar–F3	5.344	5.457	3.484	3.688	5.404	5.550	3.495	3.683	4.961	5.186
Ar–F4	3.491	3.645	5.227	5.362	5.716	5.848	5.217	5.440	5.501	5.699
	structure (f)		structure (g)		structure (h)		structure (i)		experiment	
Ar–C1	3.495	3.644	5.155	5.283	4.438	4.636	6.238	6.469	3.57957(23)	
Ar–C2	4.634	4.782	3.918	4.074	4.703	4.919	5.310	5.557	3.83870(30)	
Ar–C3	5.643	5.828	3.903	4.109	4.071	4.284	3.808	4.053	4.20686(10)	
Ar–O	3.377	3.527	5.184	5.332	5.321	5.527	6.123	6.359	4.82100(35)	
Ar–F1	3.561	3.754	6.065	6.210	3.429	3.608	6.035	6.240	3.60848(26)	
Ar–F2	5.380	5.597	5.205	5.415	3.549	3.748	3.557	3.772	4.80122(39)	
Ar–F3	6.447	6.621	3.565	3.790	5.227	5.431	3.552	3.801	5.32318(21)	
Ar–F4	6.569	6.750	3.541	3.734	3.529	3.728	3.564	3.794	3.45850(23)	

a The experimental results come from a fit to the moments of inertia of four isotopologues of the complex. 1σ standard deviations in the experimental results are given in parentheses.

**Table 3:** Rotational Constants, Dipole Moment Components, and Relative Equilibrium and Zero-Point-Corrected Energies for Nine Isomers of the Complex between Argon and cFTFO Obtained from Ab Initio Calculations at the MP2/6-311++G(2d,2p) Level without and with BSSEC

	without BSSEC	with BSSEC	without BSSEC	with BSSEC	without BSSEC	with BSSEC	without BSSEC	with BSSEC	without BSSEC	with BSSEC
	structure (a)		structure (b)		structure (c)		structure (d)		structure (e)	
*A*/MHz	1661	1630	1693	1689	1867	1860	1584	1583	1734	1746
*B*/MHz	796	748	751	701	597	568	823	761	749	695
*C*/MHz	644	612	584	553	512	491	645	604	642	604
|μ_a_|/D	1.887	2.048	0.541	0.780	2.591	2.607	1.584	1.466	1.500	1.502
|μ_b_|/D	0.354	0.346	1.257	1.273	0.311	0.300	0.819	0.885	2.391	2.401
|μ_c_|/D	2.232	2.110	2.586	2.524	1.529	1.508	2.401	2.446	1.085	1.030
*E*_equil_^a^^,^^b^/cm^–1^	0.0	0.0	28.0	7.5	28.9	6.6	37.3	31.4	62.7	42.3
*E*_zpe_^a^^,^^c^/cm^–1^	0.0	0.0	25.5	6.4	38.6	10.9	32.7	27.5	57.5	37.0
	structure (f)		structure (g)		structure (h)		structure (i)			
*A*/MHz	3070	3082	1979	1922	1669	1669	2828	2781		
*B*/MHz	505	479	633	601	812	748	557	519		
*C*/MHz	486	461	570	540	617	580	525	490		
|μ_a_|/D	0.671	0.743	1.262	1.383	1.011	1.014	1.960	1.996		
|μ_b_|/D	0.038	0.016	2.644	2.602	1.193	1.190	1.147	1.133		
|μ_c_|/D	2.835	2.827	0.423	0.366	2.527	2.526	1.937	1.904		
*E*_equil_^a^^,^^b^/cm^–1^	63.0	26.2	72.1	46.4	91.8	62.3	140.2	95.5		
*E*_zpe_^a^^,^^c^/cm^–1^	61.9	20.9	71.3	42.8	84.9	57.2	134.3	91.3		

a The energies for each calculation method are given relative to the values obtained using the same calculation method for the most stable isomer. These are −1116.056610, −1116.055768, −1116.000910, and −1115.999982 hartree for *E*_equil_, *E*_equil_ + BSSE, *E*_zpe_, and *E*_zpe_ + BSSE, respectively.

b This equilibrium energy is determined by using the equilibrium structure of cFTFO and optimizing the intermolecular degrees of freedom with argon, without and with BSSE correction, respectively.

c The equilibrium structure and energy of the complex are calculated while allowing a full relaxation of the complex geometry, including the structural parameters of cFTFO. (These differ slightly from those found when cFTFO is fixed to its equilibrium structure, as in the preceding line.) A harmonic zero-point correction to the energy is calculated for this structure, which is included in both columns for each isomer, as is a counterpoise correction for BSSE, which is included in the second column for each.

**Table 4:** Interaction Lengths in Ångstroms between Ar and Heavy Atoms in Five Isomers of Ar-tFTFO Obtained from Ab Initio Calculations at the MP2/6-311++G(2d,2p) Level without and with BSSEC^a^

	without BSSEC	with BSSEC	without BSSEC	with BSSEC	without BSSEC	with BSSEC
	structure (a)		structure (b)		structure (c)	
Ar–C1	3.532	3.680	3.730	3.862	4.570	4.712
Ar–C2	3.844	4.013	4.394	4.578	3.613	3.768
Ar–C3	4.064	4.228	4.429	4.646	4.174	4.342
Ar–O	4.806	4.963	3.477	3.638	3.434	3.577
Ar–F1	3.604	3.726	4.712	4.798	5.019	5.132
Ar–F2	4.229	4.372	3.478	3.693	5.103	5.273
Ar–F3	5.348	5.513	4.934	5.165	3.537	3.699
Ar–F4	3.483	3.635	5.531	5.738	5.047	5.204
	structure (d)		structure (e)		experiment	
Ar–C1	5.247	5.390	6.199	6.435	3.58260(43)	
Ar–C2	3.896	4.056	5.308	5.555	3.73647(20)	
Ar–C3	3.877	4.083	3.809	4.056	4.05993(26)	
Ar–O	5.000	5.142	6.087	6.325	4.80479(41)	
Ar–F1	5.640	5.741	7.428	7.667	3.58893(45)	
Ar–F2	5.196	5.406	3.558	3.780	4.44592(28)	
Ar–F3	3.542	3.749	3.557	3.794	5.28166(46)	
Ar–F4	3.565	3.774	3.562	3.798	3.40696(53)	

a The experimental results come from a fit to the moments of inertia of four isotopologues of the complex. 1σ standard deviations in the experimental results are given in parentheses.

**Table 5:** Rotational Constants, Dipole Moment Components, and Relative Equilibrium and Zero-Point-Corrected Energies for Five Isomers of the Complex between Argon and tFTFO Obtained from Ab Initio Calculations at the MP2/6-311++G(2d,2p) Level without and with BSSEC

	without BSSEC	with BSSEC	without BSSEC	with BSSEC	without BSSEC	with BSSEC	without BSSEC	with BSSEC	without BSSEC	with BSSEC
	structure (a)		structure (b)		structure (c)		structure (d)		structure (e)	
*A*/MHz	1454	1444	1369	1384	1282	1282	1496	1449	3641	3605
*B*/MHz	857	806	781	721	822	771	696	662	471	441
*C*/MHz	606	579	571	541	573	547	550	523	465	436
|μ_a_|/D	1.093	1.097	0.379	0.352	0.714	0.704	0.045	0.052	0.206	0.201
|μ_b_|/D	0.336	0.288	0.469	0.472	0.316	0.328	0.297	0.299	1.025	1.014
|μ_c_|/D	0.177	0.186	0.814	0.830	0.666	0.679	1.028	1.028	0.251	0.296
*E*_equil_^a^^,^^b^/cm^–1^	0.0	0.0	48.2	19.0	59.8	12.4	85.3	46.8	161.2	96.7
*E*_zpe_^a^^,^^c^/cm^–1^	0.0	0.0	52.9	26.9	59.9	11.9	84.1	46.4	154.1	94.6

a The energies for each calculation method are given relative to the values obtained using the same calculation method for the most stable isomer. These are −1116.060452, −1116.059529, −1116.004820, and −1116.003826 hartree for *E*_equil_, *E*_equil_ + BSSE, *E*_zpe_, and *E*_zpe_ + BSSE, respectively.

b This equilibrium energy is determined by using the equilibrium structure of tFTFO and optimizing the intermolecular degrees of freedom with argon, without and with BSSE correction, respectively.

c The equilibrium structure and energy of the complex are calculated while allowing a full relaxation of the complex geometry, including the structural parameters of tFTFO. (These differ slightly from those found when tFTFO is fixed to its equilibrium structure, as in the preceding line.) A harmonic zero-point correction to the energy is calculated for this structure, which is included in both columns for each isomer, as is a counterpoise correction for BSSE, which is included in the second column for each.

Four of the five binding modes in Ar-tFTFO are also present in Ar-cFTFO. Specifically, the Ar-tFTFO isomers (a), (c), (d), and (e) have binding motifs similar to those of the Ar-cFTFO isomers (a), (b), (g), and (i). One of these motifs, that in isomer (b) of Ar-cFTFO and isomer (c) of Ar-tFTFO, has been observed for Ar-FO.^10^ The motifs for five Ar-cFTFO isomers, (c), (d), (e), (f), and (h) have no Ar-tFTFO counterparts, but that for isomer (c) has been observed for Ar-DFO^11^ and Ar-TFO.^12^ The motif for the lowest energy isomers of Ar-cFTFO and Ar-tFTFO is the same, and one that has not been observed experimentally: the Ar atom is positioned in the “back” of the epoxide ring away from the O atom, interacting with C1, C2, F1, and F4 of each oxirane. The interaction lengths of Ar with C1, C2, and F4 in Ar-cFTFO and Ar-tFTFO for this binding mode differ by only 0.001 to 0.02 Å. Because of the location of F1, Ar is closer to it in cFTFO than in tFTFO by 0.053 Å.

We have found that in our studies of complexes of oxiranes and haloolefins, the energy ordering of isomers may change when corrected for basis set superposition error (BSSE).^17^ We therefore proceed to do so for all argon isomers of cFTFO and tFTFO, and the resulting interaction lengths between Ar and heavy atoms are listed in Tables 2 and 4, with their rotational constants, dipole moment components, and energies listed in Tables 3 and 5. (The atomic coordinates for the equilibrium structures of all isomers of Ar-cFTFO and Ar-tFTFO, with or without BSSE correction, are available in the Supporting Information.) Here, the energy ordering does change for each set of isomers of Ar-cFTFO and Ar-tFTFO, but isomer (a) remains the lowest energy species in each case. This is also the case when a zero-point correction is applied to each species, with or without BSSE correction (Tables 3 and 5). The energy differences between different isomers, however, are small. For example, with both BSSE and zero-point corrections, isomer (a) and the next lowest energy isomer [isomer (b) for Ar-cFTFO or isomer (c) for Ar-tFTFO] differ by only 6 or 12 cm^–1^. With the understanding that theory, at least at the level we use, may not be able to distinguish energies that are so similar, we look for isomer (a) in our spectrum for each complex, but keeping in mind that other isomers may also be populated under our experimental conditions. With different rotational constants and dipole moment components, we should have no difficulty in identifying the binding motifs of species present in our spectra.

## 3 Experiment

Two microwave spectrometers are employed for this work. A broadband, chirped pulse spectrometer is used to collect the spectrum of each of cFTFO and tFTFO in argon.^18−20^ We are able to identify and analyze transitions due to five isotopologues of each monomer and the most abundant species of its argon complex. It is possible to observe transitions due to the three isotopologues of the argon complex singly substituting with ^13^C in the spectrum obtained for cFTFO but not in the spectrum for tFTFO in argon. We thus turn to the more sensitive Balle-Flygare narrowband spectrometer to search for transitions due to the ^13^C isotopologues of Ar-tFTFO.

Samples of cFTFO and tFTFO, both in racemic form, are obtained from SynQuest Laboratories. For cFTFO, the liquid sample was placed in a sample cylinder. frozen by placing the sealed cylinder in liquid nitrogen and pumping out the air above the sample. After thawing, the vapor pressure was used to prepare a 1/2% mixture of cFTFO in argon. More conveniently, tFTFO is supplied as a gas that is used directly to prepare an 3/4–1% sample in argon. The backing pressure used for both samples is approximately 1 to 2 atm.

The gas mixture is introduced into the broadband instrument through two pulsed valves operating at 4 Hz, each with a 0.8 mm diameter nozzle. The molecules are polarized using a chirped microwave pulse of 4 μs duration and 20–25 W of power. Allowing 0.5 μs for the polarization pulse to dissipate, the resulting free induction decay (FID) is then digitized at 50 Gs s^–1^ for 20 μs. Ten FIDs are collected during each 800 μs opening of the pulsed valves, and 1,356,000 to 1,485,000 FID’s (tFTFO) or 1,521,000 to 1,818,000 (cFTFO) are averaged for each of the three frequency segments that are assembled to cover the entire 5.6–18.1 GHz bandwidth of the instrument. As described previously,^19^ the average is Fourier transformed to give a frequency domain spectrum with a resolution element of 11.92 kHz and typical line widths (fwhm) of 125 kHz. Using this procedure, the estimated uncertainty in the frequency measurement is 5–10 kHz.

The Balle–Flygare spectrometer^19,21^ operates in the 5–21 GHz range. It has one pulsed valve mounted parallel to the resonator axis. The background-corrected time domain signals are digitized for 1024 data points and zero-filled to a 2048-point record length before Fourier transformation to give frequency domain signals with a resolution of 4.8 kHz. Each transition appears as a Doppler doublet, and the transition frequency is the mean of the doublet frequencies, which gives a measurement uncertainty of ∼2 kHz.

## 4 Results

### 4.1 Spectral Analysis

#### 4.1.1 cFTFO and tFTFO

No internal rotation of the CF_3_ group is evident in the tFTFO spectrum, and this is also the case for the majority of transitions in the cFTFO spectrum. However, for some transitions with high *J* (typically 10 or higher) and/or high *K*_a_ (typically 5 or higher), the lines due to cFTFO appear broadened and sometimes split into two, although these are often not well resolved. The cause of this splitting is not clear. The expansion conditions used in the experiment (1–2 atm in argon) are not particularly favorable for higher cluster formation in the centerline of the jet expansion, and all attempts to find transitions due to dimers or higher clusters in the spectrum of cFTFO have, to this point, been unsuccessful. Because of the large mass of the rotor, attributing this effect to CF_3_ hindered internal rotation is also problematic, although we do note that there is a lower barrier to rotation in cFTFO than in tFTFO. Furthermore, attempts to model the splitting with XIAM^22^ actually gave splittings much larger than those observed and that were, in fact, physically unreasonable. In the end, we only include transitions that do not show these effects in our analysis, which is sufficient for the purposes of this study.

**Figure 5:**
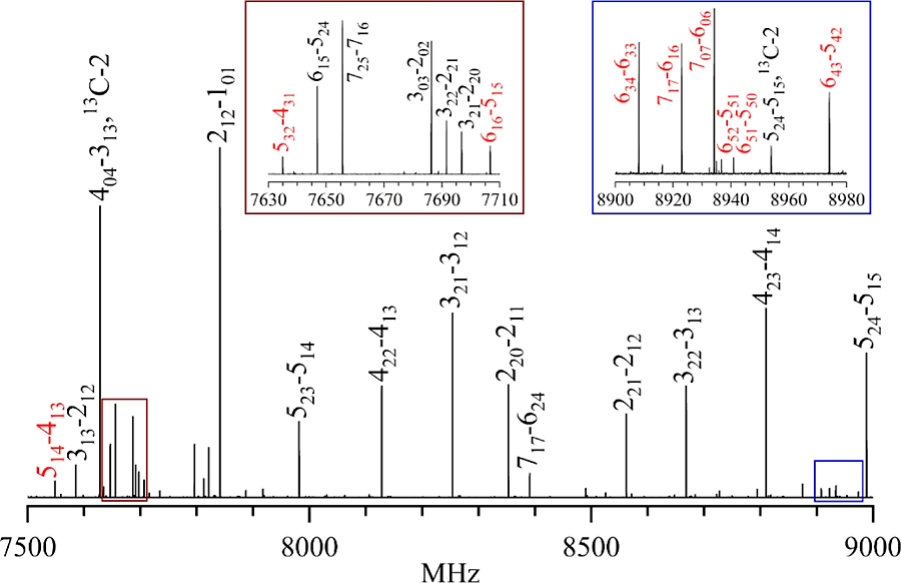
1500 MHz segment of the chirped pulse spectrum obtained from a sample of tFTFO in Ar. The transitions labeled in black, except for two due to the ^13^C-2 isotopologue, come from the most abundant species of the tFTFO monomer. Transitions labeled in red are due to the most abundant isotopologue of Ar-tFTFO. Two regions are magnified and are displayed as insets.

The broadband spectra are assigned using Kisiel’s AABS program.^23^ For the most abundant isotopologues of cFTFO and tFTFO, we have analyzed over 235 *a*-, *b*-, and *c*-type transitions, with line intensities matching the theoretically predicted dipole moment components (Table 1). These transitions cover large *J* (1–20 or higher) and *K*_a_ (0–9) ranges. Fewer transitions due to the minor isotopologues in natural abundance (^18^O and ^13^C-containing) are analyzed for each isomer: 68–140 for cFTFO and 31–92 for tFTFO. The quality of the spectra can be seen in Figure 5 by using the tFTFO sample in argon as an example. The 1500 MHz region shows several *a*-type transitions and a number of *b*-type transitions due to the most abundant isotopologue of tFTFO, and also two transitions due to a ^13^C isotopologue.

The spectroscopic constants for each species are determined using the Watson *S*-reduced Hamiltonian^24,25^ in the I^*r*^ representation and Pickett’s nonlinear SPFIT program,^26^ and are listed in Tables 6 and 7. In addition to the rotational constants, we are able to determine 4–5 quartic centrifugal distortion constants for all species. The magnitudes of the centrifugal distortion constants for cFTFO are generally much greater than those for tFTFO. Using the most abundant isotopologues as an example, the magnitudes of *D*_J_, *D*_JK_, *D*_K_, *d*_1_, and *d*_2_ for cFTFO are greater than those for tFTFO by factors of 3.5, 1.2, 2.4, 5.1, and 7.3, which indicate that the bonding in cFTFO is less rigid and lend credence to the theoretical finding and experimental observation that there is greater ease for CF_3_ internal rotation in this molecule than in tFTFO. The rms deviation of each fit is 4–6 kHz, commensurate with the measurement precision of the broadband spectrometer. Tables of observed and calculated transition frequencies with assignments for all isotopologues studied are provided in the Supporting Information.

**Table 6:** Spectroscopic Constants (in MHz, Unless as Otherwise Noted) for Five Isotopologues of cFTFO^a^

	c-CHFCH(CF_3_)O	c-^13^CHFCH(CF_3_)O	c-CHF^13^CH(CF_3_)O	c-CHFCH(^13^CF_3_)O	c-CHFCH(CF_3_)^18^O
*A*	3554.89277(18)	3548.35492(32)	3533.39607(31)	3555.12229(29)	3474.79946(59)
*B*	1657.274725(87)	1640.99656(15)	1654.81865(17)	1653.24570(13)	1639.62583(33)
*C*	1482.449327(86)	1470.22382(14)	1480.04616(16)	1479.22341(13)	1455.84690(26)
*D*_J_/10^–3^	0.30236(65)	0.2987(15)	0.3046(20)	0.3004(14)	0.3122(38)
*D*_JK_/10^–3^	0.74791(87)	0.7372(29)	0.7128(31)	0.7407(24)	0.6096(98)
*D*_K_/10^–3^	–0.5568(27)	–0.557(13)	–0.562(12)	–0.546(12)	–0.486(39)
*d*_1_/10^–3^	–0.042384(50)	–0.04142(41)	–0.04090(43)	–0.04266(26)	–0.0454(16)
*d*_2_/10^–3^	0.013047(24)	0.01291(15)	0.01257(18)	0.013242(88)	0.01363(52)
no. of rotational transitions	235	139	138	140	68
no. of *a* type	30	23	22	22	18
no. of *b* type	108	44	48	50	10
no. of *c* type	97	72	68	68	40
*J* range	1–20	1–13	1–13	1–13	1–10
*K*_a_ range	0–9	0–5	0–5	0–5	0–4
rms/kHz	4.81	5.26	5.44	4.83	6.42

a 1σ standard deviations in the parameters are given in parentheses.

**Table 7:** Spectroscopic Constants (in MHz, Unless as Otherwise Noted) for Five Isotopologues of tFTFO^a^^,^^b^

	t-CHFCH(CF_3_)O	t-^13^CHFCH(CF_3_)O	t-CHF^13^CH(CF_3_)O	t-CHFCH(^13^CF_3_)O	t-CHFCH(CF_3_)^18^O
*A*	4100.94130(18)	4096.51948(27)	4091.14264(27)	4101.04520(27)	4013.10127(42)
*B*	1317.036356(56)	1308.53218(13)	1315.59375(13)	1313.64633(12)	1308.10893(44)
*C*	1246.837608(56)	1239.58030(12)	1246.43081(12)	1243.78474(12)	1230.76572(36)
*D*_J_/10^–3^	0.08763(15)	0.08538(99)	0.0878(10)	0.0881(10)	0.0969(58)
*D*_JK_/10^–3^	0.63224(79)	0.6306(29)	0.6164(26)	0.6330(28)	0.557(13)
*D*_K_/10^–3^	0.2361(13)	[0.2361]	[0.2361]	[0.2361]	[0.2361]
*d*_1_/10^–3^	–0.008329(20)	–0.00888(16)	–0.00817(17)	–0.00803(13)	–0.0040(19)
*d*_2_/10^–3^	0.0017933(59)	0.001715(53)	0.001776(44)	0.001732(43)	0.00333(60)
no. of rotational transitions	264	78	92	89	31
no. of *a* type	44	17	26	24	3
no. of *b* type	193	60	66	65	28
no. of *c* type	27	1	0	0	0
*J* range	1–23	1–14	1–14	1–14	1–8
*K*_a_ range	0–9	0–3	0–4	0–3	0–4
rms/kHz	4.77	3.93	4.35	4.11	4.00

a 1σ standard deviations in the parameters are given in parentheses.

b The value of *D*_K_ in square brackets for each minor isotopologue is fixed to that for the most abundant isotopologue.

#### 4.1.2 Ar-cFTFO and Ar-tFTFO

We have assigned 368 *a*-, *b*-, and *c*-type transitions consistent with isomer (a) in Figure 3 for the most abundant species of Ar-cFTFO and 176 *a*- and *b*-type transitions, also consistent with isomer (a) in Figure 4 for the most abundant species of Ar-tFTFO. Once again, the line intensities agree with theoretical predictions of the magnitudes of the dipole moment components: for cFTFO, these are calculated to be 1.89, 0.35, and 2.23 D along the *a*-, *b*-, and *c*-axes (Table 3), and we have observed in the broadband spectrum strong *c*-type transitions, weaker *a* type, and still weaker *b*-type transitions. For Ar-tFTFO, the magnitudes of the dipole moment components along the *a*-, *b*-, and *c*-axes are calculated to be 1.09, 0.34, and 0.18 D (Table 5). These components are all less than their Ar-cFTFO counterparts, and the observed transitions are only *a*- and *b*-type; no *c* type transitions are assigned. For both species, a large range of *J* and *K*_a_ are sampled: *J* from 1 to 19 for Ar-cFTFO and from 2 to 14 for Ar-tFTFO; and *K*_a_ from 0 to at least 8. Figure 5 shows some *a*-type transitions for the most abundant isotopologue of Ar-tFTFO, readily observed among those for the tFTFO monomer. Because the *a* and *c* dipole components for Ar-cFTFO are significantly greater than those for Ar-tFTFO, we are able to observe transitions due to the three naturally occurring ^13^C isotopologues of Ar-cFTFO in the broadband spectrum but have to turn to the Balle-Flygare spectrometer to collect transitions (only *a*-type) for the minor isotopologues of Ar-tFTFO. No evidence of internal rotation is observed in either complex.

The transitions for each species are once again analyzed using the Watson *S*-reduced Hamiltonian^24,25^ in the I^*r*^ representation and Pickett’s nonlinear SPFIT program.^26^ For the isotopologues of Ar-cFTFO and Ar-tFTFO, we determine three rotational constants and five quartic centrifugal distortion constants, as well as four and two sextic centrifugal distortion constants for the most abundant species of each, respectively (Tables 8 and 9). The rms deviation for each fit is 5–7 kHz, whereas the fit for each minor Ar-tFTFO isotopologue is about 1 kHz. Tables of observed and calculated transition frequencies with assignments for all isotopologues studied are provided as Supporting Information.

**Table 8:** Spectroscopic Constants (in MHz, Unless as Otherwise Noted) for Four Isotopologues of Ar-cFTFO^a^^,^^b^

	Ar-c-CHFCH(CF_3_)O	Ar-c-^13^CHFCH(CF_3_)O	Ar-c-CHF^13^CH(CF_3_)O	Ar-c-CHFCH(^13^CF_3_)O
*A*	1638.11628(18)	1624.61612(76)	1633.28478(87)	1635.7948(11)
*B*	798.696848(72)	798.57949(25)	797.16066(27)	796.80292(31)
*C*	642.119434(73)	640.16482(24)	641.65779(23)	640.52760(28)
*D*_J_/10^–3^	1.12799(53)	1.1158(14)	1.1218(15)	1.1243(19)
*D*_JK_/10^–3^	3.6595(19)	3.544(13)	3.642(15)	3.566(22)
*D*_K_/10^–3^	–0.4104(71)	–0.307(42)	–0.620(55)	–0.266(75)
*d*_1_/10^–3^	–0.285138(66)	–0.28467(75)	–0.28134(99)	–0.2847(12)
*d*_2_/10^–3^	–0.008820(26)	–9.94(47)	–9.43(54)	–9.02(69)
no. of rotational transitions	368	54	56	53
no. of *a* type	117	28	36	34
no. of *b* type	49	0	0	0
no. of *c* type	202	26	20	19
*J* range	1–19	2–11	2–11	2–11
*K*_a_ range	0–8	0–4	0–4	0–4
rms/kHz	4.84	5.58	5.37	6.66

a 1σ standard deviations in the parameters are given in parentheses.

b Four sextic centrifugal distortion constants, in Hz, have also been determined for the most abundant isotopologue, Ar-c-CHFCH(CF_3_)O: *H*_J_, −0.0108(12); *H*_JK_, 0.0499(53); *H*_KJ_, −0.869(28); and *H*_K_, 0.596(84). The values of these constants for the ^13^C-containing isotopologues cannot be determined and are fixed to the corresponding values for the most abundant species.

**Table 9:** Spectroscopic Constants (in MHz, Unless as Otherwise Noted) for Four Isotopologues of Ar-tFTFO^a^^,^^b^

	Ar-t-CHFCH(CF_3_)O	Ar-t-^13^CHFCH(CF_3_)O	Ar-t-CHF^13^CH(CF_3_)O	Ar-t-CHFCH(^13^CF_3_)O
*A*	1453.36924(37)	1443.4970(10)	1450.7028(15)	1451.6909(12)
*B*	857.09094(13)	857.06819(11)	855.86403(15)	854.62196(12)
*C*	604.631044(81)	603.062180(60)	603.969518(84)	603.104644(68)
*D*_J_/10^–3^	1.84287(61)	1.84202(68)	1.8196(10)	1.84550(79)
*D*_JK_/10^–3^	–0.9079(52)	–1.033(11)	–0.730(16)	–1.041(13)
*D*_K_/10^–3^	6.266(13)	6.175(80)	6.11(12)	6.105(95)
*d*_1_/10^–3^	–0.70001(28)	–0.70246(40)	–0.69285(56)	–0.70210(46)
*d*_2_/10^–3^	–0.00316(15)	–0.00216(33)	–0.00451(46)	–0.00217(37)
no. of rotational transitions	176	42	42	42
no. of *a* type	122	42	42	42
no. of *b* type	54	0	0	0
*J* range	2–14	3–11	3–11	3–11
*K*_a_ range	0–9	0–3	0–3	0–3
rms/kHz	4.77	0.93	1.31	1.08

a 1σ standard deviations in the parameters are given in parentheses.

b Two sextet centrifugal distortion constants, in Hz, have also been determined for the most abundant isotopologue, Ar-t-CHFCH(CF_3_)O: *H*_JK_, 0.552(32) and *H*_KJ_, −2.57(12). The values of these constants for the ^13^C-containing isotopologues cannot be determined and are fixed to the corresponding values for the most abundant species.

### 4.2 Structure Determination

#### 4.2.1 cFTFO and tFTFO

With asymmetry parameters of −0.831 and −0.951 for the most abundant isotopologues, cFTFO and tFTFO are near prolate asymmetric tops. We use the three moments of inertia of five isotopologues of each monomer to arrive at its average structure using Kisiel’s STRFIT program.^27^ The same eight structural parameters are fitted for each monomer: three bond lengths (C2–C1, C2–C3, and C2–O), four angles (C1C2O, C3C2C1, F1C1C2, and F2C3C2), and one dihedral angle (F1C1C2O). Additionally, we restrict the angles F3C3C2 and F4C3C2 so that the difference between each and F2C3C2 is the same as that determined by theory. All other parameters are fixed to their ab initio values. It should be noted that the two bond lengths and one angle fitted in the three-membered ring (C2–C1, C2–O, and C1C2O) completely determine the ring structure. The fitted parameters (listed above) and the additional structural parameters in the ring derived from them (C1–O, C2OC1, and OC1C2), which are calculated using Kisiel’s EVAL program,^28,29^ are listed in Table 1. The principal coordinates of all atoms in each species are available in Supporting Information, with those of the C and O atoms listed in Table 10. The rms deviation of the fits are 0.0076 and 0.0064 u Å^2^ for cFTFO and tFTFO, respectively.

**Table 10:** Coordinates of Four Atoms in cFTFO and tFTFO Determined from a Structural Fit and from a Kraitchman Analysis^a^

	cFTFO			tFTFO		
	*a*/Å	*b*/Å	*c*/Å	*a*/Å	*b*/Å	*c*/Å
(i) From Structural Fit						
C1	–1.6772(18)	–0.2096(24)	–0.4820(22)	–1.5431(16)	–0.1029(89)	–0.3631(22)
C2	–0.2917(79)	–0.6946(44)	–0.6241(20)	–0.3716(77)	0.0117(28)	0.5450(54)
C3	0.8712(21)	0.0303(14)	–0.0225(13)	1.0031(10)	–0.07636(15)	0.03461(67)
O	–1.2457(15)	–1.2662(14)	0.3015(32)	–1.1446(14)	1.1729(13)	0.0818(27)
(ii) Substitution Coordinates^b^						
C1	–1.67549(90)	–0.1909(79)	–0.4801(31)	–1.54146(97)	–0.079(19)	–0.3586(42)
C2	–0.2657(57)	–0.6929(22)	–0.4798(31)	–0.3597(42)	0.058(26)	0.3571(42)
C3	0.8668(17)	nonphysical	nonphysical	0.9992(15)	–0.028(54)	nonphysical
O	–1.2112(12)	–1.2647(12)	0.3037(49)	–1.1433(13)	1.1720(13)	0.082(18)

a Costain errors^42^ (for the substitution coordinates) or 1σ standard deviations (in the structure fit parameters) are given in parentheses.

b Although only the absolute values of the substitution coordinates can be determined from the Kraitchman analysis, the relative signs are assigned using physically reasonable atomic distances.

Using the moments of inertia of ^13^C- and ^18^O-containing isotopologues, we also carry out a Kraitchman analysis to determine the substitution coordinates^30^ for these atoms in the principal axis system of the most abundant isotopologue of each of cFTFO and tFTFO, and the results are listed in Table 10. A Kraitchman analysis assumes that the structural parameters do not change upon isotopic substitution, which is equivalent to treating the zero-point motions for different isotopologues as the same. This is typically not the case. Therefore, it is not unusual to find that the average and substitution coordinates of an atom do not agree well. For example, while the agreement between the two methods is within 0.05 Å for most atoms, the *c* coordinates for C2 determined by the two methods differ by 0.14 and 0.19 Å for cFTFO and tFTFO, respectively. Another common situation leading to disagreement is when an atomic coordinate is close to zero, giving an ill-determined or a nonphysical imaginary value for that substitution coordinate. This is a consequence of slight differences in the zero-point motion between molecules containing the normal and heavier isotope of the substituted atom, which can also lead to a larger rotational constant for the heavier isotopologue. Thus, the nonphysical or ill-determined values for the substitution *b* and *c* coordinates for C3 (and the larger *A* rotational constant for the ^13^C3-substituted versions) in cFTFO and also in tFTFO indicate that the atom lies close to the *a* axis in each monomer, which is consistent with the average coordinates determined by the structure fit. Despite the fact that Kraitchman analysis does not always provide precise quantitative structural parameters, it can provide good insight into how to arrive at them, as demonstrated in the next section.

#### 4.2.2 Ar-cFTFO and Ar-tFTFO

The asymmetry parameters for Ar-cFTFO and Ar-tFTFO are −0.686 and −0.405; they are each less negative than their respective oxirane monomers and thus even more asymmetric. Only three parameters are required to locate argon in each oxirane. To guide the structure fit of each argon complex, we use the most abundant isotopologue as the parent and calculate the substitution coordinates for the three C atoms using the ^13^C-containing isotopologues. We also use the “extreme substitution” technique to determine the coordinates of Ar in the complex^31,32^ by using the monomeric oxirane as the daughter species. In other words, we substitute Ar in the most abundant isotopologue of the complex with a hypothetical mass of 0 isotope. The substitution coordinates are listed in Table 11, and they show a structure consistent with isomer (a) for each complex, just as expected from the rotational constants that we determined. With these results in hand, we proceeded to determine the average structure of each complex.

**Table 11:** Coordinates of Four Atoms in Ar-cFTFO and Ar-tFTFO Determined from a Structural Fit and from a Kraitchman Analysis^a^

	Ar-cFTFO			Ar-tFTFO		
	*a*/Å	*b*/Å	*c*/Å	*a*/Å	*b*/Å	*c*/Å
(i) From Structural Fit						
C1	–0.01844(26)	–1.57133(11)	–0.37447(8)	–0.04240(46)	–1.51129(20)	0.35979(7)
C2	0.70186(31)	–0.39052(13)	–0.88632(2)	0.67413(6)	–0.57485(13)	–0.54561(1)
C3	1.23429(9)	0.67292(7)	0.02217(1)	1.30013(19)	0.65271(19)	–0.03641(4)
Ar	–2.96421(5)	0.44558(11)	–0.11401(3)	–2.75697(18)	0.79925(25)	0.00265(5)
(ii) Substitution Coordinates^b^						
C1	nonphysical	–1.56231(96)	–0.3593(42)	nonphysical	–1.5074(10)	0.3331(45)
C2	0.6608(23)	–0.3600(42)	–0.3568(42)	0.7491(20)	–0.5967(25)	–0.3320(45)
C3	1.2300(12)	0.6694(22)	nonphysical	1.3076(12)	0.6410(12)	nonphysical
Ar	–2.96432(51)	0.4455(34)	–0.114(13)	–2.75695(54)	0.7990(19)	nonphysical

a Costain errors^42^ (for the substitution coordinates) or 1σ standard deviations (in the structure fit parameters) are given in parentheses.

b Although only the absolute values of the substitution coordinates can be determined from the Kraitchman analysis, the relative signs are assigned using physically reasonable atomic distances.

For each argon complex, we fixed the oxirane structure to its average structure, as determined in the last section. The average position of Ar in Ar-cFTFO is determined by fitting the Ar–C3 distance, the ArC3C2 angle, and the ArC3C2C1 dihedral angle to the three moments of inertia of four isotopologues using Kisiel’s STRFIT program.^27^ The rms deviation for the fit is 0.0277 u Å^2^, and the values for the three fitted parameters are 4.20686(10) Å, 65.600(13)°, and −67.130(14)°. The average coordinates for the C and Ar atoms are listed in Table 11 (and those for all atoms are listed in Supporting Information), and the distance between Ar and the heavy atoms, calculated using Kisiel’s EVAL program,^28,29^ is in Table 2. The substitution and average coordinates of argon agree to a remarkable extent (Table 11); they are identical not only to within experimental uncertainties but indeed within 0.0001 Å.

We attempt to carry out a similar structure fit to locate Ar in Ar-tFTFO, but we are not able to find a set of uncorrelated structural parameters. A potential reason for this difficulty is suggested by the substitution coordinates of Ar in the complex (Table 11). It has a nonphysical *c* coordinate; thus, it lies practically in the *a*–*b* plane. Consequently, the location of the argon atom contributes only to two of the three planar moments for the complex (*P*_*aa*_ and *P*_*bb*_). This might lead to an attempted fit to three structural parameters being ill-conditioned. Regardless of the reason, we could not break the correlation. We therefore use Kisiel’s STRFIT program^27^ to fit only two parameters, the distance Ar–C3 and the dihedral angle ArC3C2C1 to three moments of inertia of four isotopologues while adjusting the ArC3C2 angle to achieve the lowest rms deviation possible. This occurs when the ArC3C2 angle is fixed to 66.90°. The fitted Ar–C3 distance is 4.05993(26) Å and the ArC3C2C1 angle is −70.627(22)°, with an rms deviation of 0.0866 u Å^2^. Once again, the coordinates for the C and Ar atoms can be found in Table 11 (and those for all of the atoms are in Supporting Information). The Ar-heavy atom distances, calculated using Kisiel’s EVAL program^28,29^ are listed in Table 4. As is the case with Ar-cFTFO, the substitution and average coordinates of Ar in Ar-tFTFO are the same, and indeed, the average *c* coordinate shows that it lies in the *a*–*b* plane.

## 5 Discussion

The predicted rotational constants and large dipole moment components for both cFTFO and tFTFO make spectral assignments rather straightforward. Although these predictions are for the equilibrium structures, they approximate the experimental structures well. The experimental *A*, *B*, and *C* rotational constants differ from the theoretical ones (experiment–theory) by 23, −7, and −3 MHz (or 0.6, −0.4, and −0.2%), respectively, for cFTFO and by 35, −7, and −2 MHz (or 0.9, 0.4, and 0.2%), respectively, for tFTFO. Theory also guides us very well for the argon-FTFO heterodimers, being especially remarkable for Ar-tFTFO where the experimental rotational constants differ from the predicted ones without BSSE correction by −0.6, 0.1, and −1.4 MHz (or −0.04, 0.01, and −0.2%). The corresponding differences for Ar-cFTFO are −23, 3, and −2 MHz (or −1.4, 0.3, and −0.3%). The agreement between experimental and predicted constants when BSSE correction is included is not as good; the theoretical values are all too small by 8–51 MHz (or 0.5–6%).

For the structural parameters of the monomers that we can fit, we find the same trends for the average parameters as those for the theoretical values (Table 1). Specifically, the epoxide rings are similar for cFTFO and tFTFO, that is, the bond lengths and angles made by atoms in the rings agree to approximately 2σ. In both molecules, the C1–O bond length is shorter than the C2–O bond length by 0.05–0.06 Å in the theoretical equilibrium structure, as well as the experimental average structure. Thus, the electron density in the C1–O bond is greater than that in the C2–O bond. The average F1C1C2 angle and F2C3C2 angle are greater in cFTFO to minimize F1–F2 repulsion, while the F3C3C2 angle is smaller in cFTFO to facilitate intramolecular interactions between F3 and H2.

The interaction potential contour plots for each of Ar-cFTFO and Ar-tFTFO reveal many isomers, and according to calculations, the energies of the different isomers of the same complex differ by no more than 95 cm^–1^ (∼1 kJ mol^–1^) after applying both BSSE and zero-point corrections. It is, therefore, perhaps unreasonable to expect theory to have the precision to distinguish their energy ordering; yet, in both complexes, we observe the lowest energy isomers predicted by theory [isomer (a) in each case], and they have the same binding mode. Because the carrier gas argon is effective in relaxing a complex to its lowest energy arrangement,^33−35^ we can be quite certain that the observed structures are also lowest in energy experimentally. The interaction lengths between Ar and C1 for Ar-cFTFO and Ar-tFTFO (see Tables 2 and 4) are practically the same, the values being 3.57957(23) and 3.58260(43) Å, respectively, and matching well with the Ar–C van der Waals contact of 3.58 Å. The other interaction lengths shown in Figures 3 and 4 (defined as having distances no greater than 110% of the corresponding van der Waals contact length) are somewhat longer than the van der Waal contact. Additionally, the interaction lengths Ar–C2, Ar–F1, and Ar–F4 are all longer for Ar-cFTFO than for Ar-tFTFO, by 0.102, 0.020, and 0.052 Å, respectively, and thus are somewhat weaker.

**Figure 6:**
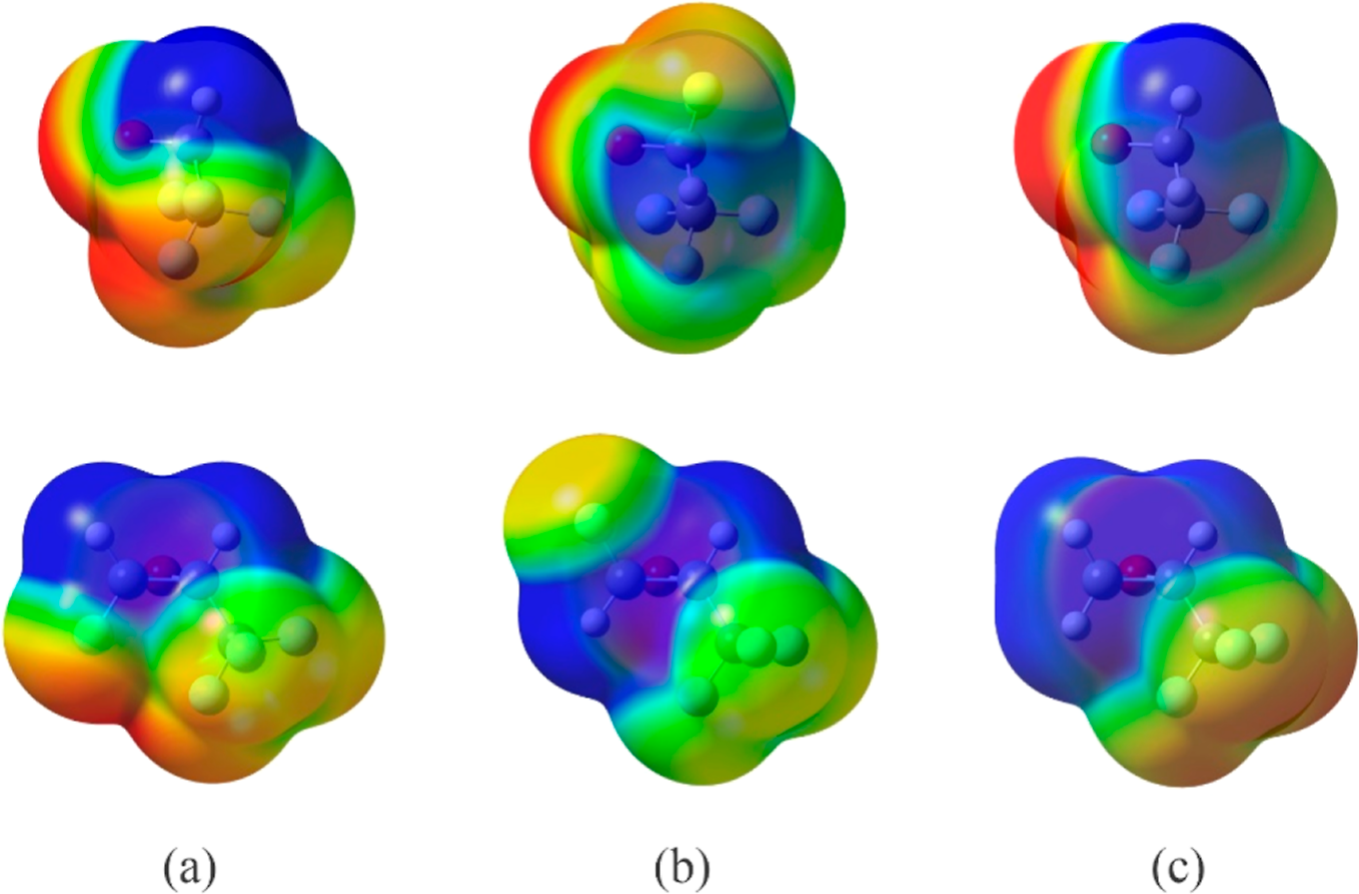
Electrostatic potential mapped onto the total electron density isosurface for (a) cFTFO, (b) tFTFO, and (c) TFO. Two views are shown for each surface. Top shows O pointing to the left and C1–H in front; the bottom shows O pointing to the back and the fluoromethyl group on the right. Same value of electron density is used for the isosurface in all molecules, and identical color scales are used. Blue color represents positive electrostatic potential and red represents negative electrostatic potential.

Argon binds to cFTFO and tFTFO in a different motif than to TFO. Here, we have determined that Ar forms four interactions with each of cFTFO and tFTFO; two of them involve the C atoms in the epoxide ring. The interactions between Ar and TFO include the same C atoms, but Ar is positioned on the opposite side of the epoxide ring from the trifluoromethyl group so that it can also interact with O.^12^ The distances between Ar and O, C1, and C2, respectively, in Ar-TFO, are 3.4744(12), 3.7644(11), and 3.72693(40) Å, which are 0.07, 0.18, and 0.14 Å longer than the corresponding van der Waals contacts. These differences suggest that Ar interacts most strongly with the O atom. The two different sets of binding modes, Ar-TFO versus Ar-FTFO, can be rationalized by examining the mapped electrostatic potentials of these oxiranes onto their respective total electron density surfaces (Figure 6). As can be seen in Figure 6c, the most electronegative atom in TFO is the Ar atom, more so than in cFTFO and tFTFO, and perhaps unexpectedly to an even greater extent than the F atoms in the trifluoromethyl group in TFO; it is, therefore, not surprising that Ar interacts with O instead of the F atoms. In the presence of four F atoms in cFTFO and tFTFO, the combination of the less electronegative O atom and the availability of two F atoms (F1 and F4) located opposite to it makes it energetically more stable for Ar to adopt a different binding modes. Additionally, the location of F1 in tFTFO effectively blocks argon from approaching close enough to interact with both itself and O in a binding mode similar to that in Ar-TFO. In fact, such a motif is not a minimum on the interaction potential surface for Ar and tFTFO. It is interesting to note that when F1 is *cis* to the CF_3_ group, both F1 and the F atoms in the group are more electronegative than those when the groups are *trans* to each other. It appears then that having both groups on the same side of the epoxide ring is more effective in drawing electron density from the rest of the molecule toward themselves.

**Figure 7:**
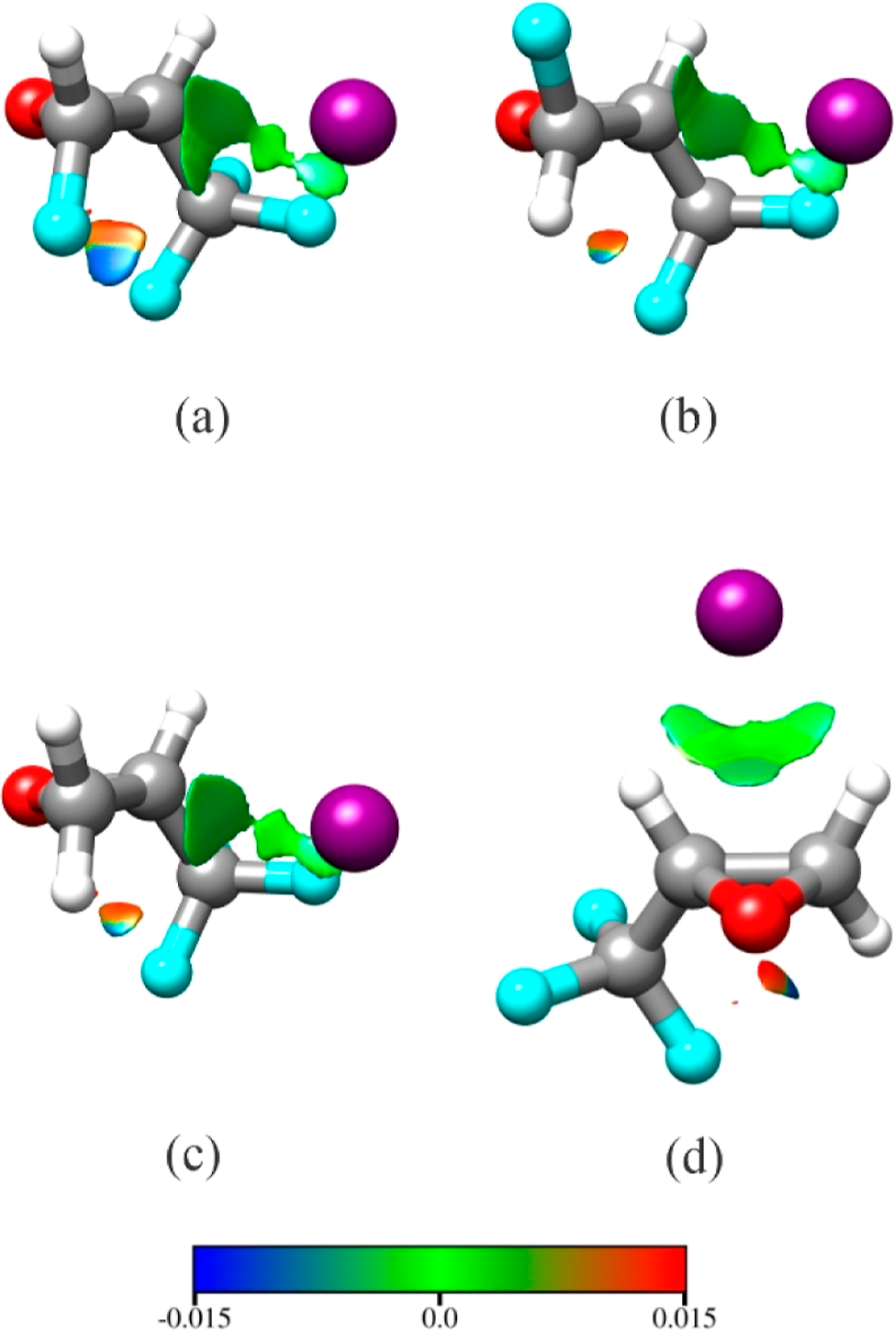
NCI surfaces for the observed back binding mode for (a) Ar-cFTFO and (b) Ar-tFTFO. In (c,d) are, respectively, the theoretically predicted back binding mode and the observed top binding mode for Ar-TFO. Legend displays the color scale in a.u. used for the sign(λ_2_)ρ. Blue and green represent regions of attractive interactions and red represents regions of steric repulsion.

The interactions between Ar and the oxiranes can be viewed with a noncovalent interaction (NCI) analysis^36^ performed using Multiwfn^37^ and visualized using Chimera^38^ (Figure 7). The observed structures of Ar-cFTFO and Ar-tFTFO result from dispersion interactions between Ar and four atoms in the two oxiranes (Figure 7a,b). If Ar-TFO were to assume the same structure, which is determined to be 7.0 cm^–1^ higher in energy than the global minimum structure by theoretical calculation when BSSE correction is taken into account,^12^ then Ar could only interact with three atoms, C1, C2, and one of the F in the fluoromethyl group, and presumably be less stable than the observed structure, which corresponds to the global minimum structure (Figure 7d). As is typically the case, argon is positioned to interact with the greatest number of heavy atoms for both Ar-TFO and the oxirane complexes investigated here.

It will be important to determine whether these observed differences in the electrostatic and steric properties of cFTFO, tFTFO, and TFO that have tuned their intermolecular interactions with the argon atom will translate into advantages or disadvantages in their respective abilities to be effective chiral tagging agents. To the extent that having a wide variety of binding preferences available is an advantage, the movement of the preferred binding site of argon from the “top” or “side” to the “back” of the oxirane ring indicates that having cFTFO and tFTFO in one’s chiral tagging toolkit will provide additional possibilities for interactions with analytes and expand the applicability of the analytical method. Both TFO and cFTFO have large dipole moments (calculated at 2.633 and 2.974 D, respectively) that can enhance the sensitivity of the method, at least in the absence of large opposing dipole moments in the analyte. The dipole moment of tFTFO is somewhat smaller (1.080 D), but that could be advantageous in not canceling out any dipole moment in the analyte. The lowest energy homodimer of each species is heterochiral and possesses a center of symmetry. Hence, these are all nonpolar species and are silent in the microwave spectrum and will not complicate chiral tag-analyte spectra. Similarly, the reported spectra for higher energy homochiral homodimers of TFO^14^ are sufficiently sparse so as not to interfere with the analysis of chiral tag-analyte heterodimer spectra, such as TFO–styrene oxide^5^ or TFO–tFTFO.^39^ Interestingly, transitions appearing in the tFTFO spectrum obtained in this work that we tentatively attributed to a tFTFO homodimer do not appear in the spectrum used to observe TFO–tFTFO despite the clear appearance in that spectrum of both (TFO)_2_ and TFO–tFTFO with intensities typical for 1:1 heterodimers under our expansion conditions. Additionally, transitions assignable to the cFTFO dimer have not been found in the cFTFO spectrum presented here. These and other important questions relevant to the performance of these species as chiral tags are informed by the present results and will form the basis for future investigations.

## 6 Conclusions

With guidance from quantum chemistry calculations, the microwave spectra and molecular structures of *cis*-2-fluoro-3-(trifluoromethyl)oxirane and *trans*-2-fluoro-3-(trifluoromethyl)oxirane and of the gas-phase heterodimer of each with an argon atom have been examined. Apart from the obvious isomerism with respect to the substitution about the ring plane, the two molecules and the two heterodimers appear remarkably similar structurally. However, a closer examination reveals subtle differences, both electronically and sterically. Reminiscent of the *cis* effect for fluorine substitution of a C=C double bond,^40,41^ more electron density is withdrawn from the epoxide ring for the *cis* isomer than for the *trans*. On the steric side, the location of the ring-bound fluorine atom in the *trans* isomer blocks the approach to the epoxide oxygen atom on the face opposite to that already blocked by the trifluoromethyl group. Both electronic and steric differences affect the balance of intramolecular interactions and lead to slight shifts in the dihedral angles between the trifluoromethyl group and the ring plane. Finally, the determination of the zero-point averaged ground vibrational state structures for both *cis*-2-fluoro-3-(trifluoromethyl)oxirane and *trans*-2-fluoro-3-(trifluoromethyl)oxirane is a necessary prerequisite for the rotational spectroscopic characterization of the complexes formed by analytes with these tags, which will be similarly averaged.

Despite the differing amounts of electron withdrawal from the epoxide ring in the two isomers, it is sufficient in both to markedly affect the preferred binding site of an argon atom compared to FO, DFO, and TFO. (As noted above, the preferred site in the latter two of these three is also sterically blocked in the *trans* isomer.) As is typical for argon complexes of fluoroolefins, the argon atom seeks to maximize van der Waals contacts with heavy atoms, and for both *cis*- and *trans*-2-fluoro-3-(trifluoromethyl)oxirane, this is found “in back” of the C–C ring bond, opposite the oxygen atom. The ability to tune electrostatic and steric influences on intermolecular interactions can provide advantages in chiral tagging by offering suitable tags for binding to a wider variety of analytes.

## Supporting Information Available

Observed and calculated transition frequencies for five isotopologues of *cis*- and *trans*-2-fluoro-3-(trifluoromethyl)oxirane and four isotopologues of each of their argon complexes; atomic coordinates for the theoretical and experimentally observed structures of the monomers and the isomers of their argon complexes; (shown in Figures 3 and 4) and complete citation for Gaussian 16 (ref (15))(PDF)

## References

[ref1] Pate B. H.; Evangelisti L.; Caminati W.; Xu Y.; Thomas J.; Patterson D.; Pérez C.; Schnell M., Quantitative Chiral Analysis by Molecular Rotational Spectroscopy. In Chiral Analysis, 2nd ed.; Elsevier: 2018; pp 679–729.

[ref2] Domingos S. R.; Pérez C.; Schnell M. Sensing Chirality with Rotational Spectroscopy. Annu. Rev. Phys. Chem. 2018, 69, 499–519. 10.1146/annurev-physchem-052516-050629. 29490206

[ref3] Borho N.; Xu Y. Lock-and-Key Principle on a Microscopic Scale: The Case of the Propylene Oxide···Ethanol Complex. Angew. Chem., Int. Ed. 2007, 46, 2276–2279. 10.1002/anie.200603809. 17111448

[ref4] Seifert N. A.; Pérez C.; Neill J. L.; Pate B. H.; Vallejo-López M.; Lesarri A.; Cocinero E. J.; Castaño F. Chiral Recognition and Atropisomerism in the Sevoflurane Dimer. Phys. Chem. Chem. Phys. 2015, 17, 18282–18287. 10.1039/C5CP01025J. 25959977

[ref5] Domingos S. R.; Pérez C.; Marshall M. D.; Leung H. O.; Schnell M. Assessing the Performance of Rotational Spectroscopy in Chiral Analysis. Chem. Sci. 2020, 11, 10863–10870. 10.1039/D0SC03752D. 34123188 PMC8162261

[ref6] Mills M. D.; Sonstrom R. E.; Vang Z. P.; Neill J. L.; Scolati H. N.; West C. T.; Pate B. H.; Clark J. R. Enantioselective Synthesis of Enantioisotopomers with Quantitative Chiral Analysis by Chiral Tag Rotational Spectroscopy. Angew. Chem., Int. Ed. 2022, 61, e202207275 10.1002/anie.202207275. PMC9403034 35700045

[ref7] Sonstrom R. E.; Neill J. L.; Mikhonin A. V.; Doetzer R.; Pate B. H. Chiral Analysis of Pantolactone with Molecular Rotational Resonance Spectroscopy. Chirality 2022, 34, 114–125. 10.1002/chir.23379. 34698412

[ref8] Xie F.; Seifert N. A.; Hazrah A. S.; Jäger W.; Xu Y. Conformational Landscape, Chirality Recognition and Chiral Analyses: Rotational Spectroscopy of Tetrahydro-2-Furoic Acid···Propylene Oxide Conformers. ChemPhysChem 2021, 22, 455–460. 10.1002/cphc.202000995. 33453085

[ref9] Brown G. G.; Dian B. C.; Douglass K. O.; Geyer S. M.; Pate B. H. The Rotational Spectrum of Epifluorohydrin Measured by Chirped-Pulse Fourier Transform Microwave Spectroscopy. J. Mol. Spectrosc. 2006, 238, 200–212. 10.1016/j.jms.2006.05.003.

[ref10] Leung H. O.; Marshall M. D.; Stuart D. J. Microwave Spectrum and Molecular Structure of 3-Fluoro-1,2-Epoxypropane and the Unexpected Structure of Its Complex with the Argon Atom. J. Phys. Chem. A 2020, 124, 1798–1810. 10.1021/acs.jpca.0c00327. 32048844

[ref11] Marshall M. D.; Leung H. O. The Microwave Spectrum and Molecular Structure of 3,3-Difluoro-1,2-Epoxypropane and Its Complex with the Argon Atom. J. Mol. Spectrosc. 2018, 350, 18–26. 10.1016/j.jms.2018.05.003.

[ref12] Marshall M. D.; Leung H. O.; Wang K.; Acha M. D. Microwave Spectrum and Molecular Structure of the Chiral Tagging Candidate, 3,3,3-Trifluoro-1,2-Epoxypropane and Its Complex with the Argon Atom. J. Phys. Chem. A 2018, 122, 4670–4680. 10.1021/acs.jpca.8b02550. 29694783

[ref13] Marshall M. D.; Leung H. O. Molecular Structures and Microwave Spectra of the Gas-Phase Homodimers of 3-Fluoro-1,2-Epoxypropane and 3,3-Difluoro-1,2-Epoxypropane. J. Phys. Chem. A 2023, 127, 6267–6274. 10.1021/acs.jpca.3c03643. 37471074

[ref14] Marshall M. D.; Leung H. O.; Domingos S. R.; Krin A.; Schnell M.; Seifert N. A.; Xu Y. J.; Jäger W. Examining the Gas-Phase Homodimers of 3,3,3-Trifluoro-1,2-Epoxypropane Using Quantum Chemistry and Microwave Spectroscopy. Phys. Chem. Chem. Phys. 2022, 24, 28495–28505. 10.1039/D2CP04663F. 36408893

[ref15] Frisch M. J.; Trucks G. W.; Schlegel H. B.; Scuseria G. E.; Robb M. A.; Cheeseman J. R.; Scalmani G.; Barone V.; Petersson G. A.; Nakatsuji H.; Gaussian 16, Revision A.03; Gaussian, Inc.: Wallingford, CT, 2016.

[ref16] Bondi A. Van Der Waals Volumes and Radii. J. Phys. Chem. 1964, 68, 441–451. 10.1021/j100785a001.

[ref17] Boys S. F.; Bernardi F. The Calculation of Small Molecular Interactions by the Differences of Separate Total Energies. Some Procedures with Reduced Errors. Mol. Phys. 1970, 19, 553–566. 10.1080/00268977000101561.

[ref18] Marshall M. D.; Leung H. O.; Scheetz B. Q.; Thaler J. E.; Muenter J. S. A Chirped Pulse Fourier Transform Microwave Study of the Refrigerant Alternative 2,3,3,3-Tetrafluoropropene. J. Mol. Spectrosc. 2011, 266, 37–42. 10.1016/j.jms.2011.02.005.

[ref19] Marshall M. D.; Leung H. O.; Calvert C. E. Molecular Structure of the Argon-(Z)-1-Chloro-2-Fluoroethylene Complex from Chirped-Pulse and Narrow-Band Fourier Transform Microwave Spectroscopy. J. Mol. Spectrosc. 2012, 280, 97–103. 10.1016/j.jms.2012.05.008.

[ref20] Leung H. O.; Marshall M. D.; Messinger J. P.; Knowlton G. S.; Sundheim K. M.; Cheung-Lau J. C. The Microwave Spectra and Molecular Structures of 2-Chloro-1,1-Difluoroethylene and Its Complex with the Argon Atom. J. Mol. Spectrosc. 2014, 305, 25–33. 10.1016/j.jms.2014.09.011.

[ref21] Leung H. O.; Gangwani D.; Grabow J. U. Nuclear Quadrupole Hyperfine Structure in the Microwave Spectrum of Ar-N_2_O. J. Mol. Spectrosc. 1997, 184, 106–112. 10.1006/jmsp.1997.7293.

[ref22] Hartwig H.; Dreizler H. The Microwave Spectrum of Trans-2,3-Dimethyloxirane in Torsional Excited States. Z. Naturforsch. 1996, 51, 923–932. 10.1515/zna-1996-0807.

[ref23] Kisiel Z.; Pszczółkowski L.; Medvedev I. R.; Winnewisser M.; deLucia F. C.; Herbst E. Rotational Spectrum of Trans-Trans Diethyl Ether in the Ground and Three Excited Vibrational States. J. Mol. Spectrosc. 2005, 233, 231–243. 10.1016/j.jms.2005.07.006.

[ref24] Watson J. K. G. Aspects of Quartic and Sextic Centrifugal Effects on Rotational Energy Levels. In Vibrational Spectra and Structure; Durig J. R., Ed.; Elsevier Scientific Publishing: Amsterdam, 1977; Vol. 6, pp 1–89.

[ref25] van Eijck B. P. Reformulation of Quartic Centrifugal Distortion Hamiltonian. J. Mol. Spectrosc. 1974, 53, 246–249. 10.1016/0022-2852(74)90129-5.

[ref26] Pickett H. M. The Fitting and Prediction of Vibration-Rotation Spectra with Spin Interactions. J. Mol. Spectrosc. 1991, 148, 371–377. 10.1016/0022-2852(91)90393-O.

[ref27] Kisiel Z. Least-Squares Mass-Dependence Molecular Structures for Selected Weakly Bound Intermolecular Clusters. J. Mol. Spectrosc. 2003, 218, 58–67. 10.1016/S0022-2852(02)00036-X.

[ref28] Kisiel Z. Assignment and Analysis of Complex Rotational Spectra. In Spectroscopy from Space; Demaison J., Sarka K., Cohen E. A., Eds.; Kluwer Academic Publishers: Dordrecht, 2001.

[ref29] Kisiel Z. Prospe-Programs for Rotational Spectroscopy. http://info.ifpan.edu.pl/~kisiel/prospe.htm (accessed May 21, 2024).

[ref30] Kraitchman J. Determination of Molecular Structure from Microwave Spectroscopic Data. Am. J. Phys. 1953, 21, 17–24. 10.1119/1.1933338.

[ref31] Munrow M. R.; Pringle W. C.; Novick S. E. Determination of the Structure of the Argon Cyclobutanone van der Waals Complex. J. Phys. Chem. A 1999, 103, 2256–2261. 10.1021/jp9836527.

[ref32] Jochims E.; Grabow J. U.; Stahl W. Microwave Spectrum and Structure of the 1,2-Difluorobenzene-Argon van der Waals Complex. J. Mol. Spectrosc. 1993, 158, 278–286. 10.1006/jmsp.1993.1072.

[ref33] Klots T. D.; Ruoff R. S.; Gutowsky H. S. Rotational Spectrum and Structure of the Linear CO_2_-HCN Dimer: Dependence of Isomer Formation on Carrier Gas. J. Chem. Phys. 1989, 90, 4216–4221. 10.1063/1.455778.

[ref34] Emilsson T.; Germann T. C.; Gutowsky H. S. Kinetics of Molecular Association and Relaxation in a Pulsed Supersonic Expansion. J. Chem. Phys. 1992, 96, 8830–8839. 10.1063/1.462240.

[ref35] Ruoff R. S.; Klots T. D.; Emilsson T.; Gutowsky H. S. Relaxation of Conformers and Isomers in Seeded Supersonic Jets of Inert Gases. J. Chem. Phys. 1990, 93, 3142–3150. 10.1063/1.458848.

[ref36] Johnson E. R.; Keinan S.; Mori-Sánchez P.; Contreras-Garcia J.; Cohen A. J.; Yang W. T. Revealing Noncovalent Interactions. J. Am. Chem. Soc. 2010, 132, 6498–6506. 10.1021/ja100936w. 20394428 PMC2864795

[ref37] Lu T.; Chen F. W. Multiwfn: A Multifunctional Wavefunction Analyzer. J. Comput. Chem. 2012, 33, 580–592. 10.1002/jcc.22885. 22162017

[ref38] Pettersen E. F.; Goddard T. D.; Huang C. C.; Couch G. S.; Greenblatt D. M.; Meng E. C.; Ferrin T. E. UCSF Chimera-a Visualization System for Exploratory Research and Analysis. J. Comput. Chem. 2004, 25, 1605–1612. 10.1002/jcc.20084. 15264254

[ref39] Aucoin J. M.; Leung H. O.; Marshall M. D. The Microwave Spectrum and Molecular Structure of Trans-2-fluoro-3-(trifluoromethyl)oxirane and Its Complex with the Argon Atom. In The 2021 International Symposium on Molecular Spectroscopy, Talk RK-02: Urbana-Champaign, IL, 2021.

[ref40] Craig N. C.; Entemann E. A. Thermodynamics of Cis-Trans Isomerizations. The 1,2,-Difluoroethylenes. J. Am. Chem. Soc. 1961, 83, 3047–3050. 10.1021/ja01475a019.

[ref41] Uchimaru T.; Mizukado J. Computational Studies of Fluorinated Propenes: The Fluorine "Cis Effect," Barrier Hights of the Internal Rotation of CX_3_ (X = H or F) Group, and Pi-Bond Strengths. J. Fluorine Chem. 2021, 245, 109772 10.1016/j.jfluchem.2021.109772.

[ref42] Costain C. C. Determination of Molecular Structures from Ground State Rotational Constants. J. Chem. Phys. 1958, 29, 864–874. 10.1063/1.1744602.

