# Supplementary material for: Tuning Intermolecular Interactions for Chiral Analysis: The Microwave Spectra and Molecular Structures of the Chiral Tag Candidates cis- and trans-2-Fluoro-3-(trifluoromethyl)oxirane and Their Gas-Phase Heterodimers with the Argon Atom
Source: J Phys Chem A. 2024 Oct 16;128(43):9433–46. doi: 10.1021/acs.jpca.4c05830 (PMC11533193; doi:10.1021/acs.jpca.4c05830)
Supplement: Supplementary file 1 — jp4c05830_si_001.pdf [file jp4c05830_si_001.pdf]

Tuning Intermolecular Interactions for Chiral Analysis: The Microwave Spectra and Molecular Structures of the Chiral Tag Candidates *cis*- and *trans*-2-Fluoro-3-(trifluoromethyl)oxirane and Their Gas-phase Heterodimers with the Argon Atom

Helen O. Leung,* Mark D. Marshall,* Jordan M. Aucoin, and Jonah R. Horowitz

Department of Chemistry, Amherst College, P.O. Box 5000, Amherst, MA 01002-5000, United States

Supporting Information

Address for correspondence: Prof. Mark D. Marshall
Department of Chemistry
Amherst College
P.O. Box 5000
Amherst, MA 01002-5000
Telephone: (413) 542-2006
Fax: (413) 542-2735
E-mail: mdmarshall@amherst.edu

*Corresponding authors. Fax: +1-413-542-2735; *e-mail addresses:* hleung@amherst.edu (H.O. Leung), mdmarshall@amherst.edu (M.D. Marshall).

Tables S1 and S2 presents the atomic coordinates in the appropriate principal inertial axis system for the structures of *cis*- and *trans*-2-fluoro-3-(trifluoromethyl)oxirane and their argon complexes obtained via quantum chemistry calculations. Table S21 contains similar data for the experimentally determined structures along with uncertainties from the structural fits.

Tables S3 through S20 contain the quantum number assignments, observed transition frequencies (in MHz), and the residuals (obs. - calc., also in MHz) from the least squares fits. The quantum numbers are J, K_a, and K_c, the usual asymmetric top rotational quantum numbers for the initial and final levels of the transition.

The complete reference for Gaussian 16 (reference 15) is given here.

Frisch, M. J.; Trucks, G. W.; Schlegel, H. B.; Scuseria, G. E.; Robb, M. A.; Cheeseman, J. R.; Scalmani, G.; Barone, V.; Petersson, G. A.; Nakatsuji, H.; Li, X.; Caricato, M.; Marenich, A. V.; Bloino, J.; Janesko, B. G.; Gomperts, R.; Mennucci, B.; Hratchian, H. P.; Ortiz, J. V.; Izmaylov, A. F.; Sonnenberg, J. L.; Williams-Young, F.; Ding, F.; Lipparini, F.; Egidi, F.; Goings, J.; Peng, B.; Petrone, A.; Henderson, T.; Ranasinghe, D.; Zakrzewski, V. G.; Gao, J.; Rega, N.; Zheng, G.; Liang, W.; Hada, M.; Ehara, M.; Toyota, K.; Fukuda, R.; Hasegawa, J.; Ishida, M.; Nakajima, T.; Honda, Y.; Kitao, O.; Nakai, H.; Vreven, T.; Throssell, K.; Montgomery Jr., J. A.; Peralta, J. E.; Ogliaro, F.; Bearpark, M. J.; Heyd, J. J.; Brothers, E. N.; Kudin, K. N.; Staroverov, V. N.; Keith, T. A.; Kobayashi, R.; Normand, J.; Raghavachari, K.; Rendell, A. P.; Burant, J. C.; Iyengar, S. S.; Tomasi, J.; Cossi, M.; Millam, J. M.; Klene, M.; Adamo, C.; Cammi, R.; Ochterski, J. W.; Martin, R. L.; Morokuma, K.; Farkas, O.; Foresman, J. B.; Fox, D. J. *Gaussian 16*, Revision A.03; Gaussian, Inc.: Wallingford, CT, 2016.

Table S1: Principal coordinates for the theoretical structures of cFTFO and its argon complex

cFTFO

	a	*b*	*c*
C1	-1.6676	-0.2022	-0.4850
C2	-0.3004	-0.6856	-0.6318
C3	0.8708	0.0334	-0.0208
O	-1.2412	-1.2800	0.2988
H1	-2.4360	-0.4079	-1.2149
H2	-0.0245	-1.2979	-1.4790
F1	-1.9148	0.9530	0.1704
F2	0.5894	0.6239	1.1348
F3	1.8779	-0.8342	0.1893
F4	1.3160	0.9652	-0.8845

Ar-cFTFO
No BSSE Correction

	Structure (a)			Structure (b)			Structure (c)		
	a	*b*	*c*	*a*	*b*	*c*	*a*	*b*	*c*
C1	0.0581	1.5418	-0.3677	1.1642	1.6034	-0.4976	-0.2272	1.2708	-0.1857
C2	-0.6893	0.4012	-0.8827	0.4066	0.3709	-0.6758	0.1506	-0.1349	-0.1080
C3	-1.2544	-0.6579	0.0233	0.8181	-0.9207	-0.0244	1.5879	-0.5731	-0.0452
O	-1.2725	1.7239	-0.7603	-0.0250	1.4283	0.2187	-0.3426	0.6038	1.0389
H1	0.8096	2.0524	-0.9508	1.1730	2.3896	-1.2373	-1.1463	1.5986	-0.6475
H2	-0.5250	0.0498	-1.8917	-0.1994	0.2239	-1.5589	-0.5075	-0.9005	-0.4943
F1	0.3114	1.6490	0.9549	2.3043	1.6031	0.2269	0.7230	2.2284	-0.2579
F2	-1.6511	-0.1941	1.2025	1.3856	-0.7601	1.1653	2.3743	0.2720	0.6111
F3	-2.3096	-1.2472	-0.5687	-0.2574	-1.7146	0.1310	1.6716	-1.7733	0.5578
F4	-0.3232	-1.6091	0.2247	1.6815	-1.5661	-0.8309	2.0602	-0.7181	-1.2975
Ar	2.9568	-0.4627	-0.1176	-3.1631	0.2050	0.0135	-3.5216	-0.4325	-0.1015

	Structure (d)			Structure (e)			Structure (f)		
	a	*b*	*c*	*a*	*b*	*c*	*a*	*b*	*c*
C1	1.5158	1.5006	-0.4839	-0.3264	-1.8137	-0.2375	-0.3902	0.4563	-0.8244
C2	1.0041	0.2228	-0.9637	-1.2276	-0.7749	-0.7207	0.8371	-0.3268	-0.8969
C3	0.7530	-0.9353	-0.0376	-1.4437	0.5086	0.0329	1.9682	-0.1304	0.0746
O	0.1533	1.3834	-0.7803	0.0984	-0.9752	-1.2740	-0.3946	-0.8579	-0.3442
H1	2.0924	2.1650	-1.1096	-0.4610	-2.8565	-0.4822	-1.0007	0.6574	-1.6917
H2	1.1696	-0.0834	-1.9872	-2.0531	-1.0261	-1.3719	1.1568	-0.7574	-1.8355
F1	1.8326	1.6611	0.8194	0.3071	-1.6688	0.9468	-0.5220	1.4316	0.1010
F2	0.3487	-0.5709	1.1736	-0.3642	0.9362	0.6773	1.5692	0.2037	1.2963
F3	-0.1854	-1.7436	-0.5641	-1.8310	1.4763	-0.8184	2.6899	-1.2625	0.1674
F4	1.8833	-1.6565	0.0836	-2.4378	0.3351	0.9240	2.7871	0.8317	-0.3902
Ar	-2.9706	0.2553	0.1174	2.9807	0.6000	0.0122	-3.6729	-0.2264	0.1628

Table S1: Principal coordinates for the theoretical structures of cFTFO and its argon complex

	Structure (g)			Structure (h)			Structure (i)		
	a	*b*	*c*	*a*	*b*	*c*	*a*	*b*	*c*
C1	-1.8128	-1.3753	-0.1142	-1.0019	1.7090	-0.4793	-2.7686	0.2351	-0.2273
C2	-0.6150	-0.6759	0.3340	-1.6133	0.3917	-0.6034	-1.7152	-0.7649	-0.3500
C3	-0.3881	0.7830	0.0478	-0.9764	-0.8419	-0.0251	-0.2620	-0.4195	-0.1749
O	-1.7150	-0.9880	1.2268	-2.0772	1.3790	0.3528	-2.5461	-0.6499	0.8335
H1	-1.8235	-2.4365	-0.3126	-1.1700	2.4954	-1.1996	-3.7134	0.1468	-0.7420
H2	0.3029	-1.2195	0.5088	-2.2859	0.1754	-1.4216	-1.8691	-1.6510	-0.9498
F1	-2.7397	-0.7314	-0.8566	0.1994	1.8482	0.1227	-2.4556	1.5297	-0.0017
F2	-1.5039	1.5027	0.0326	-0.3111	-0.6194	1.1023	-0.0460	0.5381	0.7194
F3	0.4325	1.3066	0.9771	-1.9212	-1.7672	0.2247	0.4219	-1.5122	0.2118
F4	0.2229	0.9088	-1.1452	-0.1289	-1.3666	-0.9300	0.2440	-0.0263	-1.3589
Ar	3.2762	-0.5514	-0.1047	3.0248	-0.0917	0.0105	3.4576	0.3315	0.1391

With BSSE Correction

	Structure (a)			Structure (b)			Structure (c)		
	a	*b*	*c*	*a*	*b*	*c*	*a*	*b*	*c*
C1	-0.0103	1.5492	-0.3532	1.1319	1.6227	-0.4815	-0.1842	1.2749	-0.1791
C2	-0.6734	0.3615	-0.8769	0.3922	0.3744	-0.6204	0.1833	-0.1332	-0.0955
C3	-1.2675	-0.6847	0.0255	0.8814	-0.9148	-0.0200	1.6177	-0.5826	-0.0466
O	-1.3124	1.6636	-0.8524	0.0041	1.4096	0.3189	-0.2902	0.6179	1.0517
H1	0.7647	2.0671	-0.8977	1.0669	2.4181	-1.2085	-1.1061	1.6067	-0.6323
H2	-0.4190	-0.0205	-1.8557	-0.2745	0.2237	-1.4579	-0.4856	-0.8963	-0.4681
F1	0.1360	1.7156	0.9794	2.3228	1.6412	0.1560	0.7727	2.2241	-0.2698
F2	-1.7708	-0.1933	1.1517	1.5325	-0.7553	1.1263	2.4186	0.2609	0.5939
F3	-2.2516	-1.3370	-0.6205	-0.1605	-1.7367	0.2038	1.6989	-1.7790	0.5642
F4	-0.3204	-1.5901	0.3349	1.6969	-1.5288	-0.8977	2.0737	-0.7407	-1.3033
Ar	3.1024	-0.4177	-0.1050	-3.3072	0.1755	-0.0033	-3.6399	-0.4166	-0.0995

	Structure (d)			Structure (e)			Structure (f)		
	a	*b*	*c*	*a*	*b*	*c*	*a*	*b*	*c*
C1	1.4952	1.5385	-0.4923	-0.3789	-1.8069	-0.2231	-0.3590	0.4575	-0.7919
C2	1.0132	0.2458	-0.9626	-1.2881	-0.7751	-0.7063	0.8563	-0.3431	-0.8744
C3	0.8342	-0.9251	-0.0358	-1.4938	0.5181	0.0333	2.0119	-0.1396	0.0664
O	0.1285	1.3745	-0.7439	0.0295	-0.9820	-1.2770	-0.3689	-0.8454	-0.2818
H1	2.0267	2.2261	-1.1327	-0.5164	-2.8530	-0.4518	-0.9868	0.6468	-1.6495
H2	1.1552	-0.0493	-1.9929	-2.1232	-1.0354	-1.3415	1.1489	-0.7988	-1.8099
F1	1.8495	1.7038	0.8008	0.2724	-1.6458	0.9494	-0.4572	1.4550	0.1137
F2	0.4580	-0.5812	1.1903	-0.4050	0.9549	0.6555	1.6453	0.2273	1.2889
F3	-0.0921	-1.7636	-0.5359	-1.8947	1.4742	-0.8248	2.7213	-1.2786	0.1687
F4	1.9927	-1.6063	0.0433	-2.4741	0.3560	0.9416	2.8318	0.8011	-0.4388
Ar	-3.1360	0.2053	0.1118	3.1439	0.5692	0.0069	-3.8147	-0.2230	0.1420

Table S1: Principal coordinates for the theoretical structures of cFTFO and its argon complex

	Structure (g)			Structure (h)			Structure (i)		
	a	*b*	*c*	*a*	*b*	*c*	*a*	*b*	*c*
C1	-1.7843	-1.4207	-0.0979	-1.0579	1.7070	-0.4786	-2.8180	0.2717	-0.2195
C2	-0.6245	-0.6593	0.3490	-1.6674	0.3886	-0.6005	-1.7888	-0.7544	-0.3322
C3	-0.4623	0.8036	0.0400	-1.0257	-0.8440	-0.0253	-0.3268	-0.4397	-0.1729
O	-1.7175	-1.0071	1.2372	-2.1291	1.3747	0.3581	-2.6084	-0.6001	0.8547
H1	-1.7441	-2.4843	-0.2782	-1.2306	2.4933	-1.1979	-3.7683	0.1965	-0.7263
H2	0.3158	-1.1569	0.5412	-2.3431	0.1713	-1.4159	-1.9673	-1.6467	-0.9157
F1	-2.7327	-0.8328	-0.8593	0.1457	1.8483	0.1183	-2.4738	1.5624	-0.0183
F2	-1.6098	1.4706	0.0027	-0.3559	-0.6206	1.0993	-0.0826	0.5277	0.7034
F3	0.3243	1.3800	0.9676	-1.9676	-1.7711	0.2283	0.3345	-1.5412	0.2279
F4	0.1538	0.9377	-1.1494	-0.1811	-1.3668	-0.9340	0.1795	-0.0785	-1.3668
Ar	3.4227	-0.5269	-0.0955	3.1901	-0.0851	0.0109	3.6411	0.3301	0.1327

Table S2: Principal coordinates for the theoretical structures of tFTFO and its argon complex

tFTFO

	a	*b*	*c*
C1	1.5403	0.0765	-0.3595
C2	0.4010	-0.0242	0.5412
C3	-1.0050	0.0783	0.0321
O	1.1393	-1.1866	0.0860
H1	1.4596	0.2739	-1.4169
H2	0.5310	0.2323	1.5824
F1	2.7111	0.5267	0.1495
F2	-1.0754	-0.1371	-1.2878
F3	-1.8064	-0.8082	0.6382
F4	-1.4854	1.3083	0.2839

Ar-tFTFO
No BSSE Correction

	Structure (a)			Structure (b)			Structure (c)		
	a	*b*	*c*	*a*	*b*	*c*	*a*	*b*	*c*
C1	-0.0750	-1.4885	0.3193	-0.0256	-1.4894	0.2384	-1.1953	-1.5723	0.2101
C2	0.7588	-0.6560	-0.5357	-1.1281	-0.7533	-0.3634	-0.5111	-0.4051	-0.3273
C3	1.3048	0.6538	-0.0530	-1.4279	0.6608	0.0328	-1.0109	0.9822	-0.0586
O	1.2704	-1.8279	0.1485	0.1394	-0.9493	-1.0406	-0.0286	-1.1238	0.8363
H1	-0.3901	-1.2102	1.3127	0.6051	-1.1000	1.0221	-2.1090	-1.5278	0.7817
H2	0.6652	-0.7460	-1.6080	-1.9644	-1.3018	-0.7715	0.0852	-0.5120	-1.2216
F1	-0.9630	-2.3179	-0.2775	-0.1199	-2.8388	0.2907	-1.0794	-2.7400	-0.4650
F2	1.2829	0.7423	1.2831	-0.3841	1.2422	0.6378	-1.8373	1.0203	0.9945
F3	2.5698	0.8309	-0.4576	-1.7538	1.4046	-1.0330	0.0027	1.8251	0.1816
F4	0.5669	1.6599	-0.5535	-2.4698	0.6781	0.8821	-1.6782	1.4354	-1.1340
Ar	-2.7558	0.7934	0.0315	3.0012	0.6845	0.0682	3.0616	0.0676	-0.0698

	Structure (d)			Structure (e)		
	a	*b*	*c*	*a*	*b*	*c*
C1	-2.1498	-0.7696	-0.1478	2.5574	-0.3207	0.4743
C2	-0.7750	-0.3798	0.1304	1.6112	0.1990	-0.5025
C3	-0.3060	1.0273	-0.0862	0.1321	0.1089	-0.2774
O	-1.7644	-0.5466	1.1775	2.3255	1.0571	0.4232
H1	-2.8941	-0.1077	-0.5618	2.2720	-0.8345	1.3787
H2	0.0003	-1.1312	0.0975	1.9134	0.2751	-1.5367
F1	-2.3873	-2.0619	-0.4735	3.7709	-0.7218	0.0281
F2	-1.3281	1.8902	-0.1511	-0.1653	-0.1021	1.0112
F3	0.5075	1.4250	0.9014	-0.4870	1.2322	-0.6652
F4	0.3799	1.0997	-1.2401	-0.3707	-0.9089	-0.9975
Ar	3.0938	-0.8319	0.0295	-3.6341	-0.1671	0.2228

Table S2: Principal coordinates for the theoretical structures of tFTFO and its argon complex

With BSSE Correction

	Structure (a)			Structure (b)			Structure (c)		
	a	*b*	*c*	*a*	*b*	*c*	*a*	*b*	*c*
C1	0.0139	-1.4971	0.3173	-0.0552	-1.4703	0.2341	-1.2093	-1.5838	0.2073
C2	0.8253	-0.6394	-0.5344	-1.1744	-0.7510	-0.3570	-0.5402	-0.4053	-0.3246
C3	1.3193	0.6916	-0.0536	-1.4865	0.6614	0.0360	-1.0652	0.9735	-0.0601
O	1.3722	-1.7888	0.1606	0.0865	-0.9349	-1.0496	-0.0553	-1.1159	0.8429
H1	-0.3202	-1.2255	1.3063	0.5807	-1.0692	1.0076	-2.1282	-1.5545	0.7715
H2	0.7449	-0.7379	-1.6070	-2.0090	-1.3118	-0.7514	0.0650	-0.5022	-1.2141
F1	-0.8382	-2.3608	-0.2832	-0.1322	-2.8205	0.2945	-1.0685	-2.7493	-0.4668
F2	1.2818	0.7858	1.2818	-0.4422	1.2586	0.6245	-1.9006	0.9978	0.9862
F3	2.5809	0.9120	-0.4474	-1.8353	1.3956	-1.0292	-0.0677	1.8330	0.1882
F4	0.5505	1.6680	-0.5660	-2.5174	0.6703	0.8987	-1.7312	1.4156	-1.1409
Ar	-2.9076	0.7216	0.0316	3.1594	0.6631	0.0649	3.1861	0.0918	-0.0670

	Structure (d)			Structure (e)		
	a	*b*	*c*	*a*	*b*	*c*
C1	-2.1306	-0.8688	-0.1460	2.6066	-0.3516	0.4733
C2	-0.7828	-0.3912	0.1272	1.6743	0.2193	-0.4882
C3	-0.4080	1.0445	-0.0849	0.1922	0.1272	-0.2841
O	-1.7538	-0.6270	1.1784	2.3825	1.0284	0.4850
H1	-2.9187	-0.2552	-0.5534	2.3082	-0.9061	1.3491
H2	0.0397	-1.0901	0.0871	1.9886	0.3426	-1.5142
F1	-2.2848	-2.1725	-0.4767	3.8229	-0.7382	0.0220
F2	-1.4847	1.8390	-0.1409	-0.1209	-0.1429	0.9896
F3	0.3829	1.4902	0.9006	-0.4165	1.2712	-0.6249
F4	0.2657	1.1667	-1.2418	-0.3077	-0.8523	-1.0572
Ar	3.2557	-0.7550	0.0271	-3.8208	-0.1761	0.2186

Table S3: Observed Transition Frequencies (in MHz) for c-CHFCH(CHF_3)O

J'	K_a'	K_c'	J''	K_a''	K_c''	Observed	Obs - Calc
4	1	4	3	2	1	5643.6166	-0.0051
2	2	1	2	1	1	5692.8504	0.0002
12	3	10	12	2	10	5695.7946	-0.0041
4	1	4	3	2	2	5700.9506	0.0012
2	2	0	2	1	1	5704.3836	0.0017
5	2	4	4	3	1	5706.7160	0.0045
5	2	4	4	3	2	5710.0401	-0.0001
10	2	8	10	1	9	6017.5749	-0.0045
8	1	7	8	1	8	6083.7232	-0.0097
5	2	3	4	3	1	6093.1728	0.0020
5	2	3	4	3	2	6096.5025	0.0030
2	1	2	1	1	1	6104.6109	-0.0005
2	2	1	2	1	2	6217.3221	-0.0013
2	2	0	2	1	2	6228.8550	-0.0001
2	0	2	1	0	1	6267.9070	-0.0004
8	1	7	8	0	8	6451.1892	-0.0017
2	1	1	1	1	0	6454.2595	0.0001
3	2	2	3	1	3	6486.5259	0.0002
11	3	9	11	2	9	6499.5654	-0.0018
3	2	1	3	1	3	6543.8523	-0.0010
12	2	10	12	2	11	6552.1249	-0.0045
18	4	15	18	3	15	6671.2595	-0.0003
11	2	9	11	1	10	6772.1917	-0.0030
4	2	3	4	1	4	6848.8623	-0.0020
4	2	2	4	1	4	7018.5843	-0.0010
3	0	3	2	1	1	7114.5524	0.0008
13	8	5	12	9	3	7223.9068	-0.0037
10	3	8	10	2	8	7255.0624	0.0001
5	2	4	5	1	5	7305.9591	0.0024
8	4	5	7	5	3	7380.2763	-0.0018
8	4	4	7	5	2	7388.2107	0.0029
4	1	3	3	2	1	7388.5144	0.0013
4	1	3	3	2	2	7445.8424	0.0016
9	1	8	9	1	9	7459.8780	-0.0017
12	3	9	12	2	10	7540.5599	0.0007
13	3	10	13	2	11	7566.3613	0.0045
3	0	3	2	1	2	7639.0265	0.0017
11	3	8	11	2	9	7691.9290	-0.0008
5	2	3	5	1	5	7692.4155	-0.0005
9	1	8	9	0	9	7706.5637	0.0002
12	2	10	12	1	11	7738.9936	0.0004
17	4	14	17	3	14	7748.8636	0.0002
14	3	11	14	2	12	7802.3447	-0.0009

Table S3: Observed Transition Frequencies (in MHz) for c-CHFCH(CHF_3)O

J'	K_a'	K_c'	J''	K_a''	K_c''	Observed	Obs - Calc
6	2	5	6	1	6	7858.3932	-0.0002
9	3	7	9	2	7	7938.6802	-0.0025
10	3	7	10	2	8	7977.2147	-0.0070
2	1	2	1	0	1	8002.2296	0.0009
13	2	11	13	2	12	8045.4389	-0.0048
12	7	5	11	8	3	8054.6145	-0.0042
12	7	6	11	8	4		
7	3	5	6	4	2	8190.8835	-0.0009
7	3	5	6	4	3	8191.6144	-0.0022
5	1	5	4	2	2	8197.1419	0.0002
15	3	12	15	2	13	8271.4055	-0.0059
7	3	4	6	4	2	8288.0850	-0.0033
7	3	4	6	4	3	8288.8234	0.0030
9	3	6	9	2	7	8344.7523	-0.0053
5	1	5	4	2	3	8366.8606	-0.0020
7	2	6	7	1	7	8505.1057	0.0024
2	1	1	1	0	1	8526.7040	0.0022
8	3	6	8	2	6	8530.2182	-0.0037
6	2	4	6	1	6	8602.1526	-0.0015
8	3	5	8	2	6	8739.6149	-0.0050
6	2	5	5	3	2	8749.8303	-0.0047
6	2	5	5	3	3	8763.0821	-0.0037
16	4	13	16	3	13	8804.4750	-0.0027
10	1	9	10	1	10	8890.7181	0.0023
13	2	11	13	1	12	8899.9730	-0.0068
16	3	13	16	2	14	8985.5075	-0.0107
7	3	5	7	2	5	9014.0723	0.0008
10	1	9	10	0	10	9052.3971	-0.0042
7	3	4	7	2	5	9111.2776	0.0022
3	1	3	2	1	2	9149.9219	0.0039
8	2	7	8	1	8	9242.8980	-0.0015
3	0	3	2	0	2	9373.3497	0.0036
6	3	4	6	2	4	9382.0346	-0.0015
3	2	2	2	2	1	9419.1226	0.0023
6	3	3	6	2	4	9421.4560	0.0019
3	2	1	2	2	0	9464.9180	0.0018
6	2	4	5	3	2	9493.5974	0.0016
6	2	4	5	3	3	9506.8480	0.0015
14	2	12	14	2	13	9621.7932	0.0049
5	3	3	5	2	3	9636.7613	-0.0025
5	3	2	5	2	3	9650.0118	-0.0028
3	1	2	2	1	1	9673.9089	0.0006
10	5	6	9	6	4	9726.8828	0.0052

Table S3: Observed Transition Frequencies (in MHz) for c-CHFCH(CHF_3)O

J'	K_a'	K_c'	J''	K_a''	K_c''	Observed	Obs - Calc
7	2	5	7	1	7	9774.1419	0.0036
4	3	2	4	2	2	9793.0562	-0.0020
4	3	1	4	2	2	9796.3887	0.0019
15	4	12	15	3	12	9805.3367	-0.0036
3	3	1	3	2	1	9874.7980	-0.0046
3	3	0	3	2	1	9875.2796	0.0004
4	0	4	3	1	2	9887.0601	0.0014
3	3	1	3	2	2	9932.1404	0.0102
3	3	0	3	2	2	9932.6155	0.0087
4	3	2	4	2	3	9962.7788	-0.0003
4	3	1	4	2	3	9966.1070	-0.0008
5	3	3	5	2	4	10023.2267	0.0035
5	3	2	5	2	4	10036.4713	-0.0026
17	4	13	17	3	14	10051.4620	0.0038
19	4	15	19	3	16	10058.2467	0.0001
9	2	8	9	1	9	10066.3152	0.0012
6	3	4	6	2	5	10125.7900	-0.0069
6	3	3	6	2	5	10165.2155	0.0007
14	2	12	14	1	13	10220.3919	0.0014
7	3	5	7	2	6	10283.1126	0.0061
11	1	10	11	1	11	10340.6822	0.0032
16	4	12	16	3	13	10356.9493	-0.0008
7	3	4	7	2	6	10380.3118	0.0014
14	8	7	13	9	5	10396.2153	-0.0035
14	8	6	13	9	4		
8	3	6	8	2	7	10507.1512	0.0103
6	1	6	5	2	3	10541.4583	0.0021
9	4	6	8	5	4	10568.9050	0.0076
9	4	5	8	5	3	10589.2578	0.0014
8	3	5	8	2	7	10716.5470	0.0080
15	4	11	15	3	12	10803.7394	0.0035
5	1	4	4	2	2	10806.1958	0.0007
9	3	7	9	2	8	10808.4793	0.0073
3	1	3	2	0	2	10884.2384	-0.0008
6	1	6	5	2	4	10927.9136	-0.0019
4	0	4	3	1	3	10935.5252	0.0030
10	2	9	10	1	10	10967.7524	-0.0131
5	1	4	4	2	3	10975.9197	0.0037
10	3	8	10	2	9	11195.5893	-0.0029
9	3	6	9	2	8	11214.5403	-0.0066
8	2	6	8	1	8	11219.8187	0.0002
13	7	6	12	8	4	11231.3723	0.0082
13	7	7	12	8	5		

Table S3: Observed Transition Frequencies (in MHz) for c-CHFCH(CHF_3)O

J'	K_a'	K_c'	J''	K_a''	K_c''	Observed	Obs - Calc
8	3	6	7	4	3	11357.0029	-0.0107
8	3	6	7	4	4	11359.6819	-0.0040
8	3	5	7	4	3	11566.4043	-0.0073
8	3	5	7	4	4	11569.0854	0.0015
15	2	13	15	1	14	11653.5865	0.0086
11	3	9	11	2	10	11674.4081	-0.0041
7	2	6	6	3	3	11723.3639	-0.0087
7	2	6	6	3	4	11762.7851	-0.0054
12	1	11	12	0	12	11845.8975	0.0045
10	3	7	10	2	9	11917.7613	0.0098
3	1	2	2	0	2	11932.7026	-0.0001
11	2	10	11	1	11	11938.0315	0.0030
2	2	1	1	1	0	12147.1102	0.0005
2	2	0	1	1	0	12158.6428	0.0014
4	1	4	3	1	3	12187.4719	-0.0032
12	4	9	12	3	9	12205.5517	0.0068
2	2	1	1	1	1	12321.9373	0.0025
2	2	0	1	1	1	12333.4677	0.0013
12	4	8	12	3	9	12399.2415	0.0039
4	0	4	3	0	3	12446.4179	0.0025
5	0	5	4	1	3	12484.7701	0.0016
4	2	3	3	2	2	12549.8160	0.0022
4	3	2	3	3	1	12580.4769	0.0142
4	3	1	3	3	0	12583.3200	0.0052
7	1	7	6	2	4	12639.7181	-0.0053
4	2	2	3	2	1	12662.2092	0.0021
11	4	8	11	3	8	12745.2209	0.0114
11	4	7	11	3	8	12844.4865	0.0007
11	3	8	11	2	10	12866.7795	0.0047
4	1	3	3	1	2	12883.8998	-0.0032
11	5	7	10	6	5	12919.4609	0.0001
9	2	7	9	1	9	12936.1028	-0.0005
12	2	11	12	1	12	12966.8980	-0.0111
7	2	5	6	3	3	12992.4020	-0.0056
7	2	5	6	3	4	13031.8238	-0.0018
16	2	14	16	1	15	13149.0664	-0.0058
10	4	7	10	3	7	13150.5429	0.0037
13	1	12	13	1	13	13189.4521	-0.0026
10	4	6	10	3	7	13197.7292	-0.0044
13	1	12	13	0	13	13230.6390	0.0017
7	1	7	6	2	5	13383.4782	-0.0059
9	4	6	9	3	6	13436.7624	-0.0012
9	4	5	9	3	6	13457.2686	0.0022

Table S3: Observed Transition Frequencies (in MHz) for c-CHFCH(CHF_3)O

J'	K_a'	K_c'	J''	K_a''	K_c''	Observed	Obs - Calc
8	4	5	8	3	5	13626.7367	-0.0021
8	4	4	8	3	5	13634.6977	-0.0040
14	3	12	14	2	13	13675.7529	-0.0122
4	1	4	3	0	3	13698.3680	-0.0003
7	4	4	7	3	4	13745.3168	-0.0073
7	4	3	7	3	4	13747.9970	0.0005
10	4	7	9	5	5	13768.9470	0.0071
6	4	3	6	3	3	13814.8605	-0.0020
6	4	2	6	3	3	13815.5986	0.0039
10	4	6	9	5	5	13816.1274	-0.0069
8	4	5	8	3	6	13836.1289	-0.0079
7	4	4	7	3	5	13842.5263	-0.0017
9	4	6	9	3	7	13842.8299	-0.0086
8	4	4	8	3	6	13844.0949	-0.0049
7	4	3	7	3	5	13845.1987	-0.0016
5	4	2	5	3	2	13852.9513	-0.0023
6	4	3	6	3	4	13854.2849	0.0044
6	4	2	6	3	4	13855.0176	0.0050
9	4	5	9	3	7	13863.3427	0.0013
5	4	2	5	3	3	13866.1956	-0.0088
5	4	1	5	3	3	13866.3583	0.0071
4	4	1	4	3	1	13872.1649	-0.0110
10	4	7	10	3	8	13872.6985	-0.0001
10	4	6	10	3	8	13919.8926	-0.0004
11	4	8	11	3	9	13937.5746	0.0026
11	4	7	11	3	9	14036.8482	-0.0001
13	2	12	13	1	13	14044.0000	0.0092
12	4	9	12	3	10	14050.3113	0.0060
12	3	9	12	2	11	14092.6867	-0.0019
6	1	5	5	2	3	14173.2188	0.0023
5	0	5	4	1	4	14229.6598	0.0000
8	1	8	7	2	5	14466.2380	-0.0070
9	3	7	8	4	4	14511.1761	0.0101
6	1	5	5	2	4	14559.6772	0.0014
8	2	7	7	3	4	14597.8565	-0.0126
6	0	6	5	1	4	14885.0958	-0.0019
9	3	6	8	4	5	14925.2072	0.0033
3	2	2	2	1	1	15111.9715	0.0010
3	2	1	2	1	1	15169.3010	0.0028
5	1	5	4	1	4	15215.7227	-0.0042
4	1	3	3	0	3	15443.2644	0.0047
5	0	5	4	0	4	15481.6068	-0.0059
3	2	2	2	1	2	15636.4436	-0.0001

Table S3: Observed Transition Frequencies (in MHz) for c-CHFCH(CHF_3)O

J'	K_a'	K_c'	J''	K_a''	K_c''	Observed	Obs - Calc
5	2	4	4	2	3	15672.8255	0.0063
3	2	1	2	1	2	15693.7729	0.0016
5	3	3	4	3	2	15733.2727	0.0094
8	1	8	7	2	6	15735.2767	-0.0033
5	3	2	4	3	1	15743.1820	-0.0034
5	2	3	4	2	2	15889.5572	-0.0005
9	1	9	8	2	6	16010.7001	-0.0016
5	1	4	4	1	3	16079.8817	-0.0073
12	5	8	11	6	5	16124.6299	-0.0075
12	5	8	11	6	6	16124.7403	0.0129
5	1	5	4	0	4	16467.6780	-0.0019
11	4	8	10	5	5	16977.7516	0.0058
7	1	6	6	2	4	17437.3327	0.0012
18	3	16	18	2	17	17484.5038	-0.0040
6	0	6	5	1	5	17494.1547	0.0036
10	3	8	9	4	6	17659.4148	-0.0024
16	5	11	16	4	13	17732.2046	-0.0060
20	4	17	20	3	18	17861.0144	0.0129
9	1	9	8	2	7	17987.6232	0.0024
4	2	3	3	1	2	17987.8866	0.0106

Table S4: Observed Transition Frequencies (in MHz) for c-^{13}CHFCH(CHF_3)O

J'	K_a'	K_c'	J''	K_a''	K_c''	Observed	Obs - Calc
2	1	2	1	1	1	6051.6575	0.0007
2	0	2	1	0	1	6211.4693	-0.0010
2	2	1	2	1	2	6234.3879	0.0013
2	2	0	2	1	2	6245.3487	0.0006
8	1	7	8	0	8	6338.6312	-0.0041
2	1	1	1	1	0	6393.2001	0.0004
3	2	2	3	1	3	6497.1706	0.0003
3	2	1	3	1	3	6551.6789	-0.0010
11	3	9	11	2	9	6645.7970	0.0035
11	2	9	11	1	10	6668.0382	0.0164
4	2	3	4	1	4	6850.8124	0.0033
4	2	2	4	1	4	7012.2945	-0.0019
3	0	3	2	1	1	7030.2235	-0.0004
4	1	3	3	2	1	7235.5390	-0.0024
5	2	4	5	1	5	7296.8877	-0.0047
10	3	8	10	2	8	7384.8924	0.0016
3	0	3	2	1	2	7542.5389	-0.0002
5	2	3	5	1	5	7665.0400	0.0006
6	2	5	6	1	6	7836.0675	-0.0062
7	3	5	6	4	3	7933.7747	0.0003
2	1	2	1	0	1	7959.0142	-0.0002
7	3	4	6	4	2	8023.1650	0.0037
9	3	7	9	2	7	8050.7703	0.0017
5	1	5	4	2	3	8234.6782	0.0014
7	2	6	7	1	7	8467.4605	-0.0067
2	1	1	1	0	1	8471.3309	0.0012
6	2	4	6	1	6	8545.8062	-0.0004
6	2	5	5	3	3	8560.7486	0.0053
8	3	6	8	2	6	8624.4417	-0.0005
8	3	5	8	2	6	8818.6487	-0.0005
3	1	3	2	1	2	9070.8239	-0.0023
7	3	5	7	2	5	9091.5545	0.0013
7	3	4	7	2	5	9181.6038	0.0047
8	2	7	8	1	8	9188.2091	0.0044
6	2	4	5	3	2	9258.2167	0.0006
3	0	3	2	0	2	9290.0824	-0.0009
3	2	2	2	2	1	9333.6100	0.0001
3	2	1	2	2	0	9377.1572	-0.0009
6	3	4	6	2	4	9445.1701	-0.0010
6	3	3	6	2	4	9481.6560	-0.0028
3	1	2	2	1	1	9582.6958	0.0011
7	2	5	7	1	7	9681.0106	-0.0023
5	3	3	5	2	3	9688.9507	0.0006

Table S4: Observed Transition Frequencies (in MHz) for c-$^{13}CHFCH(CHF_3)O$

J'	K_a'	K_c'	J''	K_a''	K_c''	Observed	Obs - Calc
5	3	2	5	2	3	9701.2116	0.0014
4	0	4	3	1	2	9785.3498	0.0017
4	3	2	4	2	2	9838.0342	-0.0047
4	3	1	4	2	2	9841.1162	-0.0017
3	3	1	3	2	1	9915.8522	0.0010
3	3	0	3	2	1	9916.2938	0.0018
3	3	1	3	2	2	9970.3579	-0.0028
3	3	0	3	2	2	9970.7979	-0.0037
9	2	8	9	1	9	9993.2434	-0.0088
4	3	2	4	2	3	9999.5242	-0.0019
4	3	1	4	2	3	10002.6060	0.0008
5	3	2	5	2	4	10069.3551	-0.0021
6	3	4	6	2	5	10154.8938	-0.0101
6	3	3	6	2	5	10191.3992	0.0076
7	3	5	7	2	6	10305.1025	0.0035
7	3	4	7	2	6	10395.1483	0.0034
6	1	6	5	2	3	10413.6246	0.0049
8	3	6	8	2	7	10519.3242	0.0052
5	1	4	4	2	2	10622.4451	-0.0005
8	3	5	8	2	7	10713.5308	0.0048
6	1	6	5	2	4	10781.7661	-0.0006
5	1	4	4	2	3	10783.9260	-0.0069
9	3	7	9	2	8	10807.9277	0.0027
4	0	4	3	1	3	10809.5395	0.0078
3	1	3	2	0	2	10818.3712	0.0009
8	2	6	8	1	8	11083.0896	0.0081
8	3	5	7	4	3	11264.8575	-0.0059
7	2	6	6	3	4	11538.5354	0.0043
13	4	10	13	3	10	11706.1102	0.0033
3	1	2	2	0	2	11842.5544	0.0004
10	3	7	10	2	9	11851.9006	-0.0114
4	1	4	3	1	3	12082.6072	0.0012
2	2	1	1	1	0	12115.2712	0.0001
2	2	0	1	1	0	12126.2348	0.0023
2	2	1	1	1	1	12286.0460	0.0026
2	2	0	1	1	1	12297.0073	0.0024
4	0	4	3	0	3	12337.8233	0.0044
5	0	5	4	1	3	12371.5013	0.0031
4	2	3	3	2	2	12436.2474	0.0026
4	3	2	3	3	1	12465.4133	0.0031
4	3	1	3	3	0	12468.0475	-0.0008
7	1	7	6	2	4	12516.2316	-0.0035
12	4	8	12	3	9	12528.7093	0.0001

Table S4: Observed Transition Frequencies (in MHz) for c-^{13}CHFCH(CHF_3)O

J'	K_a'	K_c'	J''	K_a''	K_c''	Observed	Obs - Calc
4	2	2	3	2	1	12543.2198	-0.0026
7	2	5	6	3	3	12715.5855	-0.0038
9	2	7	9	1	9	12750.4128	0.0042
11	3	8	11	2	10	12753.5598	-0.0027
4	1	3	3	1	2	12763.0366	-0.0010
11	4	8	11	3	8	12866.1275	-0.0038
7	1	7	6	2	5	13225.9680	0.0001
10	4	7	10	3	7	13249.0272	0.0042
10	4	6	9	5	4	13445.7781	0.0043
9	4	6	9	3	6	13518.3510	-0.0048
9	4	5	9	3	6	13536.8191	-0.0129
4	1	4	3	0	3	13610.8952	0.0020
8	4	5	8	3	5	13696.7433	0.0030
7	4	4	7	3	4	13808.0395	-0.0014
7	4	3	7	3	4	13810.4457	-0.0013
6	4	3	6	3	3	13873.3687	0.0006
6	4	2	6	3	3	13874.0314	0.0043
8	4	5	8	3	6	13890.9546	0.0073
9	4	6	9	3	7	13895.6231	-0.0029
8	4	4	8	3	6	13898.1250	0.0056
7	4	3	7	3	5	13900.4912	-0.0017
5	4	2	5	3	2	13909.2146	-0.0066
6	4	2	6	3	4	13910.5176	0.0028
9	4	5	9	3	7	13914.0996	-0.0027
5	4	1	5	3	3	13921.6210	0.0076
4	4	1	4	3	1	13927.3470	-0.0131
6	1	5	5	2	3	13963.2095	0.0008
10	4	6	10	3	8	13964.1224	-0.0077
11	4	8	11	3	9	13980.0064	0.0031
11	4	7	11	3	9	14069.6162	-0.0115
5	0	5	4	1	4	14076.1117	-0.0018
6	1	5	5	2	4	14331.3479	-0.0078
6	0	6	5	1	4	14766.3403	-0.0043
5	1	5	4	1	4	15085.4868	0.0010
3	2	1	2	1	1	15110.1911	0.0002
4	1	3	3	0	3	15315.4984	-0.0100
5	0	5	4	0	4	15349.1921	0.0043
5	2	4	4	2	3	15531.5677	-0.0015
3	2	2	2	1	2	15567.9938	-0.0027
8	1	8	7	2	6	15568.7178	-0.0067
5	3	3	4	3	2	15589.1306	-0.0095
5	3	2	4	3	1	15598.3235	0.0024
5	2	3	4	2	2	15738.2303	0.0015

Table S4: Observed Transition Frequencies (in MHz) for c-^{13}CHFCH(CHF_3)O

J'	K_a'	K_c'	J''	K_a''	K_c''	Observed	Obs - Calc
5	1	4	4	1	3	15930.1226	-0.0041
5	1	5	4	0	4	16358.5542	-0.0060
7	0	7	6	1	5	16959.9464	-0.0137
7	1	6	6	2	4	17207.5256	-0.0114
9	1	9	8	2	7	17813.9483	0.0070
9	5	4	9	4	6	17837.6818	0.0161
4	2	3	3	1	2	17909.2404	0.0090
7	1	6	6	2	5	17917.2779	0.0080
4	2	2	3	1	2	18070.7300	0.0113
6	1	6	5	1	5	18078.6742	0.0152

Table S5: Observed Transition Frequencies (in MHz) for c-CHF^{13}CH(CHF_3)O

J'	K_a'	K_c'	J''	K_a''	K_c''	Observed	Obs - Calc
2	1	2	1	1	1	6094.9411	-0.0047
2	2	1	2	1	2	6160.0471	0.0035
5	2	3	4	3	1	6166.7871	-0.0021
2	2	0	2	1	2	6171.6804	0.0008
2	0	2	1	0	1	6258.0847	0.0003
11	3	9	11	2	9	6395.6914	0.0075
3	2	2	3	1	3	6429.2266	-0.0021
8	1	7	8	0	8	6436.5478	0.0090
2	1	1	1	1	0	6444.4901	0.0019
3	2	1	3	1	3	6487.0707	0.0013
11	2	9	11	1	10	6752.7806	-0.0006
4	2	3	4	1	4	6791.5689	0.0033
4	2	2	4	1	4	6962.7685	0.0037
3	0	3	2	1	1	7118.6068	0.0002
10	3	8	10	2	8	7150.4418	-0.0026
5	2	4	5	1	5	7248.6556	-0.0118
4	1	3	3	2	1	7425.1909	-0.0006
4	1	3	3	2	2	7483.0323	0.0002
5	2	3	5	1	5	7638.3328	0.0029
3	0	3	2	1	2	7642.9231	0.0020
6	2	5	6	1	6	7801.0965	-0.0001
9	3	7	9	2	7	7834.4330	0.0036
2	1	2	1	0	1	7973.5247	0.0021
9	3	6	9	2	7	8247.3668	-0.0087
7	3	5	6	4	3	8291.6596	0.0047
5	1	5	4	2	3	8399.6294	0.0052
8	3	6	8	2	6	8427.1746	-0.0001
7	2	6	7	1	7	8447.7303	0.0060
2	1	1	1	0	1	8497.8389	0.0018
6	2	4	6	1	6	8550.5736	0.0016
8	3	5	8	2	6	8640.2589	0.0117
6	2	5	5	3	3	8827.9669	0.0043
7	3	5	7	2	5	8912.7655	-0.0030
7	3	4	7	2	5	9011.7182	-0.0013
3	1	3	2	1	2	9135.3585	0.0004
8	2	7	8	1	8	9185.2794	0.0028
6	3	4	6	2	4	9282.6395	-0.0028
6	3	3	6	2	4	9322.7812	0.0016
3	0	3	2	0	2	9358.3571	-0.0023
3	2	2	2	2	1	9404.5438	0.0006
3	2	1	2	2	0	9450.7473	-0.0005
5	3	3	5	2	3	9539.0630	0.0004
5	3	2	5	2	3	9552.5545	-0.0029

Table S5: Observed Transition Frequencies (in MHz) for c-CHF^{13}CH(CHF_3)O

J'	K_a'	K_c'	J''	K_a''	K_c''	Observed	Obs - Calc
6	2	4	5	3	2	9563.9331	-0.0101
6	2	4	5	3	3	9577.4304	-0.0076
3	1	2	2	1	1	9659.1737	-0.0075
4	3	2	4	2	2	9696.5752	-0.0003
4	3	1	4	2	2	9699.9633	-0.0025
7	2	5	7	1	7	9725.5658	-0.0003
3	3	1	3	2	1	9779.0157	-0.0020
3	3	0	3	2	1	9779.5035	0.0002
3	3	0	3	2	2	9837.3452	0.0013
4	3	1	4	2	3	9871.1658	0.0010
4	0	4	3	1	2	9885.4340	-0.0035
5	3	3	5	2	4	9928.7120	-0.0132
5	3	2	5	2	4	9942.2241	0.0042
6	3	4	6	2	5	10032.1183	0.0007
6	3	3	6	2	5	10072.2537	-0.0012
7	3	5	7	2	6	10190.6239	0.0135
7	3	4	7	2	6	10289.5655	0.0041
8	3	6	8	2	7	10416.2210	0.0082
8	3	5	8	2	7	10629.2907	0.0054
5	1	4	4	2	2	10836.4407	-0.0029
3	1	3	2	0	2	10850.7941	-0.0022
4	0	4	3	1	3	10933.5727	-0.0025
6	1	6	5	2	4	10955.5896	-0.0015
5	1	4	4	2	3	11007.6454	0.0027
10	3	8	10	2	9	11108.8312	-0.0098
9	3	6	9	2	8	11132.4267	0.0017
8	2	6	8	1	8	11174.3076	-0.0071
13	4	10	13	3	10	11371.0151	-0.0102
8	3	6	7	4	4	11454.7205	0.0158
7	2	6	6	3	3	11781.5198	-0.0033
7	2	6	6	3	4	11821.6609	0.0006
3	1	2	2	0	2	11898.9372	0.0032
2	2	1	1	1	0	12080.2155	-0.0018
2	2	0	1	1	0	12091.8564	0.0031
4	1	4	3	1	3	12167.9621	0.0032
2	2	1	1	1	1	12254.9886	-0.0008
2	2	0	1	1	1	12266.6287	0.0032
4	0	4	3	0	3	12426.0083	-0.0039
5	0	5	4	1	3	12476.8963	-0.0043
4	2	3	3	2	2	12530.2935	-0.0025
4	3	2	3	3	1	12561.2247	0.0127
4	3	1	3	3	0	12564.1152	-0.0016
11	4	8	11	3	8	12597.7655	-0.0040

Table S5: Observed Transition Frequencies (in MHz) for c-$CHF^{13}CH(CHF_3)O$

J'	K_a'	K_c'	J''	K_a''	K_c''	Observed	Obs - Calc
4	2	2	3	2	1	12643.6507	-0.0035
11	3	8	11	2	10	12800.3006	-0.0034
4	1	3	3	1	2	12864.1227	-0.0005
9	2	7	9	1	9	12893.2160	-0.0115
10	4	7	10	3	7	13007.5823	0.0076
7	2	5	6	3	3	13059.3595	-0.0056
7	2	5	6	3	4	13099.5111	0.0088
9	4	6	9	3	6	13297.3594	-0.0023
9	4	5	9	3	6	13318.4333	0.0023
7	1	7	6	2	5	13406.0519	-0.0018
8	4	5	8	3	5	13489.8447	-0.0067
8	4	4	8	3	5	13498.0484	0.0125
7	4	4	7	3	4	13610.0246	-0.0039
7	4	3	7	3	4	13612.7676	-0.0080
4	1	4	3	0	3	13660.3946	-0.0012
6	4	3	6	3	3	13680.4686	-0.0101
6	4	2	6	3	3	13681.2377	0.0064
8	4	5	8	3	6	13702.9190	-0.0048
7	4	4	7	3	5	13708.9725	-0.0070
8	4	4	8	3	6	13711.1080	-0.0003
7	4	3	7	3	5	13711.7267	0.0001
5	4	2	5	3	2	13719.0344	-0.0088
5	4	1	5	3	2	13719.1995	0.0054
6	4	3	6	3	4	13720.6143	-0.0015
6	4	2	6	3	4	13721.3698	0.0013
9	4	5	9	3	7	13731.3773	0.0002
5	4	1	5	3	3	13732.6852	-0.0037
10	4	6	10	3	8	13789.8216	0.0063
12	3	9	12	2	11	14036.1017	0.0009
6	1	5	5	2	3	14195.8641	0.0004
5	0	5	4	1	4	14221.2041	0.0017
6	1	5	5	2	4	14585.5197	-0.0065
6	0	6	5	1	4	14870.5199	-0.0063
3	2	2	2	1	1	15040.2633	-0.0090
3	2	1	2	1	1	15098.1144	0.0015
5	1	5	4	1	4	15191.1915	0.0017
5	0	5	4	0	4	15455.5887	0.0025
3	2	2	2	1	2	15564.5882	0.0015
5	2	4	4	2	3	15648.2930	0.0014
5	3	3	4	3	2	15709.2467	0.0045
5	3	2	4	3	1	15719.3483	0.0017
5	2	3	4	2	2	15866.7570	0.0020
5	1	4	4	1	3	16054.9078	0.0013

Table S5: Observed Transition Frequencies (in MHz) for c-CHF^{13}CH(CHF_3)O

J'	K_a'	K_c'	J''	K_a''	K_c''	Observed	Obs - Calc
5	1	5	4	0	4	16425.5690	-0.0046
7	1	6	6	2	4	17450.9647	-0.0015
6	0	6	5	1	5	17478.5568	0.0121
9	5	5	9	4	5	17569.7625	-0.0039
9	5	4	9	4	5	17570.2950	0.0082
8	5	4	8	4	4	17612.7297	0.0141
4	2	3	3	1	2	17911.3854	-0.0016
9	1	9	8	2	7	18000.2643	-0.0090
4	2	2	3	1	2	18082.5929	0.0069

Table S6: Observed Transition Frequencies (in MHz) for c-CHFCH(^{13}CHF$_3$)O

J'	K_a'	K_c'	J''	K_a''	K_c''	Observed	Obs - Calc
2	1	2	1	1	1	6090.9083	0.0036
2	2	1	2	1	2	6227.6988	0.0092
2	2	0	2	1	2	6239.0938	0.0001
2	0	2	1	0	1	6253.5266	0.0014
8	1	7	8	0	8	6430.4762	-0.0080
2	1	1	1	1	0	6438.9462	-0.0003
3	2	2	3	1	3	6495.6108	-0.0006
11	3	9	11	2	9	6541.3722	-0.0012
3	2	1	3	1	3	6552.3076	-0.0011
11	2	9	11	1	10	6753.5257	-0.0045
4	2	3	4	1	4	6856.2136	0.0020
4	2	2	4	1	4	7024.0921	-0.0020
3	0	3	2	1	1	7090.7652	0.0002
10	3	8	10	2	8	7293.7155	0.0014
5	2	4	5	1	5	7311.1045	0.0019
4	1	3	3	2	1	7344.9023	-0.0012
4	1	3	3	2	2	7401.6067	0.0058
3	0	3	2	1	2	7612.8258	-0.0030
9	1	8	9	0	9	7680.7721	-0.0051
5	2	3	5	1	5	7693.4823	-0.0037
6	2	5	6	1	6	7860.8939	0.0022
9	3	7	9	2	7	7973.7629	-0.0032
2	1	2	1	0	1	7992.7816	0.0011
7	3	5	6	4	3	8112.9005	0.0027
7	3	4	6	4	2	8207.7320	0.0117
5	1	5	4	2	3	8325.7273	0.0008
9	3	6	9	2	7	8373.1628	0.0002
7	2	6	7	1	7	8504.5577	0.0053
2	1	1	1	0	1	8514.8454	0.0011
8	3	6	8	2	6	8561.6016	0.0017
6	2	4	6	1	6	8597.1111	-0.0032
6	2	5	5	3	3	8701.8867	-0.0038
8	3	5	8	2	6	8767.4598	-0.0070
7	3	5	7	2	5	9041.8953	0.0009
3	1	3	2	1	2	9129.4324	-0.0017
7	3	4	7	2	5	9137.4343	0.0028
8	2	7	8	1	8	9238.9677	-0.0053
3	0	3	2	0	2	9352.0832	-0.0010
3	2	2	2	2	1	9397.3583	0.0024
6	3	4	6	2	4	9406.7526	-0.0014
6	2	4	5	3	2	9425.0877	-0.0058
3	2	1	2	2	0	9442.6462	-0.0029
6	3	3	6	2	4	9445.4898	0.0009

Table S6: Observed Transition Frequencies (in MHz) for c-CHFCH($^{13}CHF_3$)O

J'	K_a'	K_c'	J''	K_a''	K_c''	Observed	Obs - Calc
3	1	2	2	1	1	9651.0268	0.0034
5	3	2	5	2	3	9672.0983	0.0007
7	2	5	7	1	7	9761.3638	-0.0061
4	3	2	4	2	2	9813.7726	-0.0018
4	3	1	4	2	2	9817.0431	-0.0017
4	0	4	3	1	2	9858.3521	0.0057
3	3	1	3	2	1	9894.6370	-0.0046
3	3	0	3	2	1	9895.1073	-0.0027
3	3	1	3	2	2	9951.3349	-0.0039
3	3	0	3	2	2	9951.8101	0.0029
4	3	2	4	2	3	9981.6529	-0.0039
4	3	1	4	2	3	9984.9264	-0.0009
5	3	3	5	2	4	10041.4643	0.0031
5	3	2	5	2	4	10054.4772	-0.0037
9	2	8	9	1	9	10058.7765	-0.0100
6	3	4	6	2	5	10142.9695	-0.0071
6	3	3	6	2	5	10181.7139	0.0024
7	3	5	7	2	6	10298.7171	0.0052
7	3	4	7	2	6	10394.2519	0.0029
6	1	6	5	2	3	10500.0819	0.0052
8	3	6	8	2	7	10520.5709	-0.0128
5	1	4	4	2	2	10755.0740	-0.0073
9	3	7	9	2	8	10819.1295	0.0047
3	1	3	2	0	2	10868.6870	-0.0024
6	1	6	5	2	4	10882.4612	0.0011
4	0	4	3	1	3	10902.0027	0.0033
5	1	4	4	2	3	10922.9616	-0.0023
8	2	6	8	1	8	11197.9617	0.0050
9	3	6	9	2	8	11218.5164	-0.0049
8	3	6	7	4	4	11273.6848	0.0042
8	3	5	7	4	3	11476.9455	0.0068
13	4	10	13	3	10	11582.8766	-0.0060
7	2	6	6	3	4	11695.8010	-0.0050
3	1	2	2	0	2	11912.3433	0.0008
10	3	7	10	2	9	11913.5201	-0.0060
11	2	10	11	1	11	11923.0878	0.0029
2	2	1	1	1	0	12144.5714	-0.0010
2	2	0	1	1	0	12155.9800	0.0036
4	1	4	3	1	3	12160.2906	-0.0034
12	4	9	12	3	9	12253.8042	0.0074
2	2	1	1	1	1	12318.5959	0.0015
2	2	0	1	1	1	12329.9991	0.0008
4	0	4	3	0	3	12418.6069	0.0022

Table S6: Observed Transition Frequencies (in MHz) for c-CHFCH($^{13}CHF_3$)O

J'	K_a'	K_c'	J''	K_a''	K_c''	Observed	Obs - Calc
12	4	8	12	3	9	12443.0669	0.0020
5	0	5	4	1	3	12452.3665	-0.0005
4	2	3	3	2	2	12520.8912	-0.0030
4	3	2	3	3	1	12551.2227	0.0106
4	3	1	3	3	0	12554.0131	-0.0012
7	1	7	6	2	4	12597.9973	-0.0104
4	2	2	3	2	1	12632.0746	-0.0048
11	4	8	11	3	8	12787.4167	-0.0051
11	3	8	11	2	10	12851.9631	-0.0008
4	1	3	3	1	2	12853.5615	0.0023
9	2	7	9	1	9	12904.1580	0.0129
7	2	5	6	3	3	12913.8911	0.0025
7	2	5	6	3	4	12952.6148	-0.0087
10	4	7	10	3	7	13187.6862	-0.0044
7	1	7	6	2	5	13334.2223	-0.0079
9	4	6	9	3	6	13470.0874	0.0164
8	4	5	8	3	5	13657.3895	-0.0058
10	4	7	9	5	5	13659.2863	-0.0009
8	4	4	8	3	5	13665.1635	-0.0057
4	1	4	3	0	3	13676.8963	-0.0029
7	4	4	7	3	4	13774.3165	0.0052
7	4	3	7	3	4	13776.9273	0.0073
6	4	2	6	3	3	13843.6015	0.0017
7	4	3	7	3	5	13872.4614	0.0044
5	4	2	5	3	2	13880.4628	-0.0021
6	4	3	6	3	4	13881.6278	0.0078
6	4	2	6	3	4	13882.3387	0.0041
9	4	5	9	3	7	13889.4838	-0.0024
5	4	1	5	3	3	13893.6323	0.0044
10	4	7	10	3	8	13898.3843	-0.0050
4	4	1	4	3	1	13899.4344	-0.0062
10	4	6	10	3	8	13944.4834	0.0059
11	4	7	11	3	9	14058.7196	0.0032
12	3	9	12	2	11	14065.1054	0.0000
6	1	5	5	2	3	14115.6514	-0.0025
5	0	5	4	1	4	14189.2889	0.0036
6	0	6	5	1	4	14850.2699	-0.0067
3	2	2	2	1	1	15102.9837	0.0020
3	2	1	2	1	1	15159.6790	0.0000
5	1	5	4	1	4	15181.9398	0.0017
4	1	3	3	0	3	15413.8137	-0.0038
5	0	5	4	0	4	15447.5815	0.0018
5	2	4	4	2	3	15636.8287	-0.0004

Table S6: Observed Transition Frequencies (in MHz) for c-CHFCH($^{13}CHF_3$)O

J'	K_a'	K_c'	J''	K_a''	K_c''	Observed	Obs - Calc
3	2	1	2	1	2	15681.7448	0.0020
8	1	8	7	2	6	15682.6889	0.0048
5	3	3	4	3	2	15696.6347	0.0012
5	3	2	4	3	1	15706.3800	-0.0028
5	2	3	4	2	2	15851.3321	0.0020
5	1	4	4	1	3	16042.2595	0.0023
5	1	5	4	0	4	16440.2268	-0.0058
7	1	6	6	2	4	17374.7887	-0.0059
6	0	6	5	1	5	17447.5270	0.0130
7	5	3	7	4	3	17848.1837	0.0000
4	2	3	3	1	2	17972.8508	-0.0018

Table S7: Observed Transition Frequencies (in MHz) for c-CHFCH(CHF_3)^{18}O

J'	K_a'	K_c'	J''	K_a''	K_c''	Observed	Obs - Calc
2	0	2	1	0	1	6177.8114	-0.0017
2	1	1	1	1	0	6374.7131	0.0026
3	2	1	3	1	3	6405.6094	0.0032
4	2	2	4	1	4	6914.8427	-0.0032
4	1	3	3	2	1	7417.7265	-0.0088
3	0	3	2	1	2	7569.8237	0.0044
5	2	3	5	1	5	7640.8989	-0.0023
2	1	2	1	0	1	7842.3230	-0.0057
8	3	6	8	2	6	8096.5491	0.0106
5	1	5	4	2	3	8243.8838	-0.0046
2	1	1	1	0	1	8393.6592	-0.0029
7	3	5	7	2	5	8621.3295	0.0024
6	2	4	6	1	6	8622.3476	0.0092
6	2	5	5	3	3	8767.3255	0.0133
3	1	3	2	1	2	9002.7809	-0.0088
6	3	4	6	2	4	9025.9315	0.0005
3	0	3	2	0	2	9234.3272	-0.0077
3	2	2	2	2	1	9286.3622	-0.0063
5	3	3	5	2	3	9309.6177	-0.0017
4	3	2	4	2	2	9485.4731	0.0000
3	1	2	2	1	1	9553.5279	-0.0012
3	3	1	3	2	1	9578.0598	-0.0049
3	3	0	3	2	2	9643.8294	0.0020
4	3	1	4	2	3	9682.1017	-0.0073
4	0	4	3	1	2	9719.3898	-0.0029
5	3	3	5	2	4	9746.3844	0.0056
5	3	2	5	2	4	9762.6962	0.0065
7	2	5	7	1	7	9884.2770	-0.0061
6	3	3	6	2	5	9910.4588	0.0061
7	3	4	7	2	6	10157.7894	-0.0062
3	1	3	2	0	2	10667.3046	-0.0006
6	1	6	5	2	4	10727.4294	0.0035
5	1	4	4	2	2	10791.6388	-0.0051
4	0	4	3	1	3	10821.4726	0.0068
9	3	6	9	2	8	11117.2348	0.0024
2	2	1	1	1	0	11880.2313	0.0023
2	2	0	1	1	0	11893.3520	-0.0001
4	1	4	3	1	3	11989.6821	-0.0074
2	2	1	1	1	1	12064.0061	-0.0016
2	2	0	1	1	1	12077.1268	-0.0039
5	0	5	4	1	3	12230.9486	0.0024
4	0	4	3	0	3	12254.4312	-0.0048
4	3	2	3	3	1	12406.3454	0.0079

Table S7: Observed Transition Frequencies (in MHz) for c-CHFCH(CHF_3)^{18}O

J'	K_a'	K_c'	J''	K_a''	K_c''	Observed	Obs - Calc
4	2	2	3	2	1	12498.9270	-0.0021
10	4	7	10	3	7	12615.7811	-0.0132
4	1	3	3	1	2	12721.2609	-0.0077
7	1	7	6	2	5	13099.6901	0.0141
8	4	5	8	3	5	13174.1308	0.0033
6	4	3	6	3	3	13396.0671	-0.0040
4	1	4	3	0	3	13422.6614	0.0016
5	4	2	5	3	2	13440.7080	0.0009
6	4	2	6	3	4	13445.5047	0.0087
6	1	5	5	2	3	14101.4216	0.0144
3	2	1	2	1	1	14857.0565	-0.0059
5	0	5	4	0	4	15232.8120	-0.0100
3	2	2	2	1	2	15343.2240	0.0034
8	1	8	7	2	6	15363.2216	-0.0054
5	2	4	4	2	3	15448.0304	0.0003
5	3	3	4	3	2	15516.4113	0.0105
5	3	2	4	3	1	15528.6131	0.0023
5	2	3	4	2	2	15692.2550	0.0005
5	1	4	4	1	3	15872.8393	0.0016
5	1	5	4	0	4	16134.4247	0.0017
7	0	7	6	1	5	16612.6875	-0.0127
8	2	6	7	3	4	16640.0213	-0.0122
6	0	6	5	1	5	17270.9053	0.0109
4	2	2	3	1	2	17802.4640	0.0017
6	1	6	5	1	5	17931.5734	0.0058

Table S8: Observed Transition Frequencies (in MHz) for t-CHFCH(CHF_3)O

J'	K_a'	K_c'	J''	K_a''	K_c''	Observed	Obs - Calc
11	1	10	11	0	11	5765.2776	-0.0013
11	7	5	12	6	6	5857.4655	-0.0025
11	7	4	12	6	7		
10	3	8	9	4	5	5949.3416	0.0015
10	3	7	9	4	6	5975.5583	0.0023
6	1	6	5	2	3	6128.3258	0.0002
8	2	7	7	3	4	6352.2314	0.0004
13	8	6	14	7	7	6363.5458	0.0097
13	8	5	14	7	8		
12	1	11	12	0	12	6401.9524	-0.0003
8	2	6	7	3	5	6624.8925	-0.0002
15	9	7	16	8	8	6869.5565	-0.0073
15	9	6	16	8	9		
4	4	1	5	3	2	6906.4885	0.0014
4	4	0	5	3	3	6906.9119	-0.0037
17	6	12	16	7	9	7006.6757	-0.0058
17	6	11	16	7	10		
12	2	10	12	1	11	7075.6700	0.0027
13	1	12	13	0	13	7110.3537	-0.0010
11	2	9	11	1	10	7126.2144	0.0008
14	2	12	14	1	13	7133.3534	0.0035
4	0	4	3	1	2	7207.0755	-0.0006
10	2	8	10	1	9	7218.8843	0.0016
15	2	13	15	1	14	7255.5572	0.0009
9	2	7	9	1	8	7344.5323	0.0008
6	5	2	7	4	3	7413.4453	-0.0014
6	5	1	7	4	4		
16	2	14	16	1	15	7447.9607	0.0020
7	2	6	7	1	6	7492.6088	-0.0103
8	2	6	8	1	7	7493.4333	-0.0028
15	5	11	14	6	8	7516.3897	0.0048
15	5	10	14	6	9	7516.6276	0.0029
3	1	3	2	1	2	7585.5004	0.0008
6	1	5	5	2	3	7601.1687	-0.0170
4	0	4	3	1	3	7628.2499	-0.0010
6	1	5	5	2	4	7646.8976	-0.0028
7	2	5	7	1	6	7655.6495	-0.0023
3	0	3	2	0	2	7686.3702	-0.0017
3	2	2	2	2	1	7691.5988	0.0017
3	2	1	2	2	0	7696.8365	-0.0015
17	2	15	17	1	16	7715.6039	0.0017
6	2	5	6	1	5	7730.3529	0.0145
3	1	2	2	1	1	7796.0752	-0.0036

Table S8: Observed Transition Frequencies (in MHz) for t-CHFCH(CHF_3)O

J'	K_a'	K_c'	J''	K_a''	K_c''	Observed	Obs - Calc
6	2	4	6	1	5	7821.4335	0.0005
2	1	2	1	0	1	7841.4475	-0.0001
14	1	13	14	0	14	7887.3283	-0.0044
8	6	3	9	5	4	7919.9464	-0.0047
8	6	2	9	5	5		
5	2	4	5	1	4	7935.9297	0.0014
5	2	3	5	1	4	7981.6432	0.0003
13	4	10	12	5	7	8027.2507	-0.0017
13	4	9	12	5	8	8030.8574	0.0057
2	1	1	1	0	1	8052.0395	-0.0038
18	2	16	18	1	17	8062.7828	0.0022
4	2	3	4	1	3	8108.4560	-0.0001
4	2	2	4	1	3	8128.0874	-0.0027
3	2	2	3	1	2	8247.2168	-0.0017
3	2	1	3	1	2	8253.7698	-0.0006
2	2	1	2	1	1	8351.6879	-0.0123
2	2	0	2	1	1	8353.0098	-0.0013
7	1	7	6	2	4	8390.7511	-0.0001
10	7	4	11	6	5	8426.2852	0.0001
10	7	3	11	6	6		
19	2	17	19	1	18	8492.8892	-0.0027
11	3	9	10	4	6	8525.7122	0.0015
2	2	1	2	1	2	8562.2937	-0.0022
2	2	0	2	1	2	8563.6062	-0.0006
11	3	8	10	4	7	8571.5071	-0.0026
3	2	2	3	1	3	8668.3920	-0.0013
15	1	14	15	0	15	8727.6645	-0.0012
4	2	3	4	1	4	8810.3270	-0.0028
4	2	2	4	1	4	8829.9613	-0.0025
9	2	8	8	3	5	8874.8300	-0.0033
12	8	5	13	7	6	8932.5042	0.0062
12	8	4	13	7	7		
5	2	4	5	1	5	8988.4651	-0.0010
20	2	18	20	1	19	9008.1829	-0.0034
5	2	3	5	1	5	9034.1760	-0.0047
6	2	5	6	1	6	9203.1961	-0.0025
6	2	4	6	1	6	9294.2915	-0.0016
9	2	7	8	3	6	9300.4319	-0.0040
14	9	6	15	8	7	9438.6271	-0.0035
14	9	5	15	8	8		
7	2	6	7	1	7	9454.9246	0.0001
18	6	13	17	7	10	9584.7765	-0.0149
18	6	12	17	7	11		

Table S8: Observed Transition Frequencies (in MHz) for t-CHFCH(CHF_3)O

J'	K_a'	K_c'	J''	K_a''	K_c''	Observed	Obs - Calc
21	2	19	21	1	20	9609.4570	0.0005
16	1	15	16	0	16	9624.3467	0.0003
8	2	7	8	1	8	9744.0021	-0.0031
5	5	0	6	4	3	9980.5723	-0.0023
5	5	1	6	4	2		
9	2	8	9	1	9	10070.7197	-0.0038
16	5	12	15	6	9	10096.6013	-0.0017
16	5	11	15	6	10	10097.0702	0.0099
4	1	4	3	1	3	10112.4975	0.0010
4	0	4	3	0	3	10242.3930	0.0009
4	2	3	3	2	2	10254.4303	-0.0028
4	3	1	3	3	0	10258.0503	0.0138
4	3	2	3	3	1		
4	2	2	3	2	1	10267.5138	-0.0014
22	2	20	22	1	21	10295.7470	0.0078
3	1	3	2	0	2	10300.5109	-0.0021
5	0	5	4	1	4	10309.0007	0.0004
4	1	3	3	1	2	10393.1983	0.0028
10	2	9	10	1	10	10435.2444	0.0002
7	1	6	6	2	5	10444.1477	-0.0036
7	6	2	8	5	3	10487.4841	-0.0022
7	6	1	8	5	4		
17	1	16	17	0	17	10568.9917	-0.0019
8	1	8	7	2	5	10587.7272	-0.0006
14	4	11	13	5	8	10609.9992	-0.0058
14	4	10	13	5	9	10616.4720	-0.0041
3	1	2	2	0	2	10721.6856	-0.0022
22	3	19	22	2	20	10830.2751	0.0065
11	2	10	11	1	11	10837.5715	-0.0015
21	3	18	21	2	19	10970.8892	-0.0032
9	7	3	10	6	4	10994.1387	-0.0010
9	7	2	10	6	5		
12	3	10	11	4	7	11102.9600	0.0014
20	3	17	20	2	18	11165.0997	-0.0019
12	3	9	11	4	8	11179.0848	-0.0033
12	2	11	12	1	12	11277.5228	0.0008
10	2	9	9	3	6	11380.1533	-0.0036
19	3	16	19	2	17	11402.1137	0.0013
11	8	4	12	7	5	11500.6065	-0.0004
11	8	3	12	7	6		
18	1	17	18	0	18	11552.3473	-0.0010
18	3	15	18	2	16	11670.4250	0.0004
13	2	12	13	1	13	11754.6800	0.0000

Table S8: Observed Transition Frequencies (in MHz) for t-CHFCH(CHF_3)O

J'	K_a'	K_c'	J''	K_a''	K_c''	Observed	Obs - Calc
6	0	6	5	1	4	11956.0026	0.0050
17	3	14	17	2	15	11958.2095	-0.0017
13	9	5	14	8	6	12006.9298	-0.0009
13	9	4	14	8	7		
10	2	8	9	3	7	12012.5508	-0.0023
16	3	13	16	2	14	12253.7866	-0.0002
14	2	13	14	1	14	12268.3918	0.0001
15	3	12	15	2	13	12546.1110	0.0007
19	1	18	19	0	19	12564.8157	0.0016
5	1	5	4	1	4	12638.2506	0.0001
17	5	13	16	6	10	12679.7105	0.0040
17	5	12	16	6	11	12680.5446	0.0005
9	1	9	8	2	6	12710.8324	-0.0009
4	1	4	3	0	3	12726.6376	0.0000
5	0	5	4	0	4	12793.2484	0.0025
5	2	4	4	2	3	12816.3902	0.0034
15	2	14	15	1	15	12817.7461	-0.0003
5	4	1	4	4	0	12822.1384	0.0070
5	4	2	4	4	1		
5	3	3	4	3	2	12823.6208	0.0044
5	3	2	4	3	1	12823.9366	-0.0011
14	3	11	14	2	12	12825.2853	0.0025
5	2	3	4	2	2	12842.4662	-0.0012
5	1	4	4	1	3	12988.9166	0.0019
6	0	6	5	1	5	13008.5370	0.0015
6	6	1	7	5	2	13054.1036	-0.0013
6	6	0	7	5	3		
13	3	10	13	2	11	13083.0044	-0.0001
15	4	12	14	5	9	13196.1627	-0.0042
15	4	11	14	5	10	13207.3256	-0.0005
8	1	7	7	2	6	13270.7851	0.0010
12	3	9	12	2	10	13312.9533	0.0019
16	2	15	16	1	16	13401.5752	-0.0022
4	1	3	3	0	3	13428.5083	-0.0031
11	3	8	11	2	9	13511.0292	0.0031
2	2	1	1	1	0	13549.6402	-0.0010
2	2	0	1	1	0	13550.9596	0.0074
8	7	2	9	6	3	13561.1634	-0.0012
8	7	1	9	6	4		
20	1	19	20	0	20	13597.0036	0.0032
2	2	1	1	1	1	13619.8514	0.0115
2	2	0	1	1	1	13621.1511	0.0002
10	3	8	10	2	8	13649.4118	-0.0021

Table S8: Observed Transition Frequencies (in MHz) for t-CHFCH(CHF_3)O

J'	K_a'	K_c'	J''	K_a''	K_c''	Observed	Obs - Calc
10	3	7	10	2	8	13675.4374	-0.0019
13	3	11	12	4	8	13680.1346	-0.0024
13	3	10	12	4	9	13801.4729	-0.0044
9	3	6	9	2	7	13806.5843	-0.0015
11	2	10	10	3	7	13863.6497	-0.0048
8	3	5	8	2	6	13906.7218	-0.0017
7	3	5	7	2	5	13976.2801	-0.0148
7	3	4	7	2	5	13979.5012	-0.0007
17	2	16	17	1	17	14018.4727	0.0000
6	3	4	6	2	4	14028.1516	0.0167
6	3	3	6	2	4	14029.4190	-0.0001
5	3	3	5	2	3	14060.8614	-0.0052
5	3	2	5	2	3	14061.2920	-0.0029
10	8	3	11	7	4	14067.9566	-0.0030
10	8	2	11	7	5		
4	3	1	4	2	2	14079.8271	0.0024
3	3	0	3	2	1	14089.2511	0.0074
3	3	1	3	2	2	14095.7725	-0.0078
4	3	2	4	2	3	14099.3504	-0.0012
5	3	3	5	2	4	14106.5797	-0.0014
5	3	2	5	2	4	14106.9984	-0.0111
6	3	4	6	2	5	14119.2278	-0.0017
6	3	3	6	2	5	14120.5024	-0.0112
7	3	5	7	2	6	14139.3248	-0.0027
8	3	6	8	2	7	14169.1364	0.0027
9	3	7	9	2	8	14211.0847	-0.0019
10	3	8	10	2	9	14267.7517	-0.0012
11	3	9	11	2	10	14341.7751	0.0015
12	3	10	12	2	11	14435.8131	0.0036
13	3	11	13	2	12	14552.4878	0.0016
12	9	4	13	8	5	14574.5456	-0.0035
12	9	3	13	8	6		
21	1	20	21	0	21	14640.2232	-0.0031
18	2	17	18	1	18	14666.7953	0.0001
14	3	12	14	2	13	14694.3400	-0.0031
10	1	10	9	2	7	14751.4986	0.0001
11	2	9	10	3	8	14765.7820	-0.0003
15	3	13	15	2	14	14863.7773	-0.0058
16	3	14	16	2	15	15063.0400	0.0104
5	1	5	4	0	4	15122.4949	-0.0013
6	1	6	5	1	5	15162.5078	0.0014
18	5	14	17	6	11	15265.9928	-0.0023
18	5	13	17	6	12	15267.4861	0.0112

Table S8: Observed Transition Frequencies (in MHz) for t-CHFCH(CHF_3)O

J'	$K_a{}'$	$K_c{}'$	J''	$K_a{}''$	$K_c{}''$	Observed	Obs - Calc
17	3	15	17	2	16	15294.0860	0.0007
6	0	6	5	0	5	15337.7898	0.0041
19	2	18	19	1	19	15344.7274	0.0146
6	2	5	5	2	4	15377.2412	0.0025
6	5	2	5	5	1	15386.2561	0.0050
6	5	1	5	5	0		
6	4	2	5	4	1	15387.5046	0.0040
6	4	3	5	4	2		
6	3	4	5	3	3	15389.8905	0.0034
6	3	3	5	3	2	15390.7449	0.0019
6	2	4	5	2	3	15422.6189	0.0001
18	3	16	18	2	17	15558.6991	0.0007
6	1	5	5	1	4	15582.8253	-0.0034
7	0	7	6	1	6	15721.0768	0.0013
16	4	13	15	5	10	15785.8972	-0.0013
16	4	12	15	5	11	15804.4513	-0.0050
3	2	2	2	1	1	16043.2966	-0.0007
3	2	1	2	1	1	16049.8502	0.0010
20	2	19	20	1	20	16050.2445	0.0097
9	1	8	8	2	7	16125.0384	0.0003
7	7	0	8	6	3	16127.4863	0.0212
7	7	1	8	6	2		
5	1	4	4	0	4	16175.0349	0.0008
20	3	18	20	2	19	16194.1523	0.0105
3	2	2	2	1	2	16253.8946	0.0017
14	3	12	13	4	9	16255.9046	-0.0012
3	2	1	2	1	2	16260.4439	-0.0009
12	2	11	11	3	8	16320.0880	-0.0005
14	3	11	13	4	10	16442.4378	0.0017
21	3	19	21	2	20	16566.9428	-0.0125
11	1	11	10	2	8	16701.5185	-0.0024
21	2	20	21	1	21	16781.2535	-0.0011
23	3	21	23	2	22	17425.1885	-0.0128
6	1	6	5	0	5	17491.7585	0.0019
22	2	21	22	1	22	17535.5862	-0.0076
12	2	10	11	3	9	17563.4898	0.0087
7	1	7	6	1	6	17685.0452	0.0009
19	5	14	18	6	13	17858.3068	0.0059
7	0	7	6	0	6	17875.0465	-0.0001
7	2	6	6	2	5	17936.7720	0.0016
7	6	2	6	6	1	17950.3499	0.0036
7	6	1	6	6	0		

Table S8: Observed Transition Frequencies (in MHz) for t-CHFCH(CHF_3)O

J'	K_a'	K_c'	J''	K_a''	K_c''	Observed	Obs - Calc
7	5	3	6	5	2	17951.4288	0.0015
7	5	2	6	5	1		
7	4	3	6	4	2	17953.3831	-0.0014
7	4	4	6	4	3		
7	3	5	6	3	4	17956.8684	0.0000
7	3	4	6	3	3	17958.7891	-0.0022
7	2	5	6	2	4	18008.7082	-0.0003

Table S9: Observed Transition Frequencies (in MHz) for t-^{13}CHFCH(CHF_3)O

J'	K_a'	K_c'	J''	K_a''	K_c''	Observed	Obs - Calc
6	1	6	5	2	3	6038.6710	0.0006
13	1	12	13	0	13	7023.9166	0.0027
13	2	11	13	1	12	7081.3879	0.0061
12	2	10	12	1	11	7088.0550	-0.0080
11	2	9	11	1	10	7143.3754	0.0032
10	2	8	10	1	9	7239.1065	-0.0038
9	2	7	9	1	8	7366.3116	0.0015
8	2	6	8	1	7	7515.4705	-0.0035
6	1	5	5	2	4	7529.4733	0.0017
3	1	3	2	1	2	7540.1136	-0.0016
4	0	4	3	1	3	7558.6894	-0.0005
3	0	3	2	0	2	7639.2765	-0.0018
7	2	5	7	1	6	7676.9266	-0.0002
2	1	2	1	0	1	7815.2523	-0.0017
6	2	4	6	1	5	7841.2198	0.0011
5	2	3	5	1	4	7999.5097	-0.0019
4	2	2	4	1	3	8143.8982	0.0012
3	2	1	3	1	2	8267.6173	-0.0020
2	2	0	2	1	1	8365.2117	0.0012
2	2	1	2	1	2	8570.8023	0.0001
3	2	2	3	1	3	8674.9997	-0.0002
4	2	3	4	1	4	8814.3856	-0.0013
5	2	4	5	1	5	8989.3131	0.0014
9	2	7	8	3	6	9130.0445	0.0073
6	2	5	6	1	6	9200.1577	-0.0013
7	2	6	7	1	7	9447.3170	0.0008
8	2	7	8	1	8	9731.1383	0.0008
9	2	8	9	1	9	10051.9014	-0.0043
4	1	4	3	1	3	10052.0401	0.0021
4	0	4	3	0	3	10179.8275	0.0057
5	0	5	4	1	4	10222.0339	-0.0017
3	1	3	2	0	2	10260.4101	0.0000
7	1	6	6	2	5	10307.0518	-0.0108
4	1	3	3	1	2	10327.7528	-0.0014
10	2	9	10	1	10	10409.7985	0.0069
10	2	9	9	3	6	11211.3364	0.0053
13	2	12	13	1	13	11705.4215	-0.0045
10	2	8	9	3	7	11821.4626	-0.0030
5	1	5	4	1	4	12562.7498	-0.0120
9	1	9	8	2	6	12598.2593	-0.0042
4	1	4	3	0	3	12673.1718	0.0019
5	2	4	4	2	3	12737.6816	-0.0051
5	3	3	4	3	2	12744.6643	0.0093

Table S9: Observed Transition Frequencies (in MHz) for t-^{13}CHFCH(CHF_3)O

J'	K_a'	K_c'	J''	K_a''	K_c''	Observed	Obs - Calc
5	2	3	4	2	2	12762.8170	-0.0063
14	3	11	14	2	12	12878.0250	0.0049
6	0	6	5	1	5	12904.1262	-0.0002
5	1	4	4	1	3	12907.2145	0.0058
8	1	7	7	2	6	13113.6368	-0.0009
13	3	10	13	2	11	13130.2352	-0.0033
2	2	1	1	1	0	13529.1182	-0.0003
11	3	8	11	2	9	13547.2007	0.0006
2	2	1	1	1	1	13598.0700	-0.0004
2	2	0	1	1	1	13599.3371	0.0035
10	3	7	10	2	8	13706.7413	-0.0037
9	3	6	9	2	7	13833.7596	-0.0011
8	3	5	8	2	6	13930.5993	0.0049
7	3	4	7	2	5	14000.8857	-0.0026
6	3	3	6	2	4	14049.0586	-0.0012
4	3	1	4	2	2	14097.6670	-0.0004
4	3	2	4	2	3	14116.4841	-0.0026
5	3	3	5	2	4	14123.4541	-0.0009
7	3	5	7	2	6	14155.0384	0.0067
8	3	6	8	2	7	14183.7891	0.0037
9	3	7	9	2	8	14224.2706	-0.0005
10	3	8	10	2	9	14278.9728	-0.0040
5	1	5	4	0	4	15056.1118	0.0018
6	1	6	5	1	5	15072.0438	0.0045
6	0	6	5	0	5	15244.8602	0.0076
6	2	5	5	2	4	15282.8932	0.0067
6	3	3	5	3	2	15295.8888	-0.0013
6	2	4	5	2	3	15326.6304	-0.0027
7	0	7	6	1	6	15599.3717	-0.0020
9	1	8	8	2	7	15947.5095	-0.0031
3	2	2	2	1	1	16008.2615	0.0013
3	2	1	2	1	2	16221.4264	-0.0023
6	1	6	5	0	5	17412.7665	0.0010
7	1	7	6	1	6	17579.6578	0.0010
7	0	7	6	0	6	17767.2838	-0.0029

Table S10: Observed Transition Frequencies (in MHz) for t-$CHF^{13}CH(CHF_3)O$

J'	K_a'	K_c'	J''	K_a''	K_c''	Observed	Obs - Calc
11	1	10	11	0	11	5707.1529	-0.0100
6	1	6	5	2	3	6156.3103	0.0011
12	1	11	12	0	12	6333.0756	0.0008
8	2	6	7	3	5	6649.0881	-0.0037
13	2	11	13	1	12	7051.4398	-0.0030
12	2	10	12	1	11	7055.5703	-0.0119
14	2	12	14	1	13	7103.7918	0.0106
11	2	9	11	1	10	7108.8615	0.0009
10	2	8	10	1	9	7203.0546	-0.0061
8	2	6	8	1	7	7477.6722	-0.0038
3	1	3	2	1	2	7581.5249	-0.0015
4	0	4	3	1	3	7627.1100	-0.0026
7	2	5	7	1	6	7638.8012	-0.0006
6	1	5	5	2	4	7651.9967	0.0110
3	0	3	2	0	2	7680.9663	0.0053
3	2	1	2	2	0	7691.1484	-0.0043
3	1	2	2	1	1	7789.0044	0.0057
6	2	4	6	1	5	7803.0348	0.0022
2	1	2	1	0	1	7830.4292	0.0006
5	2	3	5	1	4	7961.4506	0.0005
4	2	2	4	1	3	8106.0698	0.0014
3	2	1	3	1	2	8230.0573	-0.0057
2	2	0	2	1	1	8327.9083	-0.0007
7	1	7	6	2	4	8421.9480	-0.0020
2	2	1	2	1	2	8534.1219	0.0014
3	2	2	3	1	3	8638.6430	-0.0004
4	2	3	4	1	4	8778.4668	-0.0016
9	2	8	8	3	5	8905.3353	-0.0095
5	2	4	5	1	5	8953.9464	-0.0013
6	2	5	6	1	6	9165.4707	0.0020
9	2	7	8	3	6	9319.9990	-0.0049
7	2	6	7	1	7	9413.4235	0.0021
8	2	7	8	1	8	9698.1609	-0.0006
9	2	8	9	1	9	10019.9682	-0.0024
4	0	4	3	0	3	10235.3359	-0.0009
4	2	2	3	2	1	10259.7982	-0.0041
3	1	3	2	0	2	10289.1859	0.0007
5	0	5	4	1	4	10304.5561	0.0015
4	1	3	3	1	2	10383.7905	-0.0063
12	2	11	12	1	12	11208.6992	0.0017
14	2	13	14	1	14	12184.9230	0.0014
5	1	5	4	1	4	12631.7432	0.0058
4	1	4	3	0	3	12715.4573	-0.0046

Table S10: Observed Transition Frequencies (in MHz) for t-$CHF^{13}CH(CHF_3)O$

J'	K_a'	K_c'	J''	K_a''	K_c''	Observed	Obs - Calc
5	0	5	4	0	4	12784.6789	-0.0008
5	2	4	4	2	3	12807.2074	-0.0094
14	3	11	14	2	12	12807.5417	0.0035
5	4	1	4	4	0	12812.8174	0.0002
5	4	2	4	4	1		
5	3	3	4	3	2	12814.2579	-0.0005
5	3	2	4	3	1	12814.5627	-0.0049
5	2	3	4	2	2	12832.6065	-0.0100
5	1	4	4	1	3	12977.2350	0.0002
6	0	6	5	1	5	13000.6860	0.0006
13	3	10	13	2	11	13060.8984	-0.0056
8	1	7	7	2	6	13265.2306	0.0017
12	3	9	12	2	10	13286.5077	0.0011
11	3	8	11	2	9	13480.5052	0.0019
2	2	1	1	1	0	13519.8384	-0.0004
2	2	0	1	1	1	13590.2798	0.0015
10	3	7	10	2	8	13641.2940	-0.0028
9	3	6	9	2	7	13769.4002	-0.0020
8	3	5	8	2	6	13867.1220	-0.0024
11	2	10	10	3	7	13894.2117	0.0033
7	3	4	7	2	5	13938.0950	-0.0004
6	3	3	6	2	4	13986.7467	-0.0001
5	3	2	5	2	3	14017.8009	-0.0019
4	3	1	4	2	2	14035.8538	0.0021
3	3	0	3	2	1	14045.0327	0.0079
3	3	1	3	2	2	14051.3885	-0.0018
4	3	2	4	2	3	14054.8630	-0.0054
5	3	3	5	2	4	14061.9090	-0.0011
6	3	4	6	2	5	14074.2302	-0.0013
7	3	5	7	2	6	14093.8120	-0.0010
8	3	6	8	2	7	14122.8635	0.0049
9	3	7	9	2	8	14163.7529	0.0033
10	3	8	10	2	9	14218.9926	-0.0018
12	3	10	12	2	11	14382.9058	0.0016
5	1	5	4	0	4	15111.8636	0.0011
6	1	6	5	1	5	15154.7686	-0.0079
6	0	6	5	0	5	15327.8747	0.0065
6	2	5	5	2	4	15366.3014	0.0039
6	1	5	5	1	4	15568.9149	-0.0014
7	0	7	6	1	6	15709.8678	0.0034
3	2	2	2	1	1	16012.6868	0.0053
3	2	1	2	1	2	16226.5495	-0.0004
6	1	6	5	0	5	17481.9620	0.0027

Table S10: Observed Transition Frequencies (in MHz) for t-CHF^{13}CH(CHF_3)O

J'	K_a'	K_c'	J''	K_a''	K_c''	Observed	Obs - Calc
7	1	7	6	1	6	17676.1482	0.0087
7	0	7	6	0	6	17863.9540	-0.0015
7	2	6	6	2	5	17924.0915	-0.0007
7	4	4	6	4	3	17940.2693	0.0040
7	4	3	6	4	2		
7	3	4	6	3	3	17945.5320	0.0073

Table S11: Observed Transition Frequencies (in MHz) for t-CHFCH($^{13}CHF_3$)O

J'	K_a'	K_c'	J''	K_a''	K_c''	Observed	Obs - Calc
11	1	10	11	0	11	5751.4738	-0.0003
6	1	6	5	2	3	6083.8799	0.0003
13	2	11	13	1	12	7082.9061	-0.0077
12	2	10	12	1	11	7085.0920	-0.0105
11	2	9	11	1	10	7137.2063	-0.0002
14	2	12	14	1	13	7138.0487	0.0067
10	2	8	10	1	9	7230.9260	-0.0059
9	2	7	9	1	8	7357.1797	-0.0030
8	2	6	8	1	7	7506.2976	-0.0008
3	1	3	2	1	2	7566.6762	-0.0091
6	1	5	5	2	4	7594.9087	0.0018
4	0	4	3	1	3	7598.3454	0.0010
3	0	3	2	0	2	7667.0970	-0.0024
7	2	5	7	1	6	7668.4174	0.0064
3	2	2	2	2	1	7672.2647	-0.0036
3	2	1	2	2	0	7677.4504	-0.0026
3	1	2	2	1	1	7776.2467	-0.0066
2	1	2	1	0	1	7832.3943	0.0015
6	2	4	6	1	5	7833.8622	0.0014
14	1	13	14	0	14	7861.5550	0.0092
5	2	3	5	1	4	7993.5977	-0.0003
4	2	2	4	1	3	8139.5137	-0.0005
3	2	1	3	1	2	8264.6773	-0.0012
7	1	7	6	2	4	8341.6315	0.0021
2	2	0	2	1	1	8363.4781	-0.0007
2	2	1	2	1	2	8571.7668	0.0007
3	2	2	3	1	3	8677.3504	0.0012
4	2	3	4	1	4	8818.5947	-0.0003
5	2	4	5	1	5	8995.8571	-0.0038
6	2	5	6	1	6	9209.5358	-0.0039
9	2	7	8	3	6	9222.5112	0.0009
7	2	6	7	1	7	9460.0277	0.0014
8	2	7	8	1	8	9747.6788	-0.0011
9	2	8	9	1	9	10072.7822	-0.0017
4	1	4	3	1	3	10087.4292	0.0027
4	0	4	3	0	3	10216.7561	-0.0046
4	2	3	3	2	2	10228.6767	0.0043
4	2	2	3	2	1	10241.6138	-0.0004
5	0	5	4	1	4	10272.2281	0.0002
3	1	3	2	0	2	10285.5164	0.0008
4	1	3	3	1	2	10366.7830	0.0045
7	1	6	6	2	5	10384.6715	-0.0014
10	2	9	10	1	10	10435.5086	0.0035

Table S11: Observed Transition Frequencies (in MHz) for t-CHFCH($^{13}CHF_3$)O

J'	K_a'	K_c'	J''	K_a''	K_c''	Observed	Obs - Calc
11	2	10	11	1	11	10835.8455	-0.0094
10	2	9	9	3	6	11300.9005	0.0031
10	2	8	9	3	7	11926.7427	-0.0001
14	2	13	14	1	14	12259.7473	-0.0103
5	1	5	4	1	4	12606.9352	-0.0024
9	1	9	8	2	6	12653.8559	-0.0124
4	1	4	3	0	3	12705.8420	-0.0007
5	0	5	4	0	4	12761.3129	0.0029
5	2	4	4	2	3	12784.2032	-0.0003
5	3	3	4	3	2	12791.3515	-0.0044
5	3	2	4	3	1	12791.6627	-0.0090
5	2	3	4	2	2	12810.0078	0.0024
5	1	4	4	1	3	12955.9238	0.0021
6	0	6	5	1	5	12964.9005	0.0003
13	3	10	13	2	11	13108.2829	0.0115
8	1	7	7	2	6	13203.7215	0.0020
12	3	9	12	2	10	13336.6276	0.0000
11	3	8	11	2	9	13533.1584	0.0010
2	2	1	1	1	0	13546.8992	-0.0008
2	2	0	1	1	1	13618.0598	0.0013
10	3	7	10	2	8	13696.1687	0.0027
9	3	6	9	2	7	13826.1109	-0.0008
8	3	5	8	2	6	13925.2862	0.0021
7	3	4	7	2	5	13997.3315	-0.0022
6	3	3	6	2	4	14046.7357	-0.0018
5	3	2	5	2	3	14078.2808	0.0012
4	3	1	4	2	2	14096.6156	0.0025
3	3	0	3	2	1	14105.9307	-0.0011
4	3	2	4	2	3	14115.9371	0.0060
5	3	3	5	2	4	14123.0834	-0.0001
7	3	5	7	2	6	14155.4829	-0.0020
8	3	6	8	2	7	14184.9775	-0.0037
10	3	8	10	2	9	14282.5975	0.0049
12	3	10	12	2	11	14448.9768	-0.0007
5	1	5	4	0	4	15096.0195	-0.0002
6	2	5	5	2	4	15338.6464	0.0020
6	3	4	5	3	3	15351.1627	0.0039
6	3	3	5	3	2	15352.0045	0.0041
6	2	4	5	2	3	15383.5432	0.0007
6	1	5	5	1	4	15543.2784	-0.0012
7	0	7	6	1	6	15670.6350	-0.0018
3	2	2	2	1	1	16034.4487	-0.0016
3	2	1	2	1	2	16250.5168	0.0008

Table S11: Observed Transition Frequencies (in MHz) for t-CHFCH($^{13}CHF_3$)O

J'	K_a'	K_c'	J''	K_a''	K_c''	Observed	Obs - Calc
6	1	6	5	0	5	17459.6798	0.0044
7	0	7	6	0	6	17830.7038	0.0016
7	3	4	6	3	3	17913.5630	0.0061

Table S12: Observed Transition Frequencies (in MHz) for t-CHFCH(CHF_3)^{18}O

J'	K_a'	K_c'	J''	K_a''	K_c''	Observed	Obs - Calc
4	0	4	3	1	3	7620.3817	0.0003
2	1	2	1	0	1	7705.3929	0.0010
5	2	3	5	1	4	7714.6718	0.0013
4	2	2	4	1	3	7871.8172	0.0033
3	2	1	3	1	2	8008.1481	0.0057
2	2	0	2	1	1	8116.6011	0.0024
2	2	1	2	1	2	8346.9825	-0.0103
3	2	2	3	1	3	8464.0124	0.0042
4	2	3	4	1	4	8620.6156	0.0020
5	2	4	5	1	5	8817.2459	-0.0017
8	2	7	8	1	8	9651.9075	-0.0012
3	1	3	2	0	2	10128.8629	-0.0023
4	1	4	3	0	3	12516.3657	0.0011
5	0	5	4	0	4	12661.8465	0.0017
6	0	6	5	1	5	12967.3525	0.0055
2	2	1	1	1	0	13270.0497	-0.0009
2	2	0	1	1	1	13349.0330	0.0042
6	3	3	6	2	4	13636.8935	0.0049
5	3	2	5	2	3	13676.3909	0.0031
4	3	1	4	2	2	13699.4182	0.0058
4	3	2	4	2	3	13723.7319	-0.0071
5	3	3	5	2	4	13732.7361	-0.0060
6	3	4	6	2	5	13748.4626	-0.0009
8	3	6	8	2	7	13810.2870	0.0015
5	1	5	4	0	4	14871.2325	0.0021
3	2	2	2	1	1	15731.5569	-0.0065
3	2	1	2	1	2	15971.7639	0.0004
6	1	6	5	0	5	17198.0259	-0.0058
7	4	4	6	4	3	17780.0068	-0.0009
7	4	3	6	4	2		
7	3	4	7	2	5	13575.3006	-0.0013

Table S13: Observed Transition Frequencies (in MHz) for Ar-c-CHFCH(CHF_3)O

J'	K_a'	K_c'	J''	K_a''	K_c''	Observed	Obs - Calc
6	0	6	5	1	4	5612.8646	0.0035
2	2	1	1	1	1	5712.9326	-0.0017
3	1	2	2	0	2	5716.2539	-0.0049
4	2	3	3	2	2	5747.3030	0.0000
4	3	1	3	3	0	5807.7004	-0.0015
8	4	5	8	3	5	5825.6097	-0.0018
4	2	2	3	2	1	5929.6085	-0.0046
4	1	3	3	1	2	6031.0324	-0.0070
7	4	4	7	3	4	6090.3520	0.0024
7	0	7	6	1	5	6120.4846	0.0023
8	2	7	7	3	5	6151.9080	-0.0016
9	1	9	8	2	7	6239.8032	0.0026
6	4	3	6	3	3	6251.6806	0.0000
5	4	2	5	3	2	6338.8964	-0.0019
4	4	1	4	3	1	6380.7280	-0.0056
6	4	2	6	3	4	6382.7664	0.0023
5	4	1	5	3	3	6383.4179	0.0026
4	4	0	4	3	2	6391.9347	-0.0030
7	4	3	7	3	5	6406.2475	0.0012
8	4	4	8	3	6	6480.0369	-0.0010
5	0	5	4	1	4	6508.2753	-0.0029
8	0	8	7	1	6	6529.2205	0.0021
9	3	7	8	4	5	6606.2478	0.0011
13	5	9	13	4	9	6610.7520	-0.0022
6	1	5	5	2	3	6621.1289	-0.0018
9	4	5	9	3	7	6641.7363	0.0026
7	2	5	6	3	3	6651.6473	-0.0056
5	1	5	4	1	4	6740.0529	0.0001
10	4	6	9	5	4	6746.7043	0.0003
7	2	5	7	1	7	6816.3724	0.0059
10	1	10	9	2	8	6836.9392	0.0087
3	2	2	2	1	1	6840.4498	0.0008
5	0	5	4	0	4	6875.8899	-0.0031
9	0	9	8	1	7	6895.3201	0.0054
3	2	1	2	1	1	6938.0215	0.0004
10	4	6	10	3	8	6940.1625	0.0031
5	1	5	4	0	4	7107.6677	0.0002
12	5	8	12	4	8	7147.4254	0.0002
5	2	4	4	2	3	7159.1697	0.0010
9	2	8	8	3	6	7176.8971	-0.0055
6	1	5	5	2	4	7227.7821	-0.0012
5	4	2	4	4	1	7247.1383	0.0021
5	4	1	4	4	0	7247.9850	0.0035

Table S13: Observed Transition Frequencies (in MHz) for Ar-c-CHFCH(CHF_3)O

J'	K_a'	K_c'	J''	K_a''	K_c''	Observed	Obs - Calc
5	3	3	4	3	2	7256.5029	-0.0010
10	0	10	9	1	8	7263.1301	0.0012
5	3	2	4	3	1	7288.9677	-0.0038
3	2	2	2	1	2	7310.1605	-0.0002
3	2	1	2	1	2	7407.7367	0.0038
11	4	7	11	3	9	7430.9672	0.0002
5	2	3	4	2	2	7485.9365	-0.0027
5	1	4	4	1	3	7490.1768	-0.0039
4	1	3	3	0	3	7502.6141	-0.0046
11	5	7	11	4	7	7545.6770	-0.0011
9	3	6	8	4	4	7634.7739	-0.0030
11	0	11	10	1	9	7655.7736	-0.0008
10	5	6	10	4	6	7818.0300	-0.0017
7	1	6	6	2	4	7854.4744	-0.0028
6	0	6	5	1	5	7917.4335	-0.0039
12	1	12	11	2	10	7924.7507	-0.0057
10	3	8	9	4	6	7928.3058	-0.0002
11	4	8	10	5	6	7970.2133	-0.0021
9	5	5	9	4	5	7991.8859	0.0033
4	2	3	3	1	2	8043.9632	0.0008
6	1	6	5	1	5	8053.5965	-0.0006
12	0	12	11	1	10	8077.7211	0.0084
10	3	7	10	2	9	8078.6746	0.0008
8	5	4	8	4	4	8097.5510	0.0007
10	2	9	9	3	7	8099.1654	0.0006
10	5	5	10	4	7	8108.4977	0.0056
9	5	4	9	4	6	8121.7608	-0.0006
11	5	6	11	4	8	8130.1052	0.0032
8	5	3	8	4	5	8148.8691	0.0035
6	0	6	5	0	5	8149.2117	-0.0003
7	5	3	7	4	3	8159.9076	-0.0011
12	4	8	12	3	10	8165.7395	0.0052
7	5	2	7	4	4	8177.2882	0.0026
6	5	2	6	4	2	8196.0639	-0.0019
6	5	1	6	4	3	8200.8430	-0.0001
5	5	0	5	4	2	8217.6013	0.0149
12	5	7	12	4	9	8218.1068	-0.0039
8	2	6	8	1	8	8259.5099	-0.0060
6	1	6	5	0	5	8285.3733	0.0016
4	2	2	3	1	2	8323.8451	0.0004
8	2	6	7	3	4	8408.5633	-0.0017
13	5	8	13	4	10	8415.6016	-0.0057
13	1	13	12	2	11	8437.4952	-0.0126

Table S13: Observed Transition Frequencies (in MHz) for Ar-c-CHFCH(CHF_3)O

J'	K_a'	K_c'	J''	K_a''	K_c''	Observed	Obs - Calc
11	4	7	10	5	5	8490.2333	0.0027
6	2	5	5	2	4	8555.1298	-0.0008
6	5	2	5	5	1	8692.9959	0.0110
6	5	1	5	5	0		
6	4	3	5	4	2	8709.7745	0.0034
6	4	2	5	4	1	8713.5278	0.0011
6	3	4	5	3	3	8714.1764	-0.0014
14	5	9	14	4	11	8776.0070	-0.0013
6	3	3	5	3	2	8796.9879	-0.0008
8	1	7	7	2	5	8863.7701	0.0006
15	6	10	15	5	10	8894.8553	-0.0026
3	3	1	2	2	0	8902.4144	0.0037
3	3	0	2	2	0	8904.0116	0.0006
6	1	5	5	1	4	8909.6921	-0.0023
3	3	1	2	2	1	8922.3360	-0.0009
11	2	10	10	3	8	8923.3159	0.0029
3	3	0	2	2	1	8923.9427	0.0054
7	1	6	6	2	5	8952.0667	-0.0030
4	2	3	3	1	3	8981.7435	-0.0009
6	2	4	5	2	3	9046.0679	-0.0026
11	3	9	10	4	7	9167.7504	-0.0053
5	2	4	4	1	3	9172.0899	-0.0018
13	4	9	13	3	11	9177.3287	0.0060
13	5	9	12	6	7	9195.3226	-0.0047
4	2	2	3	1	3	9261.6356	0.0089
14	6	9	14	5	9	9279.8992	-0.0047
7	0	7	6	1	6	9281.1499	-0.0060
7	1	7	6	1	6	9357.0655	0.0013
5	1	4	4	0	4	9412.2406	-0.0032
7	0	7	6	0	6	9417.3147	-0.0009
12	4	9	11	5	7	9429.7863	0.0043
11	3	8	11	2	10	9469.4892	0.0034
7	1	7	6	0	6	9493.2259	0.0020
10	3	7	9	4	5	9536.1049	0.0001
13	6	8	13	5	8	9545.3511	-0.0008
9	1	8	8	2	6	9641.3709	-0.0006
12	6	7	12	5	7	9722.7489	0.0123
14	6	8	14	5	10	9766.8767	0.0022
5	2	3	4	1	3	9778.7446	0.0002
15	6	9	15	5	11	9790.4161	-0.0022
13	6	7	13	5	9	9790.8606	-0.0038
12	6	6	12	5	8	9836.9089	0.0033
11	6	6	11	5	6	9840.1166	-0.0045

Table S13: Observed Transition Frequencies (in MHz) for Ar-c-CHFCH(CHF_3)O

J'	K_a'	K_c'	J''	K_a''	K_c''	Observed	Obs - Calc
9	2	7	9	1	9	9876.2685	-0.0002
11	6	5	11	5	7	9888.6762	0.0058
16	6	10	16	5	12	9897.8865	-0.0198
10	6	5	10	5	5	9918.2474	0.0008
7	2	6	6	2	5	9933.2489	0.0002
9	6	3	9	5	5	9977.1456	-0.0004
8	6	3	8	5	3	10006.5199	0.0057
8	6	2	8	5	4	10008.3190	-0.0115
9	2	7	8	3	5	10054.0584	0.0002
17	6	11	17	5	13	10137.2595	-0.0064
7	6	2	6	6	1	10138.4863	-0.0073
7	6	1	6	6	0		
7	5	3	6	5	2	10153.9933	0.0069
7	5	2	6	5	1	10154.3273	-0.0057
19	7	13	19	6	13	10156.2455	0.0194
7	3	5	6	3	4	10166.6604	-0.0010
7	4	4	6	4	3	10177.8967	0.0062
7	4	3	6	4	2	10190.1484	0.0049
10	1	9	9	2	7	10210.2041	0.0017
6	2	5	5	1	4	10237.0438	0.0022
7	1	6	6	1	5	10279.4163	-0.0007
4	3	2	3	2	1	10300.7291	-0.0003
12	3	10	11	4	8	10310.4629	-0.0064
4	3	1	3	2	1	10311.8280	0.0010
13	2	12	12	3	10	10320.8570	-0.0037
7	3	4	6	3	3	10339.2185	-0.0031
12	4	8	11	5	6	10346.1070	0.0056
15	6	10	14	7	8	10346.6067	-0.0123
4	3	2	3	2	2	10398.3002	-0.0013
4	3	1	3	2	2	10409.3994	0.0003
14	4	10	14	3	12	10470.4050	0.0040
7	2	5	6	2	4	10587.8195	0.0021
8	0	8	7	1	7	10612.2465	0.0018
11	1	10	10	2	8	10619.9207	-0.0061
8	1	8	7	1	7	10652.9855	0.0012
8	0	8	7	0	7	10688.1476	-0.0055
5	2	4	4	1	4	10726.5371	-0.0030
8	1	8	7	0	7	10728.9018	0.0092
14	5	10	13	6	8	10733.4765	-0.0003
13	4	10	12	5	8	10839.4701	-0.0026
14	2	13	13	3	11	10923.2741	-0.0031
12	1	11	11	2	9	10934.9621	-0.0026
17	7	11	17	6	11	11013.9130	-0.0042

Table S13: Observed Transition Frequencies (in MHz) for Ar-c-CHFCH(CHF_3)O

J'	K_a'	K_c'	J''	K_a''	K_c''	Observed	Obs - Calc
7	2	6	6	1	5	11260.5979	0.0020
16	7	10	16	6	10	11266.7999	-0.0014
8	2	7	7	2	6	11292.7047	0.0015
6	2	4	5	1	4	11334.6323	-0.0019
13	3	11	12	4	9	11348.0983	0.0007
15	7	9	15	6	9	11443.9251	-0.0132
6	1	5	5	0	5	11446.0395	-0.0058
11	3	8	10	4	6	11452.6415	0.0022
16	7	9	16	6	11	11463.8889	-0.0059
15	2	14	14	3	12	11481.6453	0.0083
10	2	8	9	3	6	11515.9667	0.0000
14	1	13	13	2	11	11517.7193	-0.0058
15	7	8	15	6	10	11537.8335	-0.0066
14	7	8	14	6	8	11569.4541	0.0027
8	7	2	7	7	1	11583.7244	0.0077
8	7	1	7	7	0		
8	1	7	7	1	6	11597.1081	-0.0016
8	6	3	7	6	2	11597.7884	0.0009
8	6	2	7	6	1		
8	3	6	7	3	5	11609.0269	0.0012
14	7	7	14	6	9	11611.1722	0.0035
10	2	8	10	1	10	11616.9443	-0.0040
8	5	4	7	5	3	11620.4639	0.0050
8	5	3	7	5	2	11621.8245	-0.0016
5	3	3	4	2	2	11627.6189	-0.0013
8	4	5	7	4	4	11650.2485	0.0024
13	7	7	13	6	7	11660.1116	-0.0048
5	3	2	4	2	2	11671.1850	-0.0004
13	7	6	13	6	8	11677.2699	0.0060
8	4	4	7	4	3	11682.8167	-0.0006
12	7	6	12	6	6	11726.8447	0.0018
12	7	5	12	6	7	11733.2875	-0.0022
11	7	5	11	6	5	11776.5341	-0.0047
11	7	4	11	6	6	11778.7210	0.0015
10	7	4	10	6	4	11813.6301	0.0032
10	7	3	10	6	5	11814.2741	-0.0007
5	3	3	4	2	3	11907.5011	-0.0014
8	3	5	7	3	4	11914.9841	-0.0002
16	6	11	15	7	9	11920.1412	-0.0022
9	0	9	8	1	8	11922.4615	-0.0049
9	1	9	8	1	8	11943.7248	0.0000
5	3	2	4	2	3	11951.0691	0.0014
9	0	9	8	0	8	11963.2067	0.0007

Table S13: Observed Transition Frequencies (in MHz) for Ar-c-CHFCH(CHF_3)O

J'	K_a'	K_c'	J''	K_a''	K_c''	Observed	Obs - Calc
9	1	9	8	0	8	11984.4683	0.0039
16	2	15	15	3	13	12009.2464	0.0098
8	2	6	7	2	5	12096.1347	0.0011
16	6	10	15	7	8	12097.7651	-0.0069
14	4	11	13	5	9	12179.2381	-0.0028
4	4	0	3	3	0	12188.5452	0.0034
4	4	1	3	3	1	12190.0332	-0.0026
9	1	8	8	2	7	12196.9629	-0.0004
15	5	11	14	6	9	12253.5714	-0.0076
8	2	7	7	1	6	12273.8904	0.0082
14	3	12	13	4	10	12278.9268	0.0021
13	4	9	12	5	7	12307.3169	0.0072
6	2	5	5	1	5	12541.6143	-0.0037
9	2	8	8	2	7	12634.0175	-0.0012
11	2	9	10	3	7	12740.5435	-0.0016
6	3	4	5	2	3	12855.8581	-0.0008
9	1	8	8	1	7	12873.7373	0.0016
6	3	3	5	2	3	12982.2359	0.0009
7	2	5	6	1	5	13012.7541	-0.0031
15	5	10	14	6	8	13021.2228	0.0102
9	8	2	8	8	1	13028.5369	-0.0010
9	8	1	8	8	0		
9	3	7	8	3	6	13036.7531	0.0001
9	7	2	8	7	1	13041.6601	0.0032
9	7	3	8	7	2		
9	5	5	8	5	4	13092.7909	0.0096
9	5	4	8	5	3	13097.1381	0.0028
15	3	13	14	4	11	13107.7361	-0.0093
9	4	6	8	4	5	13124.2415	0.0020
9	4	5	8	4	4	13198.4504	0.0014
10	0	10	9	1	9	13220.2912	-0.0003
10	1	10	9	1	9	13231.1503	0.0017
10	0	10	9	0	9	13241.5489	-0.0011
10	1	10	9	0	9	13252.4123	0.0052
17	8	10	17	7	10	13287.4925	-0.0041
12	3	9	11	4	7	13304.4956	0.0005
9	2	8	8	1	7	13310.7878	-0.0034
16	8	9	16	7	9	13388.5717	-0.0128
16	8	8	16	7	10	13403.1378	0.0043
15	4	12	14	5	10	13431.0639	0.0035
6	3	4	5	2	4	13462.5092	-0.0024
15	8	8	15	7	8	13467.5284	0.0020
9	3	6	8	3	5	13509.9191	-0.0008

Table S13: Observed Transition Frequencies (in MHz) for Ar-c-CHFCH(CHF_3)O

J'	K_a'	K_c'	J''	K_a''	K_c''	Observed	Obs - Calc
14	8	7	14	7	7	13529.8645	-0.0022
9	2	7	8	2	6	13560.4763	-0.0013
7	1	6	6	0	6	13576.2455	-0.0048
13	8	5	13	7	7	13579.9964	-0.0041
6	3	3	5	2	4	13588.8758	-0.0119
5	4	2	4	3	1	13627.8715	0.0017
5	4	1	4	3	1	13628.8253	0.0037
5	4	2	4	3	2	13638.9635	-0.0040
6	2	4	5	1	5	13639.2147	0.0041
5	4	1	4	3	2	13639.9353	0.0161
11	8	4	11	7	4	13648.8303	0.0162
11	8	3	11	7	5		
10	8	3	10	7	3	13672.3363	-0.0021
10	8	2	10	7	4		
10	1	9	9	2	8	13692.2516	-0.0014
12	2	10	11	3	8	13700.2446	0.0024
16	5	12	15	6	10	13737.7105	0.0047
16	3	14	15	4	12	13844.8240	-0.0086
10	2	9	9	2	8	13959.0134	-0.0019
7	3	5	6	2	4	13976.4562	0.0064
10	1	9	9	1	8	14129.3082	-0.0002
7	3	4	6	2	4	14275.3848	-0.0012
14	4	10	13	5	8	14333.7144	-0.0039
10	2	9	9	1	8	14396.0681	-0.0026
13	2	11	12	3	9	14398.8138	-0.0017
7	2	6	6	1	6	14421.2632	-0.0064
10	3	8	9	3	7	14446.2950	-0.0037
11	2	9	10	3	8	14472.0856	0.0007
10	8	3	9	8	2	14485.3659	0.0013
10	8	2	9	8	1		
10	7	4	9	7	3	14503.1504	0.0039
10	7	3	9	7	2		
11	1	11	10	1	10	14516.5487	0.0002
11	0	11	10	0	10	14521.9561	0.0022
10	6	5	9	6	4	14530.1167	-0.0002
10	6	4	9	6	3	14530.5942	0.0032
10	5	6	9	5	5	14570.8762	0.0028
16	4	13	15	5	11	14581.1939	0.0018
10	5	5	9	5	4	14582.7133	-0.0045
10	4	7	9	4	6	14595.9865	-0.0006
10	4	6	9	4	5	14744.7218	-0.0025
8	2	6	7	1	6	14829.4699	-0.0039
10	2	8	9	2	7	14971.8215	-0.0068

Table S13: Observed Transition Frequencies (in MHz) for Ar-c-CHFCH(CHF_3)O

J'	K_a'	K_c'	J''	K_a''	K_c''	Observed	Obs - Calc
8	3	6	7	2	5	14997.6591	0.0010
16	5	11	15	6	9	14999.3050	0.0016
13	3	10	12	4	8	15005.8384	0.0015
6	4	2	5	3	2	15053.3866	0.0099
7	3	5	6	2	5	15074.0378	-0.0046
6	4	3	5	3	3	15092.2344	-0.0002
6	4	2	5	3	3	15096.9383	-0.0037
10	3	7	9	3	6	15099.7779	0.0010
11	1	10	10	2	9	15114.7852	-0.0051
17	5	13	16	6	11	15165.8013	-0.0007
15	2	13	14	3	11	15184.2368	-0.0101
11	2	10	10	2	9	15270.4463	-0.0006
7	3	4	6	2	5	15372.9740	-0.0046
11	1	10	10	1	9	15381.5526	0.0000
5	5	0	4	4	0	15464.6073	-0.0090
5	5	1	4	4	1	15464.7116	-0.0047
11	2	10	10	1	9	15537.2193	0.0101
8	3	5	7	2	5	15602.5546	0.0016
17	4	14	16	5	12	15621.8463	0.0113
12	1	12	11	1	11	15800.7511	0.0117
12	0	12	11	0	11	15803.4922	0.0012
12	1	12	11	0	11	15806.1868	-0.0045
11	3	9	10	3	8	15835.4339	-0.0030
11	8	3	10	8	2	15945.1260	0.0110
11	8	4	10	8	3		
11	6	6	10	6	5	16004.2270	0.0174
11	6	5	10	6	4	16005.7002	0.0027
11	5	7	10	5	6	16053.8906	-0.0002
11	4	8	10	4	7	16060.7244	-0.0009
11	5	6	10	5	5	16082.3347	-0.0006
11	2	9	10	2	8	16324.3532	-0.0022
11	4	7	10	4	6	16326.2413	-0.0031
15	4	11	14	5	9	16354.2792	0.0013
8	2	7	7	1	7	16356.9024	-0.0061
7	4	4	6	3	3	16429.5719	0.0007
7	4	3	6	3	3	16446.5337	0.0022
14	3	11	13	4	9	16481.1952	-0.0002
12	1	11	11	2	10	16483.7358	-0.0009
7	4	4	6	3	4	16555.9455	-0.0019
12	2	11	11	2	10	16571.4420	-0.0029
7	4	3	6	3	4	16572.9032	-0.0045
12	1	11	11	1	10	16639.3939	0.0005
11	3	8	10	3	7	16661.2591	0.0003

Table S13: Observed Transition Frequencies (in MHz) for Ar-c-CHFCH(CHF_3)O

J'	K_a'	K_c'	J''	K_a''	K_c''	Observed	Obs - Calc
8	3	6	7	2	6	16749.8175	-0.0019
9	2	7	8	1	7	16792.8345	-0.0073
6	5	1	5	4	1	16909.6697	0.0073
6	5	2	5	4	2	16910.5481	0.0039
9	3	6	8	2	6	17016.3383	-0.0009
13	1	13	12	1	12	17084.2027	0.0064
13	0	13	12	0	12	17085.5725	-0.0017
12	3	10	11	3	9	17203.4367	-0.0022
12	8	5	11	8	4	17408.1345	0.0128
12	8	4	11	8	3		
12	6	7	11	6	6	17484.0945	0.0129
12	6	6	11	6	5	17488.1882	-0.0003
12	4	9	11	4	8	17513.4532	-0.0042
12	5	8	11	5	7	17539.9522	-0.0011
12	5	7	11	5	6	17601.4644	-0.0016
12	2	10	11	2	9	17620.9559	0.0001
15	3	12	14	4	10	17678.5518	0.0016
8	4	5	7	3	4	17740.5966	0.0008
8	4	4	7	3	4	17790.1247	-0.0026
13	2	12	12	2	11	17864.9777	-0.0009
13	1	12	12	1	11	17904.5882	-0.0083
12	4	8	11	4	7	17938.2052	-0.0010
9	1	8	8	0	8	17941.6240	-0.0030
8	4	5	7	3	5	18039.5321	0.0000

Table S14: Observed Transition Frequencies (in MHz) for Ar-c-$^{13}CHFCH(CHF_3)O$

J'	K_a'	K_c'	J''	K_a''	K_c''	Observed	Obs - Calc
5	0	5	4	0	4	6856.0291	0.0124
3	2	2	2	1	2	7269.3030	-0.0099
5	2	3	4	2	2	7483.3877	0.0036
4	1	3	3	0	3	7492.2544	0.0044
6	1	6	5	1	5	8031.9942	0.0073
6	0	6	5	0	5	8124.5117	-0.0085
4	2	2	3	1	2	8282.1992	0.0063
6	2	5	5	2	4	8539.4491	-0.0029
3	3	0	2	2	0	8835.2645	0.0053
3	3	1	2	2	1	8854.2232	-0.0045
6	1	5	5	1	4	8894.1482	-0.0099
4	2	3	3	1	3	8941.4245	0.0009
6	2	4	5	2	3	9042.3198	-0.0005
7	1	7	6	1	6	9331.0794	0.0023
5	1	4	4	0	4	9406.0873	0.0057
5	2	3	4	1	3	9740.9785	0.0032
7	2	6	6	2	5	9913.3381	-0.0022
7	3	5	6	3	4	10153.6114	-0.0091
4	3	1	3	2	1	10240.0312	-0.0073
7	1	6	6	1	5	10256.7161	-0.0071
4	3	2	3	2	2	10329.3529	0.0014
7	3	4	6	3	3	10335.7032	-0.0049
7	2	5	6	2	4	10581.0440	0.0065
8	0	8	7	0	7	10655.8080	0.0009
5	2	4	4	1	4	10687.4016	-0.0006
8	2	7	7	2	6	11268.0880	-0.0031
6	2	4	5	1	4	11303.2430	-0.0046
6	1	5	5	0	5	11444.2188	-0.0043
5	3	2	4	2	2	11595.5432	0.0059
8	4	5	7	4	4	11637.8578	0.0090
9	1	9	8	1	8	11909.1697	0.0058
8	3	5	7	3	4	11913.7502	-0.0014
8	2	6	7	2	5	12084.4598	-0.0055
4	4	0	3	3	0	12093.0841	0.0008
6	2	5	5	1	5	12504.1015	0.0043
9	2	8	8	2	7	12604.3774	-0.0056
9	1	8	8	1	7	12835.8854	0.0090
6	3	3	5	2	3	12903.4616	0.0019
7	2	5	6	1	5	12990.1307	0.0038
9	3	7	8	3	6	13016.7555	-0.0023
10	1	10	9	1	9	13192.4550	0.0039
10	0	10	9	0	9	13202.0123	-0.0051
6	3	4	5	2	4	13395.0970	-0.0003

Table S14: Observed Transition Frequencies (in MHz) for Ar-c-^{13}CHFCH(CHF_3)O

J'	K_a'	K_c'	J''	K_a''	K_c''	Observed	Obs - Calc
9	3	6	8	3	5	13509.2485	-0.0028
5	4	1	4	3	1	13531.2129	0.0091
11	0	11	10	0	10	14478.7117	-0.0032
10	4	7	9	4	6	14579.9953	0.0089
6	4	3	5	3	3	14993.9718	-0.0002
7	3	5	6	2	5	15009.2655	-0.0003
10	3	7	9	3	6	15096.6519	0.0020
8	3	5	7	2	5	15529.5556	-0.0061
7	4	3	6	3	3	16341.5756	-0.0107
8	4	4	7	3	4	17679.1429	0.0021
9	1	8	8	0	8	17934.3212	-0.0003

Table S15: Observed Transition Frequencies (in MHz) for Ar-c-$CHF^{13}CH(CHF_3)O$

J'	K_a'	K_c'	J''	K_a''	K_c''	Observed	Obs - Calc
5	1	5	4	1	4	6733.3971	-0.0051
5	0	5	4	0	4	6868.7534	0.0054
3	2	1	2	1	1	6921.2167	-0.0115
5	3	2	4	3	1	7278.1280	0.0024
3	2	2	2	1	2	7291.0544	-0.0080
5	2	3	4	2	2	7473.5371	-0.0081
5	1	4	4	1	3	7478.5493	-0.0024
4	1	3	3	0	3	7485.0850	0.0048
6	1	6	5	1	5	8045.9019	0.0022
6	0	6	5	0	5	8141.3011	-0.0024
4	2	2	3	1	2	8305.0304	0.0019
6	2	5	5	2	4	8543.9897	0.0015
3	3	0	2	2	0	8878.9355	0.0052
6	1	5	5	1	4	8896.5365	0.0043
3	3	1	2	2	1	8897.1006	0.0145
4	2	3	3	1	3	8959.0762	-0.0004
6	2	4	5	2	3	9030.9068	-0.0028
7	1	7	6	1	6	9348.3832	0.0000
7	0	7	6	0	6	9408.5952	0.0073
5	2	3	4	1	3	9757.2189	-0.0105
7	2	6	6	2	5	9920.6835	-0.0033
7	3	5	6	3	4	10152.0755	0.0067
7	4	4	6	4	3	10163.0478	0.0052
7	1	6	6	1	5	10265.1881	0.0014
4	3	1	3	2	1	10285.0272	-0.0087
7	3	4	6	3	3	10322.6712	-0.0016
7	2	5	6	2	4	10570.1842	-0.0010
8	1	8	7	1	7	10643.3553	-0.0021
8	0	8	7	0	7	10678.5572	0.0101
5	2	4	4	1	4	10699.8224	0.0005
8	2	7	7	2	6	11278.8686	-0.0082
6	2	4	5	1	4	11309.5884	0.0011
8	1	7	7	1	6	11582.2056	0.0078
8	4	5	7	4	4	11633.1321	-0.0046
5	3	2	4	2	2	11643.0449	-0.0012
5	3	3	4	2	3	11877.3181	0.0046
8	3	5	7	3	4	11895.2212	0.0027
9	0	9	8	0	8	11952.6817	-0.0074
8	2	6	7	2	5	12076.4139	0.0009
4	4	0	3	3	0	12153.7397	-0.0046
6	2	5	5	1	5	12510.4081	0.0002
9	2	8	8	2	7	12619.0640	0.0001
9	1	8	8	1	7	12858.2937	-0.0034

Table S15: Observed Transition Frequencies (in MHz) for Ar-c-$CHF^{13}CH(CHF_3)O$

J'	K_a'	K_c'	J''	K_a''	K_c''	Observed	Obs - Calc
6	3	3	5	2	3	12952.9541	0.0037
7	2	5	6	1	5	12983.2435	0.0031
9	3	7	8	3	6	13018.5148	-0.0066
9	4	6	8	4	5	13104.8900	0.0016
10	1	10	9	1	9	13219.6781	0.0040
6	3	4	5	2	4	13429.3222	-0.0010
9	3	6	8	3	5	13486.9958	-0.0036
9	2	7	8	2	6	13539.1293	0.0014
7	1	6	6	0	6	13541.0664	-0.0087
10	2	9	9	2	8	13943.0375	-0.0002
10	1	9	9	1	8	14113.2691	-0.0012
11	1	10	10	1	9	15364.7636	-0.0003
8	1	7	7	0	7	15714.6954	0.0102

Table S16: Observed Transition Frequencies (in MHz) for Ar-c-CHFCH($^{13}CHF_3$)O

J'	K_a'	K_c'	J''	K_a''	K_c''	Observed	Obs - Calc
5	1	5	4	1	4	6723.5985	0.0020
5	0	5	4	0	4	6859.3846	-0.0015
3	2	1	2	1	1	6925.9882	0.0133
5	2	4	4	2	3	7141.8942	0.0068
5	3	3	4	3	2	7238.9401	0.0088
3	2	2	2	1	2	7297.4984	-0.0184
5	2	3	4	2	2	7467.7440	0.0016
5	1	4	4	1	3	7472.3452	-0.0131
4	1	3	3	0	3	7486.4798	0.0030
6	1	6	5	1	5	8033.9472	0.0073
6	0	6	5	0	5	8129.5918	0.0064
4	2	2	3	1	2	8308.2282	0.0020
6	2	5	5	2	4	8534.5079	0.0017
6	3	3	5	3	2	8775.5381	-0.0068
6	1	5	5	1	4	8888.5792	-0.0038
3	3	1	2	2	1	8908.9406	-0.0082
4	2	3	3	1	3	8965.1805	0.0027
7	1	7	6	1	6	9334.2168	-0.0066
5	1	4	4	0	4	9391.6751	0.0024
7	0	7	6	0	6	9394.5383	0.0023
7	2	6	6	2	5	9909.3243	-0.0092
7	3	5	6	3	4	10142.0970	0.0042
7	4	4	6	4	3	10153.2208	-0.0022
7	1	6	6	1	5	10255.1659	-0.0077
4	3	1	3	2	1	10295.1146	0.0069
7	3	4	6	3	3	10313.9422	-0.0024
4	3	2	3	2	2	10381.3290	0.0053
7	2	5	6	2	4	10562.3462	-0.0100
8	0	8	7	0	7	10662.1995	0.0030
8	2	7	7	2	6	11265.5493	0.0021
6	2	4	5	1	4	11311.1398	-0.0075
6	1	5	5	0	5	11420.8751	0.0055
8	1	7	7	1	6	11569.8608	0.0104
8	4	5	7	4	4	11622.0197	0.0071
5	3	2	4	2	2	11651.1906	0.0034
5	3	3	4	2	3	11886.8462	0.0053
9	1	9	8	1	8	11914.5387	0.0019
6	2	5	5	1	5	12516.8344	0.0040
9	2	8	8	2	7	12603.6637	0.0000
9	1	8	8	1	7	12843.4766	-0.0032
6	3	3	5	2	3	12958.9894	-0.0003
7	2	5	6	1	5	12984.9209	0.0005
9	3	7	8	3	6	13005.3643	-0.0117

Table S16: Observed Transition Frequencies (in MHz) for Ar-c-CHFCH($^{13}CHF_3$)O

J'	K_a'	K_c'	J''	K_a''	K_c''	Observed	Obs - Calc
10	1	10	9	1	9	13198.7919	0.0037
10	0	10	9	0	9	13209.2130	-0.0072
6	3	4	5	2	4	13438.0294	-0.0154
9	3	6	8	3	5	13476.9342	0.0093
9	2	7	8	2	6	13528.3119	0.0004
7	1	6	6	0	6	13546.4586	0.0009
5	4	2	4	3	2	13617.4488	0.0015
10	2	9	9	2	8	13925.4910	0.0001
10	1	9	9	1	8	14095.9896	-0.0029
11	1	10	10	1	9	15345.1049	0.0048

Table S17: Observed Transition Frequencies (in MHz) for Ar-t-CHFCH(CHF_3)O

J'	K_a'	K_c'	J''	K_a''	K_c''	Observed	Obs - Calc
8	5	3	8	4	4	5791.3819	-0.0068
4	2	3	3	2	2	5795.3029	-0.0014
7	2	6	7	1	7	5837.4969	0.0058
4	3	2	3	3	1	5949.8514	0.0006
4	3	1	3	3	0	6011.4861	-0.0029
9	2	7	9	2	8	6059.6581	0.0043
3	2	2	2	1	1	6173.7642	-0.0071
4	1	3	3	1	2	6186.1542	-0.0021
4	2	2	3	2	1	6281.4990	-0.0013
7	5	3	7	4	4	6294.9050	0.0033
6	5	2	6	4	3	6329.7592	-0.0011
11	3	8	11	2	9	6348.1555	-0.0079
5	0	5	4	1	4	6435.3523	-0.0064
5	1	5	4	1	4	6481.0326	-0.0011
5	1	4	4	2	3	6482.2406	-0.0011
5	0	5	4	0	4	6543.4683	-0.0028
5	1	5	4	0	4	6589.1467	0.0005
8	1	7	8	1	8	6647.3451	0.0121
10	2	8	10	2	9	7158.0491	0.0059
5	2	4	4	2	3	7164.8300	-0.0032
3	2	1	2	1	2	7230.7524	-0.0015
4	2	3	3	1	2	7252.6218	-0.0012
5	3	3	4	3	2	7441.2193	-0.0029
5	4	2	4	4	1	7450.7238	-0.0016
5	4	1	4	4	0	7462.2153	-0.0018
5	1	4	4	1	3	7548.7090	0.0006
9	1	8	9	1	9	7609.0860	0.0079
5	3	2	4	3	1	7634.9627	-0.0008
9	2	8	9	1	9	7643.8144	-0.0090
6	0	6	5	1	5	7688.7665	-0.0034
6	1	6	5	1	5	7706.7271	-0.0036
6	0	6	5	0	5	7734.4421	-0.0028
6	1	6	5	0	5	7752.4020	-0.0036
5	2	3	4	2	2	7917.5546	-0.0012
3	3	1	2	2	0	7968.8057	-0.0156
3	3	0	2	2	1	8044.3164	0.0070
6	1	5	5	2	4	8111.8891	0.0029
11	2	9	11	2	10	8186.0890	-0.0056
5	2	4	4	1	3	8231.2965	-0.0035
6	2	5	5	2	4	8490.3383	-0.0007
6	1	5	5	1	4	8794.4755	-0.0022
6	3	4	5	3	3	8908.1370	0.0017
7	0	7	6	1	6	8916.2553	-0.0061

Table S17: Observed Transition Frequencies (in MHz) for Ar-t-CHFCH(CHF_3)O

J'	K_a'	K_c'	J''	K_a''	K_c''	Observed	Obs - Calc
7	1	7	6	1	6	8922.9977	0.0015
7	0	7	6	0	6	8934.2183	-0.0038
6	5	2	5	5	1	8934.9539	-0.0005
6	5	1	5	5	0	8936.7449	-0.0021
7	1	7	6	0	6	8940.9568	-0.0001
7	2	5	6	3	4	8950.0362	-0.0106
6	4	3	5	4	2	8974.0537	-0.0007
6	4	2	5	4	1	9022.8600	-0.0049
6	2	5	5	1	4	9172.9329	0.0025
4	3	2	3	2	1	9298.7327	-0.0026
6	3	3	5	3	2	9332.1471	0.0018
6	2	4	5	2	3	9475.7924	-0.0042
4	2	2	3	1	3	9542.2531	0.0057
7	1	6	6	2	5	9589.6950	0.0022
4	3	1	3	2	2	9670.8083	-0.0011
7	1	6	6	1	5	9968.1443	-0.0013
8	0	8	7	1	7	10132.2814	0.0009
8	1	8	7	1	7	10134.7193	-0.0031
8	0	8	7	0	7	10139.0131	-0.0022
8	1	8	7	0	7	10141.4611	0.0040
7	2	6	6	1	5	10153.4497	0.0024
7	3	5	6	3	4	10336.2530	-0.0012
7	6	2	6	6	1	10416.5327	0.0066
7	6	1	6	6	0	10416.7656	-0.0115
7	5	3	6	5	2	10462.8995	0.0002
7	5	2	6	5	1	10472.3786	-0.0008
7	4	4	6	4	3	10497.7550	-0.0028
7	4	3	6	4	2	10644.5047	-0.0046
8	2	6	7	3	5	10851.8206	-0.0016
4	4	1	3	3	0	10907.0666	-0.0033
4	4	0	3	3	1	10919.3309	-0.0027
7	2	5	6	2	4	10923.2865	-0.0004
8	1	7	7	2	6	10944.5692	0.0049
8	2	7	7	2	6	11027.3953	0.0033
7	3	4	6	3	3	11046.9217	0.0010
8	1	7	7	1	6	11129.8646	-0.0015
8	2	7	7	1	6	11212.6941	0.0004
9	0	9	8	1	8	11343.6073	0.0078
9	1	9	8	1	8	11344.4644	0.0014
9	0	9	8	0	8	11346.0402	-0.0011
9	1	9	8	0	8	11346.9051	0.0004
6	3	4	5	2	3	11449.0348	-0.0019
5	3	2	4	2	3	11510.4698	0.0014

Table S17: Observed Transition Frequencies (in MHz) for Ar-t-CHFCH(CHF_3)O

J'	K_a'	K_c'	J''	K_a''	K_c''	Observed	Obs - Calc
8	3	6	7	3	5	11716.7849	0.0043
8	7	1	7	7	0	11897.9851	-0.0027
8	7	2	7	7	1		
8	6	3	7	6	2	11941.6358	0.0058
8	6	2	7	6	1	11943.2207	0.0091
8	5	4	7	5	3	12003.3196	0.0031
8	4	5	7	4	4	12006.6192	-0.0010
8	5	3	7	5	2	12038.9362	-0.0036
9	1	8	8	2	7	12223.3863	0.0059
8	2	6	7	2	5	12238.0333	0.0036
9	2	8	8	2	7	12258.1298	0.0042
9	1	8	8	1	7	12306.2128	0.0047
9	2	8	8	1	7	12340.9588	0.0056
8	4	4	7	4	3	12345.3701	-0.0043
5	4	2	4	3	1	12346.3039	-0.0024
5	4	1	4	3	2	12431.7002	0.0004
10	0	10	9	1	9	12553.0745	0.0066
10	1	10	9	1	9	12553.3686	0.0012
10	0	10	9	0	9	12553.9319	0.0005
10	1	10	9	0	9	12554.2329	0.0021
8	3	5	7	3	4	12702.7898	-0.0031
9	3	7	8	3	6	13047.9698	0.0009
8	3	6	7	2	5	13102.9862	-0.0018
9	8	1	8	8	0	13379.4607	-0.0087
9	8	2	8	8	1		
9	7	3	8	7	2	13419.5811	-0.0079
9	7	2	8	7	1	13419.8217	-0.0058
9	2	7	8	2	6	13434.8244	0.0045
10	1	9	9	2	8	13462.4279	0.0117
10	2	9	9	2	8	13476.3474	0.0053
9	6	4	8	6	3	13480.7877	0.0028
9	4	6	8	4	5	13484.0323	0.0039
9	6	3	8	6	2	13487.8339	-0.0042
10	1	9	9	1	8	13497.1671	0.0057
9	5	4	8	5	3	13654.6459	0.0000
6	4	3	5	3	2	13685.3906	-0.0066
11	1	11	10	1	10	13761.9010	0.0032
11	0	11	10	0	10	13762.1014	0.0064
5	5	1	4	4	0	13818.1138	0.0092
5	5	0	4	4	1	13819.7705	0.0013
9	3	7	8	2	6	13912.9405	0.0131
6	4	2	5	3	3	14013.3398	-0.0028
9	4	5	8	4	4	14104.3370	-0.0044

Table S17: Observed Transition Frequencies (in MHz) for Ar-t-CHFCH(CHF_3)O

J'	K_a'	K_c'	J''	K_a''	K_c''	Observed	Obs - Calc
9	3	6	8	3	5	14249.0386	-0.0001
10	3	8	9	3	7	14335.0087	-0.0002
10	2	8	9	2	7	14574.7365	0.0050
11	2	10	10	2	9	14688.2075	-0.0013
11	1	10	10	1	9	14696.7475	0.0062
10	3	8	9	2	7	14813.1089	-0.0074
10	4	7	9	4	6	14916.4819	0.0021
10	7	4	9	7	3	14953.5965	0.0053
12	1	12	11	1	11	14970.2544	-0.0049
12	0	12	11	0	11		
10	6	5	9	6	4	15033.3931	0.0008
10	6	4	9	6	3	15058.1707	0.0024
10	5	6	9	5	5	15089.7786	0.0047
10	5	5	9	5	4	15342.3333	-0.0013
11	3	9	10	3	8	15587.9010	-0.0018
10	3	7	9	3	6	15656.4291	0.0024
11	2	9	10	2	8	15716.2611	0.0011
10	4	6	9	4	5	15849.1723	-0.0005
12	1	11	11	2	10	15895.1378	-0.0068
12	2	11	11	2	10	15897.1763	-0.0018
12	1	11	11	1	10	15900.5366	-0.0013
13	1	13	12	1	12	16178.3967	0.0086
13	0	13	12	0	12		
11	4	8	10	4	7	16296.7090	0.0024
11	7	5	10	7	4	16501.1461	0.0064
11	7	4	10	7	3	16506.0956	-0.0007
11	6	6	10	6	5	16595.5100	0.0060
11	5	7	10	5	6	16607.7430	0.0034
11	6	5	10	6	4	16667.7184	0.0003
12	3	10	11	3	9	16817.9426	-0.0043
11	3	8	10	3	7	16916.7036	0.0033
13	2	12	12	2	11	17104.9130	-0.0088
13	1	12	12	1	11	17106.1955	-0.0095
11	5	6	10	5	5	17108.7391	-0.0037
14	1	14	13	1	13	17386.4038	0.0007
14	0	14	13	0	13		
11	4	7	10	4	6	17503.2472	-0.0017
12	4	9	11	4	8	17624.8882	0.0038
8	4	4	7	3	5	17758.8394	0.0028
12	9	3	11	9	2	17902.4723	0.0046
12	9	4	11	9	3		
13	3	11	12	3	10	18034.6602	-0.0122
12	7	6	11	7	5	18062.3944	-0.0019

Table S17: Observed Transition Frequencies (in MHz) for Ar-t-CHFCH(CHF_3)O

J'	K_a'	K_c'	J''	K_a''	K_c''	Observed	Obs - Calc
13	2	11	12	2	10	18062.5862	-0.0099
12	3	9	11	3	8	18066.1700	-0.0067
12	7	5	11	7	4	18079.0796	0.0020
12	5	8	11	5	7	18087.5024	0.0036

Table S18: Observed Transition Frequencies (in MHz) for Ar-t-^{13}CHFCH(CHF_3)O

J'	K_a'	K_c'	J''	K_a''	K_c''	Observed	Obs - Calc
4	1	3	3	1	2	6178.1534	-0.0008
4	2	2	3	2	1	6281.8586	0.0002
5	1	5	4	1	4	6465.6635	0.0007
5	0	5	4	0	4	6525.7053	0.0005
5	2	4	4	2	3	7153.4368	0.0010
5	3	3	4	3	2	7435.2418	0.0003
5	1	4	4	1	3	7533.7259	-0.0016
5	3	2	4	3	1	7636.3048	-0.0003
6	1	6	5	1	5	7687.6877	-0.0008
6	0	6	5	0	5	7713.9630	-0.0010
5	2	3	4	2	2	7915.2411	0.0004
6	2	5	5	2	4	8474.4543	0.0005
6	1	5	5	1	4	8771.6401	0.0011
6	3	4	5	3	3	8899.2725	-0.0006
7	1	7	6	1	6	8900.4824	-0.0005
7	0	7	6	0	6	8910.9808	-0.0026
6	3	3	5	3	2	9336.2011	-0.0002
6	2	4	5	2	3	9467.8774	-0.0015
7	2	6	6	2	5	9754.3071	0.0013
7	1	6	6	1	5	9939.8982	-0.0005
8	1	8	7	1	7	10108.9010	0.0015
7	3	5	6	3	4	10323.2225	0.0003
7	2	5	6	2	4	10906.4768	0.0010
8	2	7	7	2	6	11002.0426	0.0009
7	3	4	6	3	3	11049.6569	0.0002
8	1	7	7	1	6	11098.8837	-0.0003
9	1	9	8	1	8	11315.4288	0.0004
9	0	9	8	0	8	11316.8683	0.0013
8	3	6	7	3	5	11698.4631	0.0007
8	2	6	7	2	5	12209.8023	-0.0001
9	2	8	8	2	7	12228.5284	0.0015
9	1	8	8	1	7	12273.2789	0.0003
10	1	10	9	1	9	12521.1709	-0.0008
10	0	10	9	0	9	12521.6785	-0.0007
8	3	5	7	3	4	12699.1548	0.0004
9	3	7	8	3	6	13023.7119	-0.0014
9	2	7	8	2	6	13396.6360	0.0000
10	2	9	9	2	8	13442.9329	-0.0020
10	1	9	9	1	8	13462.0397	0.0005
11	1	11	10	1	10	13726.5650	0.0004
11	0	11	10	0	10	13726.7400	0.0002
9	3	6	8	3	5	14234.7009	-0.0002

Table S19: Observed Transition Frequencies (in MHz) for Ar-t-$CHF^{13}CH(CHF_3)O$

J'	K_a'	K_c'	J''	K_a''	K_c''	Observed	Obs - Calc
4	1	3	3	1	2	6177.8204	0.0020
4	2	2	3	2	1	6273.0059	-0.0007
5	1	5	4	1	4	6473.4270	-0.0026
5	0	5	4	0	4	6535.7035	-0.0019
5	2	4	4	2	3	7155.6971	0.0018
5	3	3	4	3	2	7431.4904	0.0028
5	1	4	4	1	3	7538.6694	-0.0002
5	3	2	4	3	1	7624.8424	-0.0017
6	1	6	5	1	5	7697.7621	-0.0015
6	0	6	5	0	5	7725.4014	-0.0013
5	2	3	4	2	2	7906.7728	0.0004
6	2	5	5	2	4	8479.6052	0.0004
6	1	5	5	1	4	8782.9945	-0.0017
6	3	4	5	3	3	8896.4951	-0.0003
7	1	7	6	1	6	8912.6905	0.0006
7	0	7	6	0	6	8923.8847	0.0006
6	3	3	5	3	2	9319.6418	-0.0011
6	2	4	5	2	3	9462.8789	-0.0011
7	2	6	6	2	5	9762.7550	-0.0003
7	1	6	6	1	5	9955.4058	0.0017
8	1	8	7	1	7	10123.0916	0.0022
8	0	8	7	0	7	10127.3701	0.0005
7	3	5	6	3	4	10322.7908	0.0016
7	2	5	6	2	4	10908.4633	-0.0002
8	2	7	7	2	6	11013.7235	0.0010
7	3	4	6	3	3	11031.9680	-0.0003
8	1	7	7	1	6	11115.9156	0.0008
9	1	9	8	1	8	11331.5085	-0.0011
9	0	9	8	0	8	11333.0832	0.0003
8	3	6	7	3	5	11701.5905	-0.0005
8	2	6	7	2	5	12221.5795	0.0011
9	2	8	8	2	7	12243.0787	-0.0003
9	1	8	8	1	7	12291.0202	-0.0018
10	1	10	9	1	9	12539.0982	0.0012
10	0	10	9	0	9	12539.6611	0.0021
8	3	5	7	3	4	12685.4855	-0.0007
9	3	7	8	3	6	13031.1625	0.0005
9	2	7	8	2	6	13417.0147	-0.0021
10	2	9	9	2	8	13459.9514	0.0001
11	1	11	10	1	10	13746.3118	-0.0008
11	0	11	10	0	10	13746.5076	-0.0015
9	3	6	8	3	5	14229.5921	0.0012

Table S20: Observed Transition Frequencies (in MHz) for Ar-t-CHFCH($^{13}CHF_3$)O

J'	K_a'	K_c'	J''	K_a''	K_c''	Observed	Obs - Calc
4	1	3	3	1	2	6169.7187	0.0015
4	2	2	3	2	1	6262.9924	-0.0007
5	1	5	4	1	4	6464.6658	-0.0006
5	0	5	4	0	4	6527.4941	-0.0015
5	2	4	4	2	3	7145.9587	0.0010
5	3	3	4	3	2	7420.4998	-0.0009
5	1	4	4	1	3	7529.6934	-0.0001
5	3	2	4	3	1	7612.1792	0.0009
6	1	6	5	1	5	7687.3915	-0.0014
6	0	6	5	0	5	7715.3698	0.0006
5	2	3	4	2	2	7894.8599	-0.0024
6	2	5	5	2	4	8468.4330	0.0008
6	1	5	5	1	4	8773.3804	-0.0010
6	3	4	5	3	3	8883.6867	0.0013
7	1	7	6	1	6	8900.6656	-0.0013
7	0	7	6	0	6	8912.0317	-0.0016
6	3	3	5	3	2	9303.8310	-0.0001
6	2	4	5	2	3	9449.7478	-0.0004
7	2	6	6	2	5	9750.2278	0.0002
7	1	6	6	1	5	9944.6177	0.0013
8	1	8	7	1	7	10109.3790	0.0018
8	0	8	7	0	7	10113.7374	0.0009
7	2	5	6	2	4	10894.8766	0.0021
8	2	7	7	2	6	10999.8156	0.0006
7	3	4	6	3	3	11013.9038	-0.0006
8	1	7	7	1	6	11103.3288	-0.0006
9	1	9	8	1	8	11316.0846	-0.0005
9	0	9	8	0	8	11317.6929	0.0004
8	3	6	7	3	5	11685.9900	-0.0007
8	2	6	7	2	5	12208.1514	-0.0003
9	2	8	8	2	7	12227.7030	0.0013
9	1	8	8	1	7	12276.4391	0.0003
10	1	10	9	1	9	12521.9488	0.0005
10	0	10	9	0	9	12522.5261	0.0017
8	3	5	7	3	4	12666.3374	0.0002
9	3	7	8	3	6	13014.4614	0.0017
9	2	7	8	2	6	13403.4860	-0.0011
10	2	9	9	2	8	13443.0030	-0.0014
10	1	9	9	1	8	13464.1734	-0.0011
11	1	11	10	1	10	13727.4338	-0.0002
11	0	11	10	0	10	13727.6350	-0.0009
9	3	6	8	3	5	14210.3706	-0.0003

Table S21: Principal coordinates (with their uncertainties) for the experimental structures of cFTFO and tFTFO and their argon complexes

cFTFO

	a	*da*	*b*	*db*	*c*	*dc*
C1	-1.67721	0.00176	-0.20962	0.00239	-0.48198	0.00220
C2	-0.29166	0.00794	-0.69460	0.00442	-0.62408	0.00197
C3	0.87124	0.00208	0.03029	0.00142	-0.02249	0.00132
O	-1.24571	0.00147	-1.26617	0.00144	0.30154	0.00319
H1	-2.44379	0.00183	-0.42568	0.00257	-1.21078	0.00243
H2	-0.01226	0.00859	-1.31417	0.00633	-1.46475	0.00297
F1	-1.92058	0.00181	0.94990	0.00130	0.16701	0.00123
F2	0.59190	0.00634	0.63279	0.00400	1.12740	0.00309
F3	1.88352	0.00362	-0.82860	0.00371	0.19839	0.00413
F4	1.31752	0.00343	0.95621	0.00429	-0.89191	0.00197

tFTFO

	a	*da*	*b*	*db*	*c*	*dc*
C1	-1.54313	0.00164	-0.10291	0.00885	-0.36305	0.00219
C2	-0.37160	0.00765	0.01165	0.00277	0.54496	0.00539
C3	1.00312	0.00103	-0.07636	0.00015	0.03461	0.00067
O	-1.14463	0.00137	1.17290	0.00132	0.08180	0.00270
H1	-1.47123	0.00376	-0.30067	0.01281	-1.42101	0.00233
H2	-0.48882	0.01042	-0.24495	0.00441	1.58762	0.00567
F1	-2.72524	0.00251	-0.50709	0.00432	0.15843	0.00250
F2	1.06423	0.00668	0.13785	0.00537	-1.28596	0.00103
F3	1.80398	0.00362	0.81710	0.00479	0.63104	0.00564
F4	1.50048	0.00302	-1.30051	0.00227	0.28202	0.00500

Ar-cFTFO

	a	*da*	*b*	*db*	*c*	*dc*
C1	-0.01844	0.00026	-1.57133	0.00011	-0.37447	0.00008
C2	0.70186	0.00031	-0.39052	0.00013	-0.88632	0.00002
C3	1.23429	0.00009	0.67292	0.00007	0.02217	0.00001
O	1.30241	0.00038	-1.70162	0.00012	-0.76789	0.00037
H1	-0.75515	0.00047	-2.09923	0.00025	-0.96103	0.00024
H2	0.52552	0.00062	-0.03542	0.00029	-1.89204	0.00013
F1	-0.25792	0.00030	-1.69120	0.00015	0.94961	0.00014
F2	1.64849	0.00040	0.21647	0.00024	1.19833	0.00020
F3	2.27054	0.00020	1.30089	0.00019	-0.56350	0.00024
F4	0.27837	0.00023	1.59654	0.00005	0.23557	0.00046
Ar	-2.96421	0.00005	0.44558	0.00011	-0.11401	0.00003

Table S21: Principal coordinates (with their uncertainties) for the experimental structures of cFTFO and tFTFO and their argon complexes

Ar-tFTFO

	a	*da*	*b*	*db*	*c*	*dc*
C1	-0.04240	0.00046	-1.51129	0.00020	0.35979	0.00007
C2	0.67413	0.00006	-0.57485	0.00013	-0.54561	0.00001
C3	1.30013	0.00019	0.65271	0.00019	-0.03641	0.00004
O	1.26279	0.00041	-1.83197	0.00019	-0.06217	0.00039
H1	-0.19098	0.00074	-1.33831	0.00042	1.41409	0.00014
H2	0.40996	0.00037	-0.55214	0.00025	-1.59271	0.00010
F1	-0.99040	0.00049	-2.32006	0.00034	-0.16922	0.00021
F2	1.49478	0.00023	0.60627	0.00003	1.28778	0.00003
F3	2.48775	0.00042	0.87259	0.00058	-0.61658	0.00029
F4	0.51206	0.00052	1.70805	0.00005	-0.30536	0.00046
Ar	-2.75697	0.00018	0.79925	0.00025	0.00265	0.00005
